# Competing constraints shape the nonequilibrium limits of cellular decision-making

**DOI:** 10.1073/pnas.2211203120

**Published:** 2023-03-02

**Authors:** Nicholas C. Lammers, Avi I. Flamholz, Hernan G. Garcia

**Affiliations:** ^a^Biophysics Graduate Group, University of California, Berkeley, CA 904720; ^b^Division of Biology and Biological Engineering, California Institute of Technology, Pasadena, CA 91125; ^c^Department of Physics, University of California, Berkeley, CA 94720; ^d^Institute for Quantitative Biosciences-QB3, University of California, Berkeley, CA 94720; ^e^Department of Molecular and Cell Biology, University of California, Berkeley, CA 94720; ^f^Chan Zuckerberg Biohub, San Francisco, CA 94158

**Keywords:** gene regulation, decision theory, nonequilibrium, transcriptional dynamics, cell signaling

## Abstract

Gene regulation is central to cellular function. Yet, despite decades of work, we lack quantitative models that can predict how transcriptional control emerges from molecular interactions at the gene locus. Thermodynamic models of transcription, which assume that gene circuits operate at equilibrium, have previously been employed with considerable success in the context of bacterial systems. However, the presence of ATP-dependent processes within the eukaryotic transcriptional cycle suggests that equilibrium models may be insufficient to capture how eukaryotic gene circuits sense and respond to input transcription factor concentrations. Here, we employ simple kinetic models of transcription to investigate how energy dissipation within the transcriptional cycle impacts the rate at which genes transmit information and drive cellular decisions. We find that biologically plausible levels of energy input can lead to significant gains in how rapidly gene loci transmit information but discover that the regulatory mechanisms underlying these gains change depending on the level of interference from noncognate activator binding. When interference is low, information is maximized by harnessing energy to push the sensitivity of the transcriptional response to input transcription factors beyond its equilibrium limits. Conversely, when interference is high, conditions favor genes that harness energy to increase transcriptional specificity by proofreading activator identity. Our analysis further reveals that equilibrium gene regulatory mechanisms break down as transcriptional interference increases, suggesting that energy dissipation may be indispensable in systems where noncognate factor interference is sufficiently large.

## Significance

It is well established that the eukaryotic transcriptional cycle contains molecular reactions that consume biochemical energy in the form of ATP, yet the impact of these energy-consuming processes on how gene loci sense and respond to cognate transcription factor concentrations remains poorly understood. In this paper, we derive simple models of gene regulation to investigate how energy consumption impacts the rate at which genes can transmit information and drive cellular decision-making. We find that energy can accelerate information transmission by orders of magnitude and predict that gene loci will harness energy in different ways depending on the level of interference from the binding of noncognate transcription factors at the gene locus.

Throughout biology, systems must make accurate decisions under time constraints using noisy molecular machinery. Eukaryotic gene regulation exemplifies this challenge: genes must read out input concentrations of transcription factor proteins and respond by producing appropriate levels of gene product (mRNA and eventually protein) to drive downstream cellular decisions. Interestingly, the gene activity underlying cellular decision-making is often subject to large amounts of noise. Indeed, experiments across a wide range of organisms have revealed that eukaryotic transcription is highly stochastic, occurring in episodic bursts (1, 2)—periods of activity interspersed with periods of transcriptional silence—that unfold over timescales ranging from minutes to hours (3). Because of this stochasticity, the transcription rate is a noisy reflection of transcription factor concentration. Over time, the accumulation of gene products tends to average out this noise, but biological processes must operate under time constraints: Cells in developing fruit fly embryos have only minutes to determine their developmental fates (4, 5), antigen recognition in T-cells unfolds over a single day (6), and cells in adult tissues are constrained by mRNA half-lives that range from minutes to days (7).

A key question, therefore, is how the molecular architecture of gene loci—the number and identity of biochemical steps in the transcriptional cycle and the reaction rates connecting these steps—dictates the amount of time needed for bursty gene expression to drive accurate cellular decisions. In particular, while it is widely accepted that processes within the eukaryotic transcriptional cycle consume biochemical energy (8, 9), we do not yet know what nonequilibrium should “look like” in the context of transcriptional systems. Indeed, it remains challenging not only to predict unambiguous signatures of energy expenditure that can be detected experimentally (10–12) but also to establish how energy consumption can be harnessed to improve gene regulatory performance in the first place (13).

**Fig. 1.**
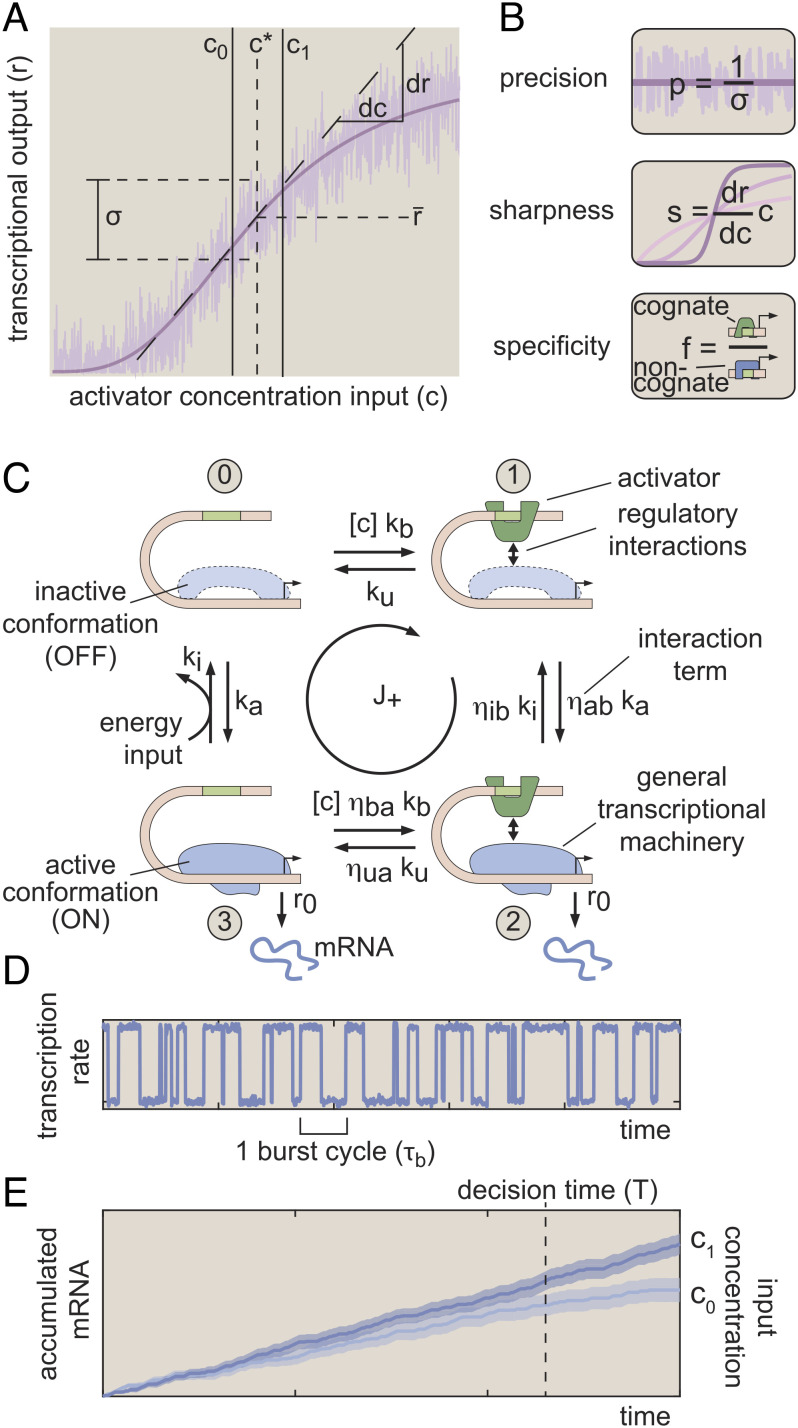
Three regulatory features shaping transcriptional information transmission. (*A*) Gene regulatory input–output function illustrating the basic biological problem considered in this work. Here, a cell must distinguish between two activator concentrations, *c*_0_ and *c*_1_, based on the transcriptional output of a gene locus (purple curve). (*B*) We examine how three regulatory features of the transcriptional input–output function—sharpness, precision, and specificity—dictate the rate at which the transcriptional output drives biological decisions. (*C*) Four-state MWC-like model of transcription used as the foundation of our investigations accounting for activator binding and where mRNA production occurs when the gene locus switches to its active (ON) conformation. A hypothetical energy input is depicted along the rate from state 3 to state 0. In practice, our framework permits nonequilibrium driving to occur along any of the eight transition rates in the model. (*D*) Simulated burst dynamics for one realization of the model shown in (*C*). The burst cycle time is defined as the average time required to complete one ON → OFF → ON cycle and sets the timescale over which biological decisions unfold. (*E*) Illustrative simulation results for accumulated mRNA levels driven by *c*_1_ and *c*_0_. Solid lines show trajectories for a single locus, and shaded regions indicate the SD of levels taken across 100 simulated trajectories. The vertical dashed line indicates the “decision time,” when the expected mRNA levels driven by *c*_1_ and *c*_0_ are sufficiently different to permit an accurate decision about the input activator concentration.

Here, we use concepts from information theory and statistical physics as a lens to investigate how energy dissipation impacts the timescale on which gene circuits can drive cellular decisions. We consider a simple binary choice scenario wherein a cell must decide, as rapidly as possible, whether it is subjected to a high (*c*_1_) or low (*c*_0_) concentration of a transcriptional activator based on the transcriptional output of a gene locus. The basis for this decision is the gene’s input–output function (Fig. 1*A* and *B*), which emerges from microscopic interactions between input activator molecules and their target gene loci (Fig. 1*C*) that induce differences in the output dynamics of transcriptional bursting (Fig. 1*D*) for high and low activator concentrations. In turn, these differences in burst dynamics drive different rates of mRNA accumulation (Fig. 1*E*). Because each ON/OFF fluctuation is stochastic, the resulting gene expression levels are noisy, and the cell must wait for some time *T* before it is possible to accurately distinguish between *c*_1_ and *c*_0_. Our central question in this work is whether energy dissipation within the molecular processes driving transcription allows gene loci to decrease the decision time, *T*, and, if so, how this performance gain manifests in terms of measurable features of the transcriptional input–output function.

There are multiple ways in which energy dissipation could alter the input–output behavior of a gene locus to improve cellular decision-making. As illustrated in Fig. 1*A* and *B*, nonequilibrium processes could increase sensitivity to differences in input transcription factor concentration (“sharpness”) or suppress transcriptional noise (“precision”). Since our model assumes that, in addition to the concentration of the cognate activator, *C*, the gene locus is subject to some level of noncognate factors, *W*, energy dissipation could also buffer against interference from off-target activation (“specificity”).

Recent works have begun to uncover a complex space of tradeoffs among these three aspects of transcriptional performance both at and away from thermodynamic equilibrium. A recent study found that systems operating at thermodynamic equilibrium suffer from strict tradeoffs between transcriptional specificity and transcriptional precision (14) but that this tradeoff can be overcome by gene circuits that spend energy to enhance specificity through a scheme reminiscent of classical kinetic proofreading (15, 16). Similarly, a separate study demonstrated that energy dissipation can enhance transcriptional sharpness (17). Interestingly, while energy can increase sharpness and specificity separately, another study found that nonequilibrium levels of specificity come at the cost of suboptimal sharpness (18). The authors also found that energy dissipation tends to decrease transcriptional precision, although this conclusion likely hinges on the study’s modeling assumptions (18). Despite this progress, it remains unclear how these nonequilibrium gains and tradeoffs ultimately impact how effectively gene circuits can harness differences in transcription factor concentrations to drive cellular decisions.

In this work, we identify a key quantity, the rate of information transmission (IR) from input transcription factor concentrations to output transcription rates as the quantitative link between energy-dependent changes in the transcriptional input–output function (Fig. 1*B*) and the speed at which gene loci drive accurate biological decisions (Fig. 1*E*) (5, 20). We use this rate as a quantitative measure to examine the interplay between energy dissipation and cellular decision-making. We consider model gene circuits with varying numbers of activator binding sites. We also examine models with different numbers of molecular steps in the activation pathway since transcription is also thought to require multiple molecular steps beyond activator binding itself, such as the localization of key general transcription factors to the gene locus (21).

We demonstrate that energy dissipation increases the rate at which genes can drive cellular decisions for all models considered. Additionally, we find that while energy input can drive increases in all three regulatory features considered (sharpness, precision, and specificity; Fig. 1*B*), genes cannot realize these gains simultaneously. In particular, we show that the upper limit of information transmission is defined by a shifting tradeoff between sharpness and specificity that is defined by the relative concentration of wrong-to-right activator species.

In closing, we identify hallmarks of nonequilibrium gene regulation that may be amenable to experimental detection. We also demonstrate the importance of using theoretical models that account for noncognate factor binding when interpreting experimental measurements of gene expression. Altogether, this work provides a rigorous foundation for interrogating the role of energy dissipation in eukaryotic gene circuit regulation.

## 1. Results

### A Simple Model for Probing the Interplay Between Energy and Information in Transcription.

A.

We sought to establish gene circuit models that capture two essential characteristics of eukaryotic transcription. First, gene regulation hinges upon interactions between specific and general transcription factors. Although salient regulatory information tends to reside exclusively in a few specific transcription factors targeted to binding sites within enhancers (22), these proteins are not sufficient to give rise to transcription. Instead, transcription and transcriptional control depend on interactions between specific regulatory factors and other key molecular players at the gene locus, such as mediators (18, 23), RNA polymerase (24), nucleosomes (14, 25), and various subunits of the preinitiation complex (21). While these factors do not themselves carry information about the input activator concentration, they constitute key molecular steps within the transcriptional cycle. This multiplicity of molecular players implies that gene loci may exist in multiple distinct molecular states corresponding to different binding configurations of specific and general molecules (e.g., ref. 26). Moreover, some of these processes—e.g., nucleosome displacement (27), preinitiation complex assembly (28), and RNA polymerase initiation (29)—entail the dissipation of biochemical energy, opening the door to nonequilibrium behaviors.

Second, it has recently become apparent that eukaryotic transcription is characterized by stochastic, episodic bursts of activity interspersed with periods of transcriptional silence (1–3). Since the concentration of specific transcription factors can regulate burst dynamics (30–32), a simple model would suggest that transcriptional bursts originate from the binding and unbinding of specific transcription factors. Although this may be the case in some yeast genes (33), recent in vivo measurements in other eukaryotic systems have revealed that activators and repressors typically bind DNA for seconds, rather than minutes or hours (2, 30). This temporal disconnect between bursting and transcription factor binding suggests a model in which transcriptional burst cycles—corresponding to OFF → ON → OFF fluctuations in the locus conformation (Fig. 1*D*)—are not determined by transcription factor binding alone, but entail additional molecular reactions that are decoupled from the timescale of activator binding.

Together, these observations support a Monod–Wyman–Changeux (MWC)-like framework (14, 18, 19, 25) for modeling transcription wherein specific transcription factors act as effector molecules, conditioning the frequency with which the gene locus fluctuates between active and inactive transcriptional conformations. The simplest model that meets this description is one where a transcriptional activator binds to a single binding site at the gene locus and where a second molecular reaction dictates fluctuations between two conformations: an inactive (OFF) state where no mRNA is produced and a transcriptionally active (ON) state where mRNA is produced at rate *r*_0_.

If we neglect the binding of noncognate transcription factors, this leads to the model shown in Fig. 1*C*. This model contains four basal reaction rates: the transcription factor binding and unbinding rates (*k*_b_ and *k*_u_) and the locus activation and deactivation rates (*k*_a_ and *k*_i_). We leave the molecular identity of this locus activation step unspecified, but in principle, it may reflect a conformational change in any of the elements of the general transcriptional machinery mentioned above. In addition to these basal rates, the *η* terms in Fig. 1*C* capture interactions between the molecular components that make up the gene circuit. Here, the first subscript indicates which molecular reaction the *η* term modifies (binding or unbinding; activation or inactivation of the general transcriptional machinery), and the second subscript indicates the molecule performing the modification (bound activator “b” or activated transcriptional machinery “a”). For instance, *η*_ab_ encodes the degree to which the rate of locus activation is modified by having a transcription factor bound at the locus (*η*_ab_ > 1 corresponds to an activating transcription factor). Lastly, the average rate of mRNA production in this model is simply equal to $\bar{r}=r_{0}\left(\pi_{2}+\pi_{3}\right)$, where *π*_*i*_ is the steady-state probability of finding the system in state *i*.

### B. Calculating Energy Dissipation Rates and Decision Times.

At equilibrium, all state transitions in our model must obey the law of microscopic reversibility. Energy dissipation along one or more of the microscopic transitions shown in Fig. 1*C* lifts this strict equilibrium constraint and opens the door to novel forms of nonequilibrium gene regulatory logic. For the model shown in Fig. 1*C*, the energy dissipated per unit time (*Φ*) can be expressed as

[1] $$\begin{array}{c} \Phi =J\ln\frac{\eta_{\;\text{ab}}\eta_{\;\text{ua}}}{\eta_{\;\text{ib}}\eta_{\;\text{ba}}}, \end{array}$$

where the *η* terms are defined in Fig. 1*C* and the net probability flux, ***J*** (defined in *SI Appendix*, Eq. S5), encodes the degree to which microscopic transitions in the system are biased in the clockwise (***J*** > 0) or counterclockwise (***J*** < 0) direction (34). *SI Appendix*, Appendix A.2 for further details. *Φ* is a strictly positive quantity with units of k_B_T per unit time that indicates how “near” or “far” a system is from thermodynamic equilibrium (34, 35). For ease of comparison across different realizations of our model gene circuit, we express *Φ* in units of k_B_T per burst cycle (“energy per burst”). We note that all time-dependent quantities reported throughout this work will, likewise, be given in burst cycle units (*SI Appendix*, sections A.5 and A.6 for details).

Our central aim is to understand how energy dissipation impacts the rate at which gene loci transmit information and drive cellular decisions. For simplicity, we assume that *c*_0_ and *c*_1_ are constant over time. We also stipulate that the difference between these concentrations (*δc*) is relatively small, such that *δc* = *c*_1_ − *c*_0_ = 0.1c^*^, where c^*^ is the midpoint concentration c^*^ = (*c*_1_ + *c*_0_)/2. This value of *δc* is equivalent, for example, to concentration differences for the activator Bicoid between adjacent nuclei in early fruit fly development (36). Finally, throughout this work, we measure all concentrations in units of c^*^. Fig. 1*E* shows the predicted integrated transcriptional output of a gene locus when it is exposed to high or low activator concentrations. Intuitively, it should be easier to distinguish between these two scenarios when i) the difference between average transcript production rates (slope of the lines in Fig. 1*E*) is large or ii) the noise (shaded regions) in the accumulated output is small.

IR codifies this intuition, providing a quantitative measure of a gene’s ability to read out and respond to different input activator concentrations. Formally, IR is defined as the rate of change in the Kullback–Leibler divergence (37) between our two hypotheses (*C* = *c*_0_ and *C* = *c*_1_) given the expected transcriptional output of our model gene circuit. If we take the noise in the transcriptional output to be approximately Gaussian (*SI Appendix*, Appendix B), IR can be expressed as

[2] $$\begin{array}{c} \;\text{IR}=\frac{1}{2}\underset{\;\text{input}}{\underbrace{(\frac{\delta c}{c^{*}}{)}^{2}}}\times \underset{\;\text{output}}{\underbrace{\;s^{2}p^{2}}}, \end{array}$$

where IR is strictly positive and has units of information per unit time, and *s* and *p* are the sharpness and precision of the transcriptional response, respectively, as defined in Fig. 1*B*. *SI Appendix*, Appendix C for a full derivation of this expression. We note that the native units of Eq. **2** are natural log units (“nats”). For simplicity, we give all informational quantities in the more familiar “bits,” such that IR has units of bits per burst cycle (“bits per burst”). Additionally, the precision term, *p*, pertains solely to noise from intrinsic fluctuations between microscopic states at the gene locus and does not account for Poisson noise resulting from mRNA synthesis, which is expected to be small relative to the noise from locus fluctuations (*SI Appendix*, Appendix D for details).

Eq. **2** contains two terms: an input component that encodes the size of the activator concentration gradient and an output component that depends on the sharpness and precision of the transcriptional input–output function (Fig. 1*A* and *B*). This expression provides quantitative support for the intuitions outlined above. IR can be increased both by increasing the difference between the transcription rates driven by *c*_1_ and *c*_0_ (i.e., increasing the sharpness) and by decreasing the noise level (i.e., increasing precision). Moreover, since both *s* and *p* can be calculated analytically from the microscopic reaction rates in our gene circuit (*SI Appendix*, Appendix A.3), Eq. **2** allows us to calculate and compare information rates for gene circuits with different microscopic reaction rates.

The IR, in turn, dictates how rapidly cells can distinguish between the two activator concentrations, *c*_0_ and *c*_1_, based on the accumulated transcriptional output of a gene circuit. Previous works (5, 20) have established that the theoretical lower limit for the time required to distinguish between *c*_0_ and *c*_1_ is given by

[3] $$\begin{array}{c} \bar{T}=\ln(\frac{1-\varepsilon }{\varepsilon })\frac{1-2\varepsilon }{\;\text{IR}}, \end{array}$$

where *ε* is the probability of being wrong, i.e., choosing *c*_1_ when the true value is *c*_0_ (or vice versa) (*SI Appendix*, Appendix E and (5) for details). We note the error-tolerance *ε* in Eq. **3** is extrinsic to the gene circuit model and depends on the nature of the downstream cellular processes. Unless otherwise noted, we follow (5) and set *ε* = 0.32, equivalent to an error level of “1 sigma.” Finally, we note that all calculations for decision times an related quantities assume that microscopic reactions within the gene circuit have reached steady state; an approach that is well justified for the decision timescales considered (*SI Appendix*, Appendix F).

### C. Energy Dissipation Increases the Rate of Information Transmission.

Utilizing our framework, we investigated whether increasing the energy dissipated by our model gene circuit, *Φ*, increases the rate at which this circuit drives cellular decisions between *c*_0_ and *c*_1_. We expanded methods employed in refs. 12, 17 to develop an algorithm capable of systematically exploring how different transition rates dictate gene circuit features. This algorithm can determine the maximum IR achievable by different realizations of our gene circuit as a function of energy dissipation. *SI Appendix*, Appendices G and H for details regarding its implementation and validation.

**Fig. 2.**
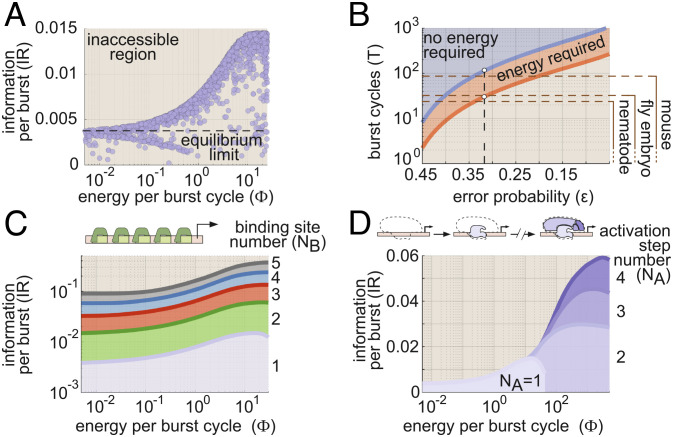
Energy dissipation increases the information transmission rate in gene circuits. (*A*) Parameter sweep exploring the range of possible model performance for information rate (IR from Eq. **2**) as a function of energy dissipation rate (*Φ* from Eq. **1**). (*B*) The amount of time needed to distinguish between *c*_0_ and *c*_1_ as a function of the probability of deciding incorrectly for equilibrium and nonequilibrium gene circuits. The decision time is given in terms of the number of transcriptional burst cycles required for a decision to be made. Achievable decision times for equilibrium and nonequilibrium are indicated as shaded regions. Note that the x-axis is arranged in order of decreasing error probability (i.e., increasing accuracy) from left to right. Horizontal lines indicate approximate upper bounds on decision times (in burst cycles) for different organisms. (*C*) Parameter sweep results for achievable IR and *Φ* values (shaded regions) for gene circuits with 1 to 5 activator binding sites. (*D*) Sweep results illustrating achievable IR vs. *Φ* regimes for gene circuits featuring 1 to 4 molecular activation steps. (For all parameter sweep results in *A*–*D*, transition rate and interaction term magnitudes, *k* and *η*, were constrained such that 10^−5^ ≤ *kτ*_*b*_ ≤ 10^5^ and 10^−5^ ≤ *η* ≤ 10^5^, where *τ*_*b*_ is the burst cycle time. *η*_*ab*_ and *η*_*ib*_ were further constrained such that *η*_*ab*_ ≥ 1 and *η*_*ib*_ ≤ 1, consistent with our assumption that the transcription factor activates the gene locus. Note that we apply these same parameter bounds for all subsequent sweeps presented throughout the main text.)

Fig. 2*A* shows the relation between IR and *Φ* resulting from our numerical analysis. Here, each circle represents IR and *Φ* values for a single realization of our gene circuit (Fig. 1*C*), as defined by its complement of transition rate values. Near equilibrium, our analysis reveals that gene circuits can transmit information no faster than 0.035 bits per burst (far left-hand side of Fig. 2*A*). According to Eq. **3**, this means that the best equilibrium gene circuits require at least 110 burst cycles to drive a decision between concentrations *c*_1_ and *c*_0_ with an error probability of 32% when these concentrations differ by 10% (Fig. 2*B*). In the developing fruit fly embryo (*D. melanogaster*), where the burst timescale (*τ*_*b*_) is approximately 2 min (3), this translates to a decision time of 3.7 h, far too long to meet the time constraints imposed by early nuclear cleavage cycles, 8–60 min, (4). Our equilibrium gene circuit would require even longer times in adult nematode (*C. elegans*) and mouse (*M. musculus*) cells, where *τ*_*b*_ is much higher, with measurements ranging from 61 to 105 min, $\bar{T}\geq 112$ h, (38) and 30 min to multiple hours, $\bar{T}\geq 55$ h, (30), respectively. In each case, these timescales likely exceed decision time limits imposed by mRNA decay or cellular division times, which set upper limits on the time over which gene output can be averaged (horizontal lines in Fig. 2*B* and *SI Appendix*, Appendix I for further details).

Our analysis indicates that energy dissipation opens the door to improved information transmission, leading to a fourfold increase in the upper IR limit from 0.0035 to 0.014 bits per burst cycle (Fig. 2*A*). Moreover, this performance gain is realized at biologically plausible levels of energy consumption: IR reaches its maximum nonequilibrium value at *Φ* ≈ 20 k_B_T per cycle, which is approximately equivalent to the hydrolysis of one to two ATP molecules (39). This corresponds to an energy-dependent decrease in decision time from 110 to 29 burst cycles (red shaded region in Fig. 2*B*). This reduction meets the upper decision limit for mouse cells (Fig. 2*B*). Yet, there remains an absolute speed limit that no amount of energy dissipation can overcome, as shown by the empty space below the red nonequilibrium boundary in Fig. 2*B*.

How can gene circuits do better? Real transcriptional systems are typically far more complex than the simple four-state model in Fig. 1*C*; gene enhancers typically feature multiple transcription factor binding sites (22), and transcriptional activation depends on the combined action of multiple general transcription factors at the gene locus (3). Thus, to overcome this speed limit, we must examine the impact of tuning two molecular “knobs”: the number of specific activator binding sites in our model (N_B_) and the number of molecular steps required to achieve productive transcription (N_A_). For simplicity, we focus on systems in which all binding sites are identical and assume identical kinetics for all molecular transitions between locus conformations. While restrictive, this simple approach gives rise to rich, biologically salient behaviors. While we explore the effects of varying N_B_ and N_A_ separately, these mechanisms are mutually compatible and may act jointly in real biological systems. *SI Appendix*, Appendix J for details regarding the implementation of these higher-order models.

#### Adding binding sites improves information-energy tradeoffs.

We first examined the performance of gene circuit models with multiple binding sites. In these models (as with the four-state model described above), activator binding does not directly dictate transitions in and out of transcriptionally active molecular states, but instead increases or decreases the likelihood of these transitions. Models with multiple binding sites also permit pairwise cooperative interactions between activator molecules, encoded by *η*_*ub*_ terms (*SI Appendix*, Appendix J and Fig. S15*A*). With these assumptions, we employed our parameter sweep algorithm to explore tradeoffs between the rate of energy dissipation (*Φ*) and the IR for systems with 1 to 5 activator binding sites. *SI Appendix*, Appendix A.2 for details about how we extend Eq. **1** to calculate *Φ* for higher-order models. In all cases, we held the number of activation steps constant at N_A_ = 1 (as in Fig. 1*C*).

As illustrated in Fig. 2*C*, adding activator binding sites shifts the IR vs. *Φ* tradeoff boundary from Fig. 2*A* upward, allowing for higher information transmission rates for a given energy dissipation rate. This leads to significant IR gains, even in gene circuits operating near the equilibrium limit (left-hand side of Fig. 2*C*), with the upper equilibrium limit increasing by approximately a factor of 25 from 0.0035 bits per burst cycle for N_B_ = 1 to 0.090 bits per cycle for N_B_ = 5. As a result, gene circuits with five binding sites need as little as five burst cycles to distinguish between *c*_1_ and *c*_0_ in the absence of any energy dissipation, easily satisfying the decision time constraints of the biological systems shown in Fig. 2*B*. More generally, we find that the lower decision time limit for equilibrium circuits scales as the inverse of the number of binding sites squared ($\bar{T}∼{\;{\text{N}}_{\;\text{B}}}^{-2}$, *SI Appendix*, Fig. S1*A*).

#### Adding molecular activation steps allows gene circuits to harness higher rates of energy dissipation.

Next, we expanded the four-state model by changing the number of activation steps (1 ≤ N_A_ ≤ 4) while holding the number of binding sites fixed at N_B_ = 1 (Fig. 2*D*, *Top*). To illustrate this model, let us first consider the baseline case, where N_A_ = 1. Here, locus activation depends on the state of a single molecular component (e.g., mediator), which can be disengaged (i.e., the locus is OFF) or engaged (i.e., the locus is ON). Now, consider a model in which locus activation also depends on the state of a second molecular component (e.g., PIC assembly) that can, likewise, be either engaged or disengaged. If we stipulate that both components must be engaged to achieve RNA polymerase initiation, then two molecular activation steps are required to reach the ON state and N_A_ = 2. We use the same logic to extend the model to the N_A_ = 3 and N_A_ = 4 cases to capture the impact of the additional molecular components necessary for transcription. *SI Appendix*, Appendix J and Fig. S15*B* for details.

We conducted parameter sweeps to examine the interplay between energy dissipation and information transmission for these systems. As with adding binding sites, the addition of activation steps leads to increased rates of information transmission. Unlike increasing N_B_, however, these IR gains do not come for free. Instead, the addition of activation steps extends the *Φ*-IR boundary into higher-energy regimes such that, for nonequilibrium gene circuits to achieve larger gains in IR, they must do so at the expense of increased energy dissipation rates (Fig. 2*D*).

This increased IR gain means that systems with multiple activation steps can drive decisions between *c*_1_ and *c*_0_ more rapidly than the simple four-state gene circuit. For example, nonequilibrium gene circuits with four activation steps can drive decisions nearly four times as rapidly as systems with a single step (8 vs. 29 burst cycles; *SI Appendix*, Fig. S1*B*). This 8-burst-cycle limit approaches what can be achieved by an equilibrium gene circuit with five activator binding sites (five burst cycles; compare *SI Appendix*, Fig. S1 *A* and *B*), suggesting a similarity between adding activator binding sites at equilibrium and adding activation steps out of equilibrium. However, this parity has an energetic cost: to approach the performance of the equilibrium five-binding-site model, the nonequilibrium one-binding-site system with five conformations must dissipate at least 180 k_B_T per burst.

### D. Increases in Nonequilibrium Sharpness Improve Information Transmission.

According to Eq. **2**, the energy-dependent increases in IR uncovered in Fig. 2 must result from increased sharpness, increased precision, or some combination thereof. Thus, to uncover how energy reshapes the transcriptional input–output function to increase IR, we used our numerical sweep algorithm to examine the space of achievable sharpness and precision values for our baseline four-state model (Fig. 1*C*) both at and away from thermodynamic equilibrium. One challenge in comparing sharpness and precision levels across different gene circuits is that the upper bounds on both *s* and *p* depend on the transcription rate at c^*^, $\bar{r}=r_{0}\pi_{\text{a}}$ (Fig. 1*A*), where *π*_a_ is the fraction of time that the system spends in transcriptionally active states. This makes it difficult to compare the sharpness and precision of gene circuits with different transcription rates. Thus, for ease of comparison across different model realizations, we give all results in terms of normalized sharpness and precision measures: S and P, where S = *s*/(*π*_a_(1 − *π*_a_)) and P = *p*(*π*_a_(1 − *π*_a_)). These metrics adhere to consistent bounds irrespective of the activity level and have intuitive interpretations. For instance, the S value of a particular gene circuit’s input–output function gives the Hill coefficient of an equivalently sharp Hill function. *SI Appendix*, Appendix K for details.

**Fig. 3.**
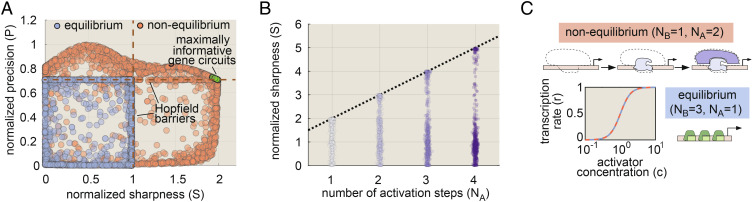
Increased transcriptional sharpness drives increased information transmission away from equilibrium. (*A*) Scatter plot of parameter sweep results showing the normalized sharpness and precision for equilibrium and nonequilibrium gene circuits. The absence of gene circuits in the upper right quadrant indicates that nonequilibrium circuits cannot simultaneously maximize sharpness and precision. (*B*) Plot of achievable nonequilibrium sharpness levels for models with 1 to 4 molecular activation steps and one activator binding site. Each circle represents a single gene circuit model. Normalized sharpness is bound by the number of locus conformations. (*C*) Cartoon illustrating functional equivalence between three binding sites at equilibrium and two activation steps out of equilibrium. The plot shows input–output functions for maximally sharp realizations of each case, demonstrating the equivalent sharpness levels driven by the two strategies.

Fig. 3*A* shows the results of our analysis, with each circle representing the S and P values for a single gene circuit realization. For systems operating at equilibrium (blue dots in Fig. 3*A*), we find that both S and P are bound by “Hopfield barriers” (dashed lines) (16, 17) with values of 1 and $1/\sqrt{2}$, respectively. These bounds place strict limits on information transmission at equilibrium and have a straightforward interpretation: they are precisely equal to the sharpness and precision of a simple two-state gene circuit with a single activator binding site and no molecular activation step (*SI Appendix*, Appendix L).

Energy dissipation permits gene circuits to overcome these equilibrium performance bounds, increasing S by up to a factor of 2 and P by up to a factor of $\sqrt{2}$ with respect to their equilibrium limits (Fig. 3*A*). Yet, while energy can improve sharpness and precision individually, the absence of realizable gene circuits in the upper-right-hand corner of Fig. 3*A* indicates that genes cannot maximize both simultaneously. This tradeoff places inexorable limits on the degree to which energy can boost IR and arises because maximally sharp and maximally precise gene circuits require distinct and incompatible underlying molecular architectures (*SI Appendix*, Appendix M for details).

Because sharpness and precision cannot be maximized simultaneously, gene circuits that dissipate energy must “choose” which aspect to maximize. From the perspective of IR maximization, the choice is clear: Fig. 3*A* shows the location of 100 gene circuits within 1% of the maximum of 0.014 bits per cycle (Fig. 2*A*) in S − P phase space (green circles). The clustering of all these circuits in the plot reveals that the most informative gene circuits maximize transcriptional sharpness (S = 2) at the cost of retaining equilibrium precision levels ($\;\text{P}=1/\sqrt{2}$). As with the equilibrium case, these values have an intuitive interpretation: They are simply equal to the expected sharpness and precision of a two-state system, one in which both the ON and OFF rates are concentration-dependent (*SI Appendix*, Appendix N). Thus, although spending energy to overcome the constraints of detailed balance opens up a vast new space of possible regulatory schemes, maximally informative nonequilibrium gene circuits exhibit an emergent simplicity, converging upon architectures in which their many molecular degrees of freedom collapse into a few effective parameters that define system behavior.

#### Nonequilibrium gains in sharpness drive IR increases in more complex regulatory architectures.

To assess the generality of our results, we used our parameter sweep algorithm to examine equilibrium and nonequilibrium tradeoffs between sharpness and precision for more complex gene circuits with 2 to 5 activator binding sites and 2 to 4 molecular activation steps. In all cases, energy dissipation increases the upper limits of S and P, and as with our simple four-state model, these nonequilibrium performance gains cannot be realized simultaneously (*SI Appendix*, Fig. S2 *A* and *B*). For all models considered, the gains in IR uncovered in Fig. 2 are maximized by spending energy to increase sharpness, rather than precision (*SI Appendix*, Appendix O for further details). For the case of multiple activator binding sites (N_B_ > 1), the N_B_-dependent increases in IR shown in Fig. 2*C* arise because increasing the number of binding sites increases the upper sharpness limit both at and away from equilibrium, (*SI Appendix*, Fig. S2 *A* and *C* and Appendix O; (17, 18)).

More surprisingly, we find that increasing the number of molecular conformations (N_A_) while holding the number of activator binding sites can also increase transcriptional sharpness in systems operating out of equilibrium. Fig. 3*B* shows the range of achievable S values for nonequilibrium systems as a function of N_A_. The upper S limit scales linearly with N_A_, such that S_neq_ ≤ N_A_ + 1. This linear scaling is identical to the effect of adding activator binding sites at equilibrium, where S_eq_ ≤ N_B_ (*SI Appendix*, Fig. S2*C*), providing intuition for why systems with multiple molecular steps can drive faster decisions: With respect to transcriptional sharpness, the regulation of multiple activation steps by a single binding site in a nonequilibrium gene circuit is functionally equivalent to the effect of having multiple binding sites at equilibrium (Fig. 3*C*).

### E. Energy Dissipation Is Required for Rapid Cellular Decisions at High Noncognate Factor Concentrations.

In real biological settings, cells do not contain only a single species of transcription factor, but many. Therefore, to drive timely biological decisions, a gene circuit must not only sense and respond to its cognate transcription factor, but also efficiently filter out “irrelevant” signals from noncognate factors. This process is inherently challenging in eukaryotes, where short DNA-binding footprints lead to modest energetic differences between specific (correct) and nonspecific (incorrect) transcription factor binding events on the order of 4.6 k_B_T (40), meaning that noncognate transcription factors unbind from gene loci approximately 100-fold faster than cognate factors (see Box 1 for further discussion).

**Box 1: Modeling transcription factor competition**

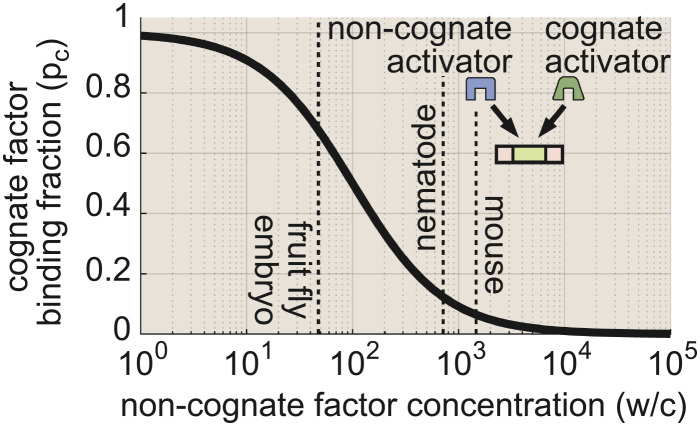

In eukaryotes, transcription factors tend to have short DNA-binding footprints, which means that cognate factors stay bound to their target sites only about 100 times longer than noncognate factors (40). To gain intuition for whether a 100-fold difference in binding kinetics is sufficient to drive biological decisions, we can examine a stripped-down scenario in which cognate and noncognate activators must compete to bind a single binding site. We quantify the severity of noncognate factor interference by calculating the fraction of total binding interactions that reflect the action of cognate (rather than noncognate) factors, which is given by

[B.1] $$\begin{array}{c} p_{c}=\frac{\pi_{c}}{\pi_{c}+\pi_{w}}=\frac{f}{f+\frac{w}{c}}. \end{array}$$

Here, *w*/*c* is the ratio of noncognate to cognate factor concentrations and *f* is the transcriptional specificity, which is defined as the (average) ratio of the probability of having cognate (*π*_*c*_) and noncognate (*π*_*w*_) factors bound, normalized by the concentration, namely

[B.2] $$\begin{array}{c} f=\frac{w}{c}\frac{\pi_{c}}{\pi_{w}}. \end{array}$$

We note that Eq. **B.2**, which considers competition between two activator species to bind and activate a single gene, is distinct from and complements specificity definitions employed in previous works, which examine the problem for a single activator species that regulates a cognate and a noncognate locus (14, 18) (*SI Appendix*, P.1 for details). From Eq. **B.1**, we see that *f* sets the scale for the severity of noncognate factor interference. At equilibrium, *f* is constrained to be equal to the ratio of wrong-to-right unbinding rates, *α* = *k*_u_^*w*^/*k*_u_ (*SI Appendix*, Appendix P.2), such that

[B.3] $$\begin{array}{c} p_{c}=\frac{\alpha }{\alpha +\frac{w}{c}}. \end{array}$$

Eq. **B.3** indicates that cognate factor binding will dominate when *w*/*c* < *α*, while noncognate factors dominate when *w*/*c* > *α*. For concreteness, we set *α* = 100 throughout this work.

Where do actual biological systems fall? The dashed lines on the plot above indicate where actual biological systems fall along the *w*/*c* axis. A recent study pursuing synthetic enhancer design in the early fly embryo cited 47 pertinent regulatory factors that were accounted for to avoid off-target binding (22), leading to an estimate of *w*/*c* = 47 (see also ref. 42). Inserting this value into Eq. B.3, we find that *p*_*c*_ ≈ 2/3 in the fly embryo. To survey the other end of the spectrum, we can use the genomic abundance of transcription factor proteins to estimate upper bounds on *w*/*c* values for adult nematode and mouse cells, yielding estimates of *w*/*c* ≤ 698 and *w*/*c*≤ 1,426, respectively (43). In this case, Eq. **B.3** predicts that cognate binding accounts for only a small fraction of total binding interactions—as little as 1/8 in worms and 1/15 in mice—suggesting that equilibrium affinity differences alone may be insufficient in these cases.

**Fig. 4.**
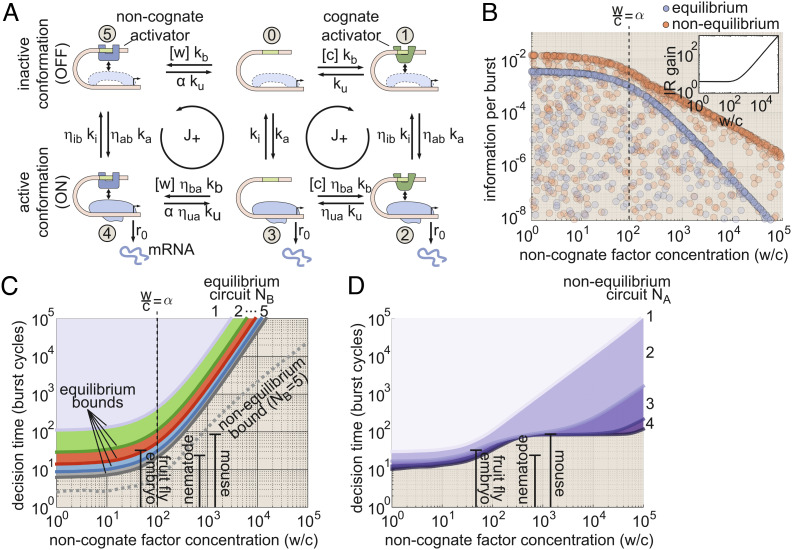
Energy dissipation is key to driving cellular decisions in the presence of noncognate factor interference. (*A*) Incorporating noncognate activator binding leads to a six-state model that features both a right and a wrong activation pathway. (*B*) Numerical results for the maximum achievable information rate for equilibrium (blue circles) and nonequilibrium (red circles) gene circuits with one activator binding site and one activation step (illustrated in *A*) as a function of the relative concentration of noncognate activators *w*/*c*. The inset panel shows the nonequilibrium performance gain (the upper bound of red divided by the upper bound of blue) as a function of *w*/*c*. (*C*) Shaded regions indicate parameter sweep results for the range of achievable decision times for equilibrium gene circuits with 1 to 5 activator binding sites as a function of *w*/*c*. The dashed gray line indicates the lower bound for decision times driven by nonequilibrium gene circuits with five binding sites and one activation step. *SI Appendix*, Fig. S3*B* for corresponding information rate ranges. (*D*) Decision times for nonequilibrium gene circuits with 1 to 4 activation steps. *SI Appendix*, Fig. S3*C* for corresponding information rate ranges. (All decision time quantities assume *ε* = 0.32.)

To examine how interference impacts the timescale of biological decisions, we must extend our gene circuit model to incorporate interference from noncognate activator binding. Drawing inspiration from ref. 41, we add a second “wrong” activation cycle to our original four-state model (Fig. 1*C*), wherein the binding of a noncognate factor to the gene locus can also induce transitions to the active conformation. This leads to the six-state model shown in Fig. 4*A*, where, for simplicity, we have grouped all noncognate activators into a single concentration term: *W*. Here, states 5 and 4 are identical to states 1 and 2, except that a noncognate activator species (blue circle) is bound rather than the cognate activator (green square). For notational convenience, we write the unbinding rates of the noncognate activator *k*_u_^*w*^ as the unbinding rate of the cognate factor *k*_u_ multiplied by an affinity factor *α* = *k*_u_^*w*^/*k*_u_, with *α* = 100.

We employed parameter sweeps to examine the upper limits on information transmission as a function of the ratio of wrong-to-right activator concentrations (*w*/*c*). We held the cognate factor concentration at *C* = c^*^, such that *W* was the only variable concentration parameter. Fig. 4*B* presents the range of achievable information rates as a function of the relative wrong factor concentration. Our results reveal that the rate of information transmission at equilibrium drops precipitously once *w*/*c* exceeds *α* (blue circles in Fig. 4*B*). Away from equilibrium, the upper information limit likewise decreases with *w*/*c*; however, we find that nonequilibrium gene circuits are significantly more robust to high noncognate factor concentrations than equilibrium systems. The relative IR gain from energy dissipation with respect to equilibrium increases from a factor of 4 when *w*/*c* ≈ 1 to a factor of 1,000 when *w*/*c* ≈ 10^5^ (Fig. 4*B*, *Inset*). This shift in information gain suggests that a qualitative change occurs in how energy is used once *w*/*c* > *α* (vertical dashed line in Fig. 4*B* ).

We next used Eq. **3** to calculate the amount of time required for a cell to decide between concentrations *c*_0_ and *c*_1_ of the cognate activator species for different values of *w*/*c*, starting with gene circuits constrained to operate at equilibrium. As in Fig. 2*B*, we compared our model’s performance to the decision time limits for different biological systems, this time with each organism placed appropriately along the *w*/*c* axis. In all organisms considered, gene circuits generally have a few tens of burst cycles over which to transmit information, with no organism exceeding 100 bursts (black error bars in Fig. 4*C*). This decision time limit is significantly faster than can be achieved by our six-state model with one binding site and one activation step at equilibrium, even with negligible amounts of noncognate transcription factor (*w*/*c* = 1, purple shaded region corresponding to N_B_ = 1 in Fig. 4*C*).

Next, we investigated the effect of having equilibrium gene circuits with multiple sites. Fig. 4*C* indicates that equilibrium gene circuits with three or more activator binding sites (red, blue, and gray regions) are sufficient to drive timely decisions in “low-interference” systems such as the early fruit fly embryo. However, we again observe a precipitous decline in performance once *w*/*c* > *α*. Indeed, the best equilibrium model (N_B_ = 5) can drive decisions in no fewer than 1,100 burst cycles—the equivalent of at least 550 h (3 wk) for mouse cells—when *w*/*c* ≈ 1, 400 (the upper limit for mice). This finding is over an order of magnitude too slow for the mouse system’s decision time limit of 86 burst cycles (Fig. 4*C*). Moreover, our analysis suggests that at least 17 activator binding sites are needed to reach this limit at equilibrium (*SI Appendix*, Fig. S3*A*). Such a number is conceivable for eukaryotic enhancers, but this analysis emphasizes that equilibrium systems—even those with biologically salient numbers of binding sites—struggle to achieve realistic decision times in the presence of significant noncognate factor interference.

How do nonequilibrium gene circuits fare? The dashed gray line in Fig. 4*C* indicates the lower decision time limit for nonequilibrium gene circuits with five binding sites and one activation step. We observe a substantial improvement relative to the equilibrium case; however, the performance nonetheless suffers at large values of *w*/*c*, falling short of the decision time limit for the mouse system (209 vs. 86 burst cycles).

We used our parameter sweep algorithm to examine the impact of increasing the number of molecular activation steps (N_A_ > 1) in nonequilibrium gene circuits with a single activator binding site. This revealed substantial improvements, particularly at large *w*/*c* values. The nonequilibrium N_A_ = 1 system required at least 1,500 burst cycles when *w*/*c*= 1,400, whereas gene circuits with two activation steps can drive decisions between *c*_0_ and *c*_1_ in as little as 104 bursts (Fig. 4*D*). Adding a third step further improves this bound to 83 burst cycles, below the 86-burst limit for the mouse system. Moreover, this N_A_ = 3 system exhibits remarkable robustness to noncognate factor interference, sustaining the same level of performance up to *w*/*c* ≈ 10^4^ (Fig. 4*D*).

These results suggest that, in biological contexts where the ratio of wrong-to-right activator concentrations exceeds the intrinsic binding affinity difference (*α*), energy dissipation increasingly becomes a necessary precondition for driving cellular decisions within biologically salient timescales.

### F. Noncognate Factor Concentration Defines Performance Tradeoffs Between Sharpness and Specificity.

**Fig. 5.**
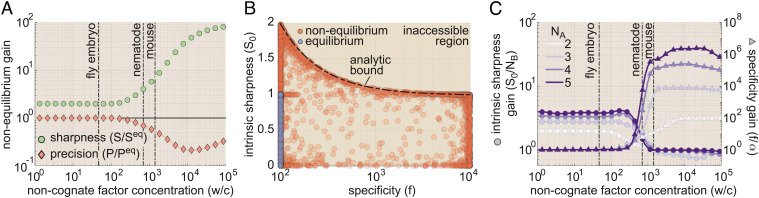
A shifting optimality landscape for information transmission. (*A*) Nonequilibrium gains in sharpness and precision as a function of w/c for six-state (N_B_ = 1, N_A_ = 1; Fig. 4*A*) gene circuits found to drive maximum information rates. IR−maximizing gene circuits are drawn from optimal systems uncovered in the parameter sweeps from Fig. 4*D*. Values above 1 indicate that the system is dissipating energy to enhance performance. The black line indicates a “break-even” point where the nonequilibrium value is equal to the equilibrium maximum. *SI Appendix*, Fig. S4*A* for results for systems with N_A_ > 2. (*B*) Tradeoffs between intrinsic sharpness (S_0_) and specificity (*f*) for equilibrium and nonequilibrium networks. Note that equilibrium gene circuits have no horizontal dispersion because all are constrained to have *f*^*eq*^ = *α*. The black dashed line indicates the bound predicted by *SI Appendix*, Eq. S101. (*C*) Nonequilibrium gains in intrinsic sharpness and specificity for IR-maximizing gene circuits as a function of *w*/*c*. Values above 1 indicate that the system is dissipating energy to enhance sharpness or specificity. Note that the left and right axes have different scales.

Next, we investigated how much sharpness and precision each contribute to the IR gain depicted in Fig. 4*B*, *Inset*. Fig. 5*A* shows the relative nonequilibrium gains in S and P (S/S^eq^ and P/P^eq^) as a function of *w*/*c* for information-maximizing realizations of the six-state gene circuit model shown in Fig. 4*A*. The plot reveals that IR-maximizing gene circuits consistently utilize energy to drive sharpness above its equilibrium limit (S/S^eq^ > 1), while precision is maintained at or below its equilibrium limit (P/P^eq^ ≲ 1). Moreover, the degree to which nonequilibrium gene circuits amplify S increases dramatically as *w*/*c* increases, from a factor of 2 when *w*/*c* ≈ 1 to a factor of 100 when *w*/*c* ≈ 10^4^ (Fig. 5*A*). Thus, the key to understanding how energy increases IR at large *w*/*c* values lies in understanding transcriptional sharpness.

We find that the upper nonequilibrium S limit is given by

[4] $$\begin{array}{c} \;\text{S}\;\leq \frac{f}{\frac{w}{c}+f}\;\;\times \underset{\begin{array}{c} \;\text{intrinsic}\\ \;\text{sharpness} \end{array}}{\underbrace{\;{\text{S}}_{0}}}, \end{array}$$

where the transcriptional specificity, *f*, is defined as the ratio of the probability of having cognate versus noncognate factors bound at the locus (Box 1), and where the intrinsic sharpness (S_0_) is a gene circuit’s normalized sharpness absent noncognate factor binding (i.e., *w* = 0; *SI Appendix*, Appendix Q).

To probe the interplay between intrinsic sharpness and specificity, we employed parameter sweeps for the six-state system in Fig. 4*A*. At equilibrium, this analysis indicated that intrinsic sharpness is constrained such that S_0_ ≤ 1 (consistent with Fig. 3*A*) and that specificity is fixed at *α* (Fig. 5*B*). Indeed, we find that *f*^*eq*^ = *α* for all models considered that operate at equilibrium (*SI Appendix*, Appendix P.3), irrespective of the number of binding sites or activation steps, placing strict limits on information transmission at equilibrium when *w*/*c* is large.

Away from equilibrium, systems can overcome these constraints, achieving up to a two-fold increase in S_0_ and increasing specificity by up to an additional factor of *α* (100) to reach an upper limit of *α*^2^ (Fig. 5*B*). The observed 100-fold increase in *f* is comparable to the gain in the observed sharpness (S) in Fig. 5*A*, suggesting that the S gain at high *w*/*c* arises from nonequilibrium increases in specificity, rather than in intrinsic sharpness. Why not spend energy to simultaneously increase *S*_0_ by two-fold and *f* by 100-fold to achieve S/S^*eq*^ = 2 × *α* = 200? Our analysis reveals a steep tradeoff between specificity and intrinsic sharpness away from equilibrium, with the maximum value of S_0_ = 2 realizable only when specificity is at its equilibrium level (*f* = *α*) and vice versa (Fig. 5*B* and *SI Appendix*, Appendix Q for further details). We find similar nonequilibrium tradeoffs between *f* and S_0_ for more complex molecular architectures (*SI Appendix*, Fig. S4*A*).

The inexorable tradeoff between the intrinsic sharpness S_0_ and specificity *f* illustrated in Fig. 5*B* means that gene loci must “choose” between allocating energy to maximize intrinsic sharpness and allocating energy to maximize specificity. To examine how the concentration of noncognate factors shapes this tradeoff, we took IR-maximizing nonequilibrium gene circuits spanning the relevant range of *w*/*c* values for systems with 1 to 4 activation steps and calculated S_0_ and *f*. Fig. 5*C* illustrates the relative nonequilibrium gains in intrinsic sharpness and specificity for these circuits as a function of *w*/*c*.

Fig. 5*C* reveals that the relative noncognate factor concentration, *w*/*c*, defines a shifting optimality landscape. At low noncognate factor concentrations, maximally informative gene circuits spend energy exclusively to maximize intrinsic sharpness (S_0_/N_B_ > 1 for all systems on the left-hand side of Fig. 5*C*) at the cost of equilibrium specificity levels (*f*/*α* = 1). However, once *w*/*c* surpasses the affinity factor *α*, IR maximization starts to disfavor sharpness (see decreasing S_0_ near *w*/*c* = 10^2^ in Fig. 5*C*) and increasingly depends on enhancing specificity to nonequilibrium levels. We also find that the presence of multiple activation steps dramatically increases the upper nonequilibrium specificity limit, such that *f*^*neq*^ ≤ *α*^N_A_ + 1^, (*SI Appendix*, Fig. S4*B*). Together, these results indicate that the optimal molecular strategy for transmitting information changes according to a scale set by the relative amount of noncognate factor interference, *w*/*c*, and the kinetic binding differences between cognate and noncognate factors, *α*.

### G. Predicting Experimental Signatures of Nonequilibrium Processes in Transcriptional Regulation.

In this section, we examine how simple experiments can identify signatures of nonequilibrium performance in real biological systems. For simplicity, we focused on the gene circuit in Fig. 4*A* with one binding site and one molecular activation step.

**Fig. 6.**
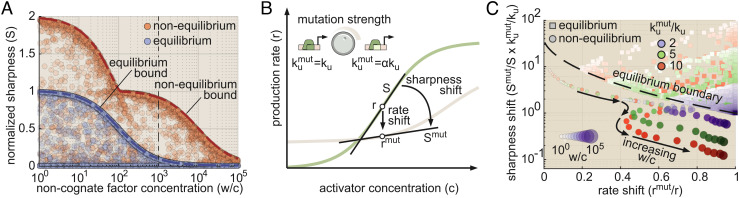
Experimental signatures of nonequilibrium processes in transcriptional regulation. (*A*) Observed sharpness as a function of *w*/*c* for equilibrium (blue circles) and nonequilibrium (red) gene circuits. Dashed blue and red lines indicate analytic sharpness bounds predicted by *SI Appendix*, Eq. S101 in Appendix Q. The black dashed line indicates the point where *w*/*c* = 10^3^. (*B*) Illustration of proposed binding site perturbation experiments. Reducing site specificity is predicted to reduce both the observed sharpness, S, and the mRNA production rate, $\bar{r}$. The strongest possible perturbation would entail a conversion from cognate specificity (*k*_u_) to noncognate specificity (*αk*_u_). (*C*) Phase-space plot of predicted sharpness shift versus rate shift for equilibrium (squares) and nonequilibrium (circles) gene circuits at three binding site perturbation strengths. Note that we normalize the sharpness fold change by *k*_u_/*k*_u_^mut^, which allows us to plot results for different mutation strengths on the same y-axis. Shading indicates the *w*/*c* value (darker shades correspond to higher values). Additionally, the circle size indicates the *w*/*c* magnitude for nonequilibrium circuits. We see that, regardless of noncognate concentration and perturbation strength, nonequilibrium systems do not cross the equilibrium boundary (dashed line). Results assume the initial transcription rate of the wild-type gene is at half-maximum ($\bar{r}=0.5r_{0}$).

Recent works have shown that strict equilibrium limits on transcriptional sharpness can be calculated if the number of activator binding sites is known, suggesting that sharpness might serve as an accessible signature of nonequilibrium regulatory mechanisms (11, 17). However, these studies did not consider off-target activation from noncognate activator species. What happens when we account for noncognate factor binding? As illustrated in Fig. 6*A*, numerical parameter sweeps of S vs. *w*/*c* indicate that the upper S limit decreases dramatically with increasing *w*/*c* for both equilibrium (blue circles) and nonequilibrium gene (red circles). Thus, the upper sharpness limit is not absolute but instead depends on the concentration of noncognate factors in the cell. This *w* dependence must be considered to accurately interpret experimental measurements.

For instance, consider the case where *w*/*c* = 10^3^ (black dashed vertical line in Fig. 6*A*), a plausible value for mammalian systems (41, 43, 44). Our model predicts that the maximum achievable S for nonequilibrium gene circuits is 0.91. This far exceeds the true equilibrium sharpness limit of 0.09 when noncognate interference is accounted for (blue dashed line in Fig. 6*A*). However, S = 0.91 falls below the “naive” equilibrium bound of S = 1 that one would predict if *w* were not accounted for (see blue bound on far-left-hand side of Fig. 6*A* and *SI Appendix*, Fig. S5*A*). Thus, failing to account for noncognate factor interference could mask strong nonequilibrium signatures. However, accurately measuring *w*/*c* may be challenging in many settings since *w* comprises the aggregate activity of all noncognate activator species.

In light of this challenge, we propose a complementary experimental approach that is more robust to uncertainty regarding the precise value of *w*/*c*. As illustrated in Fig. 6*B*, this method involves measuring changes in gene expression at *C* = c^*^ that result from point mutations to the activator binding site, which thereby leads to a higher unbinding rate, *k*_u_^mut^, for cognate activators (*k*_u_^mut^/*k*_u_ > 1). Although *w*/*c* may be difficult to estimate in many biological contexts, robust algorithms can predict changes in binding energies from the DNA sequence of transcription factor binding sites (45). We employ two metrics to quantify the resulting change in gene expression: fold changes in the mRNA production rate (${\bar{r}}^{\mathrm{mut}}/\bar{r}$) and in the normalized sharpness (S^mut^/S), each defined as the quantity corresponding to the mutated binding site divided by the wild-type value (Fig. 6*B*).

To illustrate the method, we used our model to predict outcomes for the case where the wild-type gene circuit is expressing at half its maximum rate ($\bar{r}=0.5r_{0}$). Overall, we find that IR-optimized nonequilibrium gene circuits are highly sensitive to changes in cognate activator specificity and that this sensitivity can be used to probe for nonequilibrium behavior. At low *w*/*c* levels (*w*/*c* ≲ 10^3^), mutated nonequilibrium circuits exhibit larger shifts in their transcription rate than can be achieved at equilibrium (*SI Appendix*, Fig. S5*B*). Meanwhile, when *w*/*c* > 10^3^, IR-optimized nonequilibrium systems experience a substantially larger sharpness decrease than even maximally sharp equilibrium circuits (*SI Appendix*, Fig. S5*C*). Consequently, when combined, S^mut^/S and ${\bar{r}}^{\mathrm{mut}}/\bar{r}$ define a perturbation response space in which nonequilibrium gene circuits that transmit information at optimal (or near-optimal) levels are completely disjoint from equilibrium systems. This is illustrated in Fig. 6*C*, which compares our model’s predictions for S^mut^/S vs. ${\bar{r}}^{\mathrm{mut}}/\bar{r}$ for maximally informative nonequilibrium gene circuits to the full range of achievable values for equilibrium gene circuits (circles and squares, respectively). Despite the fact that we examine three binding site perturbation strengths and a wide range of noncognate factor concentrations, we find that optimal nonequilibrium systems never cross the equilibrium boundary (dashed line). Thus, by measuring S^mut^/S and ${\bar{r}}^{\mathrm{mut}}/\bar{r}$, we can obtain clear-cut signatures on nonequilibrium regulation, even when *w*/*c* is unknown.

## 2. Discussion

In this work, we employed simple kinetic models of transcription to investigate how energy dissipation within the transcriptional cycle impacts the rate at which a gene circuit drives cellular decisions. We found that biologically plausible rates of energy dissipation can drive significant gains in the information transmission rate and discovered that the regulatory mechanisms underlying these nonequilibrium gains change from increased sharpness to increased specificity depending on the level of interference from noncognate factor binding.

### Performance Tradeoffs Dictate Limits of Information Transmission Away from Equilibrium.

We have established that although energy dissipation can increase transcriptional sharpness, precision, and specificity individually, these gains cannot be realized simultaneously. For negligible noncognate factor binding, we showed that IR is dictated by a tradeoff between sharpness (S) and precision (P). For all models considered, we discovered that the information rate was maximized by systems that boosted transcriptional sharpness (not precision) above its equilibrium limit (Fig. 3*A* and *SI Appendix*, Fig. S2 *A* and *B*).

Similarly, our analysis revealed that nonequilibrium gains in specificity and sharpness cannot occur simultaneously (Fig. 5*B* and *SI Appendix*, Fig. S4*B*). This incompatibility arises from the fact that intrinsically sharp systems are tuned to amplify concentration-dependent activator binding rates, whereas specific systems amplify differences in unbinding rates between cognate and noncognate activator species. Our model predicts that *w*/*c* defines a shifting optimality landscape, wherein nonequilibrium gene circuits that maximize intrinsic sharpness drive the fastest decisions when *w*/*c* ≤ *α*, but the optimal strategy begins to shift from increasing sharpness to activator proofreading when *w*/*c* > *α* (Fig. 5*C*). A recent study reported the potential for this kind of context-dependent shift from sharp to specific gene circuits (18), although sharpness was only investigated at its equilibrium limit. Here, we provide quantitative predictions for how gene circuits navigate this sharpness-specificity tradeoff far from equilibrium.

### Activation Steps Amplify Nonequilibrium Performance Gains.

Another key finding of this work is that the presence of multiple activation steps, wherein multiple molecular components must engage to achieve transcription, can amplify nonequilibrium gains in transcriptional sharpness (Fig. 3*B*). Our result is evocative of a recent study (46) demonstrating that equilibrium systems with multiple conformational degrees of freedom can achieve sharper, more flexible transcriptional input–output functions. Notably, however, the systems in this work still adhered to the fundamental equilibrium limitation that sharpness cannot exceed the number of activator binding sites (S ≤ N_B_). Thus, our findings further emphasize potential benefits of the conformational complexity of the eukaryotic gene cycle.

Consistent with previous results in the kinetic proofreading literature (47), we also found that gene circuits with multiple activation steps can realize dramatic increases in transcriptional specificity out of equilibrium, such that the specificity (*f*) scales exponentially with N_A_ (*f* ≤ *α*^N_A_ + 1^; *SI Appendix*, Fig. S4*B*). This result extends the findings of a recent work examining specificity in systems with up to two activation steps (14). Yet there exists an important asymmetry between sharpness and specificity: Although the addition of activator binding sites can increase the sharpness S at equilibrium, energy dissipation constitutes the only route (short of altering activator binding sequences) for increasing specificity *f* above the intrinsic affinity factor *α*. Thus, for large *w*/*c*, energy dissipation overcomes a fundamental limitation of eukaryotic gene circuits—the lack of binding specificity—that no equilibrium mechanism can address.

### Equilibrium Regulatory Schemes May Be Sufficient in Many Real Biological Systems.

While activator proofreading may be critical when *w*/*c* is large, our analysis suggests that it is unlikely to constitute a universal constraint on gene regulatory architectures. Indeed, even relatively simple equilibrium architectures with 3 to 5 binding sites should suffice to drive timely cellular decisions in “low-interference” systems such as the fruit fly embryo (Fig. 4*C*). Moreover, while simple estimates based on genomic transcription factor abundances suggest that many eukaryotic systems may exceed the *w*/*c* = *α* interference limit, these estimates likely represent upper bounds on *w*/*c*, since different cell types selectively express distinct subsets of transcription factors (48). In addition, we note that the relative size of the concentration difference between *c*_1_ and *c*_0_ (*δc*/*c*)—which we assumed to be 0.1—plays a key role in dictating the information transmission rate, Eq. **2** and will vary across different biological contexts.

### Different Frameworks for Examining the Impact of Noncognate Factor Binding.

In considering the impact of noncognate factor binding, we drew inspiration from a previous study examining competition between cognate and noncognate transcription factors to bind and activate a single gene locus (41). This formulation of the problem is distinct from the approach taken in two recent works, which addressed the problem of specificity from the perspective of a single activator species that interacts with two different gene loci: a cognate (with specific binding sites) and a noncognate locus (without specific binding sites) (14, 18). While both approaches have proven fruitful, we favor the “single-locus” approach, since it captures the effects of competitive binding between different species, which is an unavoidable reality of crowded cellular environments.

Moreover, this shift in perspectives has meaningful consequences. A previous study found that the equilibrium limit of *f* = *α* could only be achieved at the cost of high levels of transcriptional noise (14). Yet, we find that this tradeoff evaporates once competition between cognate and noncognate factors is considered since *f* becomes fixed at *α* in this case (Fig. 5*B*). Additionally, previous studies have reported transcriptional sharpness as a potential indicator of nonequilibrium optimization (11, 17). Our analysis reaffirms this idea but, crucially, reveals that one must consider the relative concentration of noncognate factors (*w*/*c*) to accurately assess whether a system is performing above the equilibrium limit since this limit decreases as *w*/*c* increases (Fig. 6*A* and *B*).

### Future directions.

While we have considered gene loci with varying numbers of specific activator binding sites, real enhancers also contain significant stretches of “neutral” DNA with no binding sites, as well as weak activator sites that fall below typical thresholds used to identify specific sites (22, 49). This focus on specific sites is widespread in theoretical studies of transcription (3, 11, 17, 41), despite the well-established importance of weak binding sites in the context of certain genes (49–51). We propose that the kinetic models utilized herein could readily be extended to feature some combination of specific and neutral sites. More ambitiously, the field would benefit from the introduction of nonequilibrium models that account for the reality that transcription factors interact with a continuum of sites along enhancer DNA.

On the experimental side, we advocate for the expanded use of theoretically tractable synthetic enhancer systems in which the number and identity of binding sites can be well established. Several recent studies constitute promising steps in this direction (11, 22, 52, 53). Additionally, synthetic transcription factor systems that can act orthogonally to endogenous regulatory networks represent an intriguing platform for investigating transcriptional specificity (54). Lastly, statistical methods that infer how transcription factors impact the kinetics of bursting (30, 31, 55) hold promise for connecting macroscopic experimental measurements to microscopic theoretical models of transcription. Looking ahead, holistic research that integrates cutting-edge experiments, statistical methods, and theory will be key to bridging the as yet yawning gap between enhancer sequence and gene regulatory function.

## Methods

Gene circuits were modeled as stationary Markov processes (*SI Appendix*). Calculations where performed using Mathematica and Matlab.

## Supplementary Material

Appendix 01 (PDF)

## Data Availability

Codebase containing scripts for calculations and simulation results used in the main text and appendices, data have been deposited in https://github.com/nlammers371/noneq-gene-regulation.git (NA).
