# Supplementary material for: Competing constraints shape the nonequilibrium limits of cellular decision-making
Source: Proc Natl Acad Sci U S A. 2023 Mar 2;120(10):e2211203120. doi: 10.1073/pnas.2211203120 (PMC10013869; doi:10.1073/pnas.2211203120)
Supplement: Supplementary file 1 — Appendix 01 (PDF) [file pnas.2211203120.sapp.pdf]

Supporting Information for

Competing constraints shape the non-equilibrium limits of cellular decision making

Nicholas C. Lammers, Avi I. Flamholz and Hernan G. Garcia

Hernan G. Garcia
E-mail: hggarcia@berkeley.edu

This PDF file includes:

Supporting text
Figs. S1 to S20
SI References

Supplementary Figures

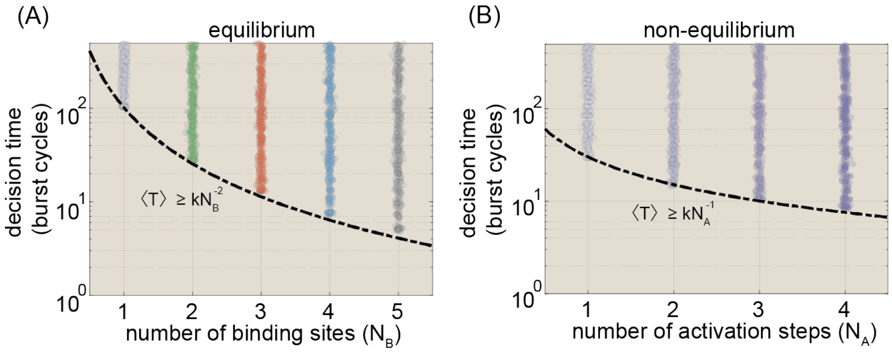

Fig. S1. **Decision times for different gene circuit architectures.** **(A)** Parameter sweep results for equilibrium gene circuits with different numbers of activator binding sites. Black dashed line indicates lower limit of the decision time and is a function of the form $\langle \mathrm{T} \rangle = k\mathrm{N_B}^{-2}$, where k is a proportionality constant. **(B)** Plot of range of achievable decision times for non-equilibrium gene circuits with a single activator binding site ($\mathrm{N_B} = 1$) as a function of the number of activation steps, $\mathrm{N_A}$. The dashed line indicates the lower decision time bound, and is a function of the form $\langle \mathrm{T} \rangle = k\mathrm{N_A}^{-1}$. (All results shown assume an error probability of 32%. For parameter sweep results in A and B, transition rate and interaction term magnitudes, k and η, were constrained such that $10^{-5} \leq k\tau_b \leq 10^5$ and $10^{-5} \leq \eta \leq 10^5$, where τ_b is the burst cycle time. η_{ab} and η_{ib} were further constrained such that $\eta_{ab} \geq 1$ and $\eta_{ib} \leq 1$, consistent with our assumption that the transcription factor activates the gene locus.)

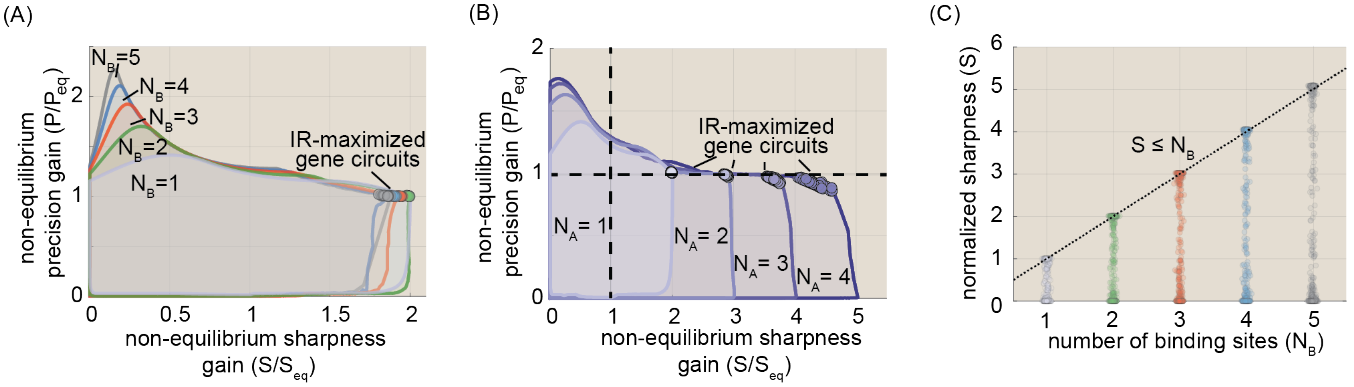

Fig. S2. Tradeoffs between sharpness and precision persist for more complex gene regulatory architectures. **(A)** Non-equilibrium gains in sharpness and precision for gene circuits with different numbers of activator binding sites (N_B) and one activation step. Shaded regions indicate achievable regimes for each system, as determined by no fewer than 10,000 unique simulated gene circuits. Circles indicate location of information-maximizing gene circuits for each mode (top 99.9th percentile). **(B)** Non-equilibrium gains in sharpness and precision for gene circuits with different numbers of activation steps (N_A) and one activator binding site. Dashed lines indicate Hopfield barriers that dictate the limits of equilibrium performance. These limits are the same, irrespective of N_A. **(C)** Scatter plot indicating sharpness levels for equilibrium gene circuits as a function of the number of binding sites. Bounding line is for a function of the form $S = N_B$. (For parameter sweep results in A-C, transition rate and interaction term magnitudes, k and η, were constrained such that $10^{-5} \leq k\tau_b \leq 10^5$ and $10^{-5} \leq \eta \leq 10^5$, where τ_b is the burst cycle time. η_{ab} and η_{ib} were further constrained such that $\eta_{ab} \geq 1$ and $\eta_{ib} \leq 1$, consistent with our assumption that the transcription factor activates the gene locus.)

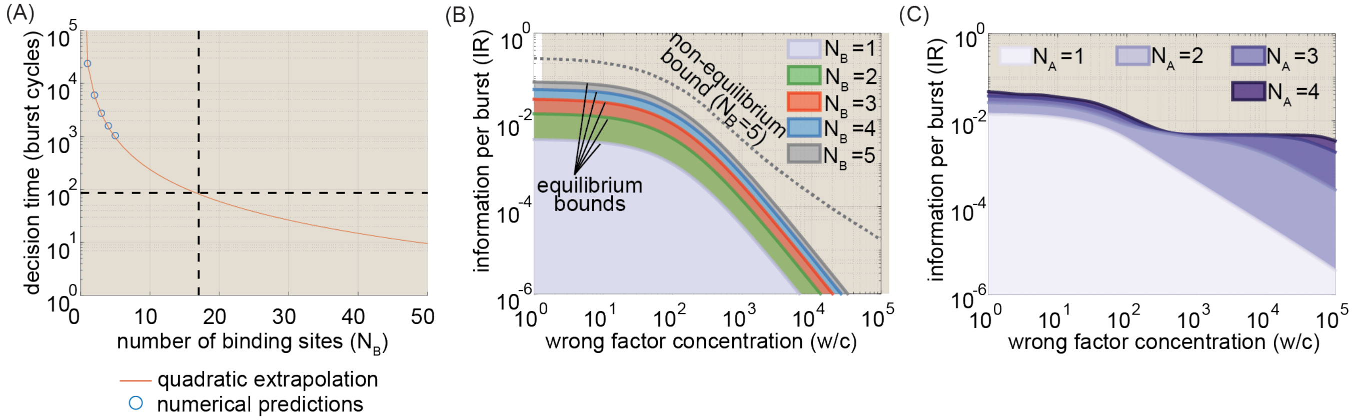

Fig. S3. Supplemental analyses for the dependence of IR with non-cognate transcription factor interference. (A) Extrapolation of minimum decision times for equilibrium gene circuits as a function of number of activator binding sites based on numerical results for circuits with 1-5 binding sites. Analysis indicates that at least 17 sites would be required to achieve plausible decision ties in the context of the mouse system. **(B)** Parameter sweep results showing the range of achievable information rates as a function of w/c for equilibrium gene circuits with 1-5 activator binding sites and one molecular activation step. **(C)** Sweep results for non-equilibrium gene circuits with 1-4 activation steps and a single activator binding site. (For parameter sweep results in B and C, transition rate and interaction term magnitudes, k and η, were constrained such that $10^{-5} \leq k\tau_b \leq 10^5$ and $10^{-5} \leq \eta \leq 10^5$, where τ_b is the burst cycle time. η_{ab} and η_{ib} were further constrained such that $\eta_{ab} \geq 1$ and $\eta_{ib} \leq 1$, consistent with our assumption that the transcription factor activates the gene locus.)

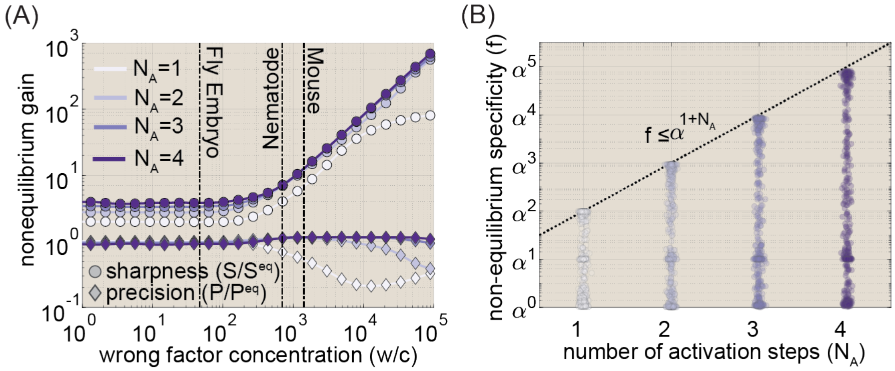

Fig. S4. **Supplemental results for main text Figure 5.** **(A)** Non-equilibrium sharpness and precision gains for IR-maximizing gene circuits with 1-4 activation steps. **(B)** Range of achievable specificity values for non-equilibrium gene circuits with with 1-4 activation steps. (Transition rate and interaction term magnitudes, k and η, were constrained such that $10^{-5} \leq k\tau_b \leq 10^5$ and $10^{-5} \leq \eta \leq 10^5$, where τ_b is the burst cycle time. η_{ab} and η_{ib} were further constrained such that $\eta_{ab} \geq 1$ and $\eta_{ib} \leq 1$, consistent with our assumption that the transcription factor activates the gene locus.)

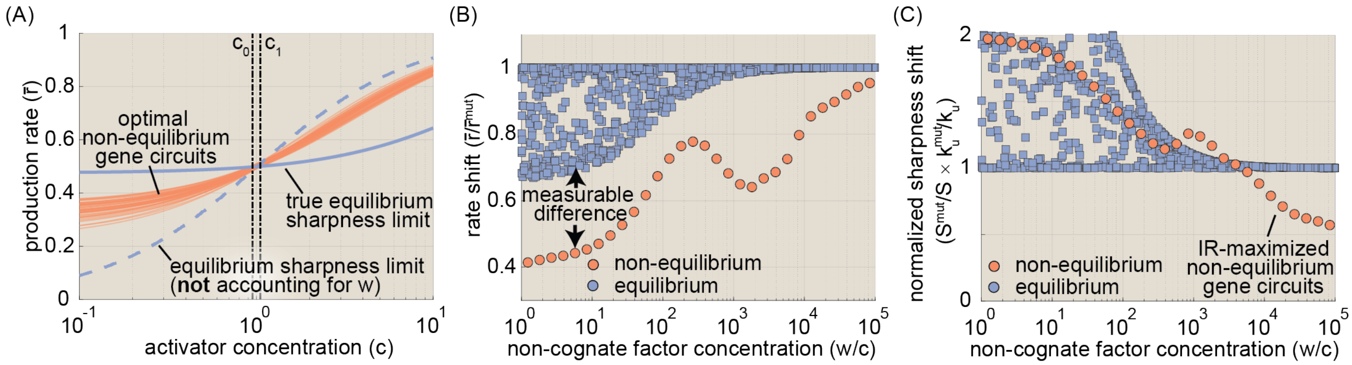

Fig. S5. Experimental signature of energy expenditure. **(A)** Predicted induction curves for 50 near-optimal non-equilibrium gene circuits when $w = 10^3 c^*$ (red lines), as well as the *actual* induction curves for the sharpest achievable equilibrium curve (solid blue line) and the (incorrect) limit when that would be predicted if w was not accounted for (dashed line). Note that the sharpness of the red curves falls above the true equilibrium limit but below the naive limit. **(B)** Predicted shift in the production rate resulting from a binding site perturbation that doubles the unbinding rate ($k_{\mathrm{u}}^{\mathrm{mut}}/k_{\mathrm{u}} = 2$)—equivalent to a energetic difference of 0.7 k$_B$T—for equilibrium gene circuits (blue squares) and IR-maximizing non-equilibrium circuits (red circles). Note that non-equilibrium circuits are far more sensitive than equilibrium circuits when $w/c < 10^4$. **(C)** Predicted sharpness shift upon perturbing the activator binding site. The non-equilibrium shift becomes markedly larger than equilibrium limit when $w/c > 10^3$. (For parameter sweep results in B and C, transition rate and interaction term magnitudes, k and η, were constrained such that $10^{-5} \leq k\tau_b \leq 10^5$ and $10^{-5} \leq \eta \leq 10^5$, where τ_b is the burst cycle time. η_{ab} and η_{ib} were further constrained such that $\eta_{ab} \geq 1$ and $\eta_{ib} \leq 1$, consistent with our assumption that the transcription factor activates the gene locus.)

Supporting Information Text

Appendices

A. Analytic expressions for key gene circuit characteristics. This section lays out analytic expressions for key quantities that play a central role in the investigations undertaken over the course of the main text. We do not repeat derivations for expressions that are treated separately elsewhere in these Appendices, and avoid re-deriving expressions from scratch, unless they are novel to this work.

A.1. The transition rate matrix and activity vector. Consider a gene circuit g that has N different microscopic states. We assume that microscopic transitions between the molecular states that make up g are Markovian, such that our system can be modeled as a continuous time Markov chain (CTMC). It follows that the steady-state behavior of g is fully determined by two quantities: the transition rate matrix, $\boldsymbol{K}$ and the state activity vector, $\boldsymbol{a}$.

$\boldsymbol{K}$ is a $N \times N$ matrix with off-diagonal elements that encode the rates with which the system switches between microscopic rates. For instance, k_{mn}—the element in the mth row and nth column of $\boldsymbol{K}$—gives the transition rate going from state n to state m. The diagonal elements of $\boldsymbol{K}$ are negative, and are scaled such that each column of $\boldsymbol{K}$ sums to 0. The activity vector $\boldsymbol{a}$ is a binary vector of length N that contains a "1" for each state that is transcriptionally active, and a "0" for inactive states. We assume that both $\boldsymbol{K}$ and $\boldsymbol{a}$ are fixed in time.

A.2. A generic expression for the rate of energy dissipation. Equation 1 gives an expression for the rate of energy dissipation (also termed entropy production), Φ, in the context of the four-state model shown in Figure 1C. This is a special case of a more general formula for Φ that applies to arbitrary molecular architectures. From (1, 2), we have

$$\Phi = \sum_{i=i}^{K} \sum_{j \neq i}^{K} \pi_i k_{ji} \ln\left(\frac{k_{ji}}{k_{ij}}\right). \quad \text{[S1]}$$

We use Equation S1 to calculate all energy dissipation rates given throughout the main text. In the case of the simple four-state system shown in Figure 1B, we have from (1) that Equation S1 simplifies to

$$\Phi = \boldsymbol{J} \ln \frac{k_{\mathrm{b}} \eta_{ab} k_{\mathrm{a}} \eta_{ua} k_{\mathrm{u}} k_{\mathrm{i}}}{k_{\mathrm{b}} k_{\mathrm{a}} \eta_{ba} k_{\mathrm{u}} \eta_{ib} k_{\mathrm{i}}}, \quad \text{[S2]}$$

which further simplifies to

$$\Phi = \boldsymbol{J} \ln \frac{\eta_{ab} \eta_{ua}}{\eta_{ba} \eta_{ib}}. \quad \text{[S3]}$$

Here $\boldsymbol{J}$ is the net cycle flux, a quantity with units of inverse time which encodes the rate at which the system completes extra cycles in the clockwise ($\boldsymbol{J} > 0$) or counterclockwise ($\boldsymbol{J} < 0$) directions. Mathematically, $\boldsymbol{J}$ is given by

$$\boldsymbol{J} = \boldsymbol{J}_{+} - \boldsymbol{J}_{-}, \quad \text{[S4]}$$

where $\boldsymbol{J}_{+}$ gives the average rate at which the system completes one full cycle in the clockwise direction (i.e., setting out from state 0 to state 1, reaches state 0 from state 4), and $\boldsymbol{J}_{-}$ is defined analogously. In terms of microscopic quantities, for any system with a single loop we can define $\boldsymbol{J}$ as

$$\boldsymbol{J} = J_{ij} = \pi_i k_{ji} - \pi_j k_{ij}, \quad \text{[S5]}$$

where k_{ji} denotes the transition rate from state i to state j and J_{ij} corresponds to the net transition flux between the two states. We note that the equivalence between $\boldsymbol{J}$ and J_{ij} does not generally hold for more complicated systems that contain multiple cycles. We direct readers to (3) for an excellent introduction to the conceptual machinery needed to analyze cycle fluxes in multi-loop systems.

A.3. State probabilities, transcription rate, and transcriptional noise. A first step to calculating virtually all gene circuit characteristics of interest is to obtain the steady-state vector, $\boldsymbol{\pi}$, which is a vector of length N that gives the steady state probability of finding the gene circuit of any one of the N microscopic states. We can obtain $\boldsymbol{\pi}$ by finding the right eigenvector ($\boldsymbol{v}_R$) of $\boldsymbol{K}$ with an eigenvalue of 0,

$$\boldsymbol{K}\boldsymbol{v}_R = 0, \quad \text{[S6]}$$

and imposing the additional constraint that the elements of $\boldsymbol{\pi}$ sum to 1, such that

$$\boldsymbol{\pi} = \frac{\boldsymbol{v}_R}{\sum_{i=1}^{K} v_i}. \quad \text{[S7]}$$

With this the steady state probability vector in hand, we can calculate the average transcription rate by taking the dot product of $\boldsymbol{a}$ and $\boldsymbol{\pi}$:

$$\bar{r} = r_0 \underbrace{\sum_{i=0}^{K} a_i \pi_i}_{\substack{\text{fraction of} \\ \text{time active } (\pi_{\mathrm{a}})}}, \quad \text{[S8]}$$

where we define the quantity indicated by the underbrace as the average fraction of time, π_{a}, that the system spends in the active state. Throughout the course of this work, we assume that r_0 is held fixed, such that the transcriptional activator may only impact transcription by modulating microscopic transition rates in $\boldsymbol{K}$ to alter $\boldsymbol{\pi}$. Furthermore, since we take Poisson noise from mRNA synthesis to be negligible (see Appendix D), the absolute magnitude of r_0 is unimportant, and we set it to 1 for simplicity. We can obtain the transcriptional sharpness, s, by taking the derivative of Equation S8 with respect to c, such that

$$s = \frac{d\bar{r}}{dc}c, \tag{S9}$$

where we note that we have multiplied by c such that s is in production rate units.

Next, we turn to obtaining an expression for the variance (noise) in gene expression. From Whitt 1992 (4), we have that

$$\sigma^2 = 2\sum_{i=1}^{K}\sum_{j=1}^{K} a_i \pi_i z_{ij} a_j, \tag{S10}$$

where z_{ij} is the element from ith row and jth column of what is known as the fundamental matrix, $\boldsymbol{Z}$ of our transition rate matrix, $\boldsymbol{K}$. $\boldsymbol{Z}$ is a $N \times N$ matrix that plays an integral role in the calculation of many key behaviors of a Markov chain. Once again drawing from Whitt, we can calculate $\boldsymbol{Z}$ using the formula

$$\boldsymbol{Z} = (\boldsymbol{\Pi} - \boldsymbol{K})^{-1} - \boldsymbol{\Pi}, \tag{S11}$$

where $\boldsymbol{\Pi}$ is a $N \times N$ matrix with each row equal to π. We define the transcriptional precision, p, as one over the square root of Equation S10, such that

$$p = \frac{1}{\sigma} \tag{S12}$$

A.4. Using the fundamental matrix to calculate first passage times. First passage times provide a useful conceptual tool for connecting microscopic fluctuations, which often are unobservable, with emergent dynamical behaviors, such as transcriptional bursting. The fundamental matrix provides an invaluable tool for doing this in the context of arbitrarily complex transcriptional systems. Once again, we start with an expression from Whitt 1992 (4) that relates off-diagonal elements of $\boldsymbol{Z}$ to first passage times between microscopic states:

$$z_{ji} = \pi_i[ET_{ei} - ET_{ij}], i \neq j. \tag{S13}$$

Here, ET_{ij} is the mean expected first passage time from state j to state i and ET_{ei} the first passage time to state i at equilibrium, defined as

$$ET_{ei} = \pi_i \sum_{j=1}^{K} \pi_j ET_{ij}. \tag{S14}$$

Now, from (4) we also have that the diagonal elements of $\boldsymbol{Z}$ can be expressed as

$$z_{ii} = \pi_i ET_{ei}. \tag{S15}$$

We can now combine Equations S13 and S15 to solve for the first passage time from state i to state j:

$$ET_{ij} = \frac{z_{ii} - z_{ji}}{\pi_i}. \tag{S16}$$

A.5. Calculating the burst cycle time. First passage times are intimately related to a quantity of central importance throughout the text: the burst cycle time, τ_b, defined as the average time required for a system to complete one ON→OFF→ON cycle (Figure 1D). This is trivial in the case of a simple two state system with a single OFF and ON state and rates k_{on} and k_{off} (Figure S17). In this case, the burst cycle time is simply

$$\tau_b = \frac{k_{\mathrm{on}} + k_{\mathrm{off}}}{k_{\mathrm{on}} k_{\mathrm{off}}}. \tag{S17}$$

The calculation becomes less trivial for systems with larger numbers of states, however. Fortunately, the concepts outlined above provide us with the tools necessary to derive a generic expression for τ_b that applies to systems of arbitrary complexity.

The essence of the procedure lies in calculating effective off and on rates (k^*_{off} and k^*_{on}) from $\boldsymbol{K}$ using first passage times. We go through this procedure in detail for k^*_{on} and note that the same approach applies for k^*_{off}. The activity vector $\boldsymbol{a}$ partitions our system into M OFF states and N ON states. To calculate k^*_{on}, the first step is to estimate the expected amount of time it will take for the system to reach an ON state (any ON state) from each OFF state. We can do this by defining a new transition rate matrix, $\boldsymbol{K}^{\mathrm{OFF}}$, that has dimensions $M + 1 \times M + 1$. The off-diagonal elements of the first M rows an M columns of $\boldsymbol{K}^{\mathrm{OFF}}$ are simply equal to the microscopic rates from $\boldsymbol{K}$ that lead from one of the M OFF states to another OFF state. Together, these molecular states constitute a single coarse-grained OFF state.

The final row and column, however, are different and contain total fluxes into and out of all ON states from each OFF state. An element in the final row of $\boldsymbol{K}^{\text{OFF}}$ is given by

$$q^{\text{OFF}}_{m+1,i} = \sum_{j=1}^{K} a_j k_{ji}, \tag{S18}$$

where a_j is the jth element of the activity vector, k_{ij} is a microscopic rate from the original transition rate matrix, and we assume the state i is in the set of OFF states. Thus, we see that each element of the last row of $\boldsymbol{K}^{\text{OFF}}$ gives the total flux from *all* OFF state into the ON conformation. The elements of the final column have a complementary definition:

$$q^{\text{OFF}}_{i,m+1} = \sum_{j=1}^{K} a_j k_{ij}. \tag{S19}$$

With our condensed transition rate matrix thus defined, we can use Equations S6 and S7 to calculate $\boldsymbol{\pi}^{\text{OFF}}$ and Equation S11 to calculate $\boldsymbol{Z}^{\text{OFF}}$. Then, we can use Equation S16 to obtain a vector $\boldsymbol{et}^{ON}$ of length M, where each element i is defined as the expected first passage time from OFF state i back into *any* of the ON states. Specifically, we have that each element, i, is given by

$$et^{\text{ON}}_i = \frac{z^{\text{OFF}}_{m+1,m+1} - z^{\text{OFF}}_{i,m+1}}{\pi_{m+1}}. \tag{S20}$$

Thus, we have obtained a vector, $\boldsymbol{et}^{\text{ON}}$, of expected mean first passage times out of each OFF state into the set of N active transcriptional states. But how do we weight the different passage times in this vector to arrive at an overall average expectation for the amount of time required for the system to turn back ON following a transition into an OFF state? It's tempting here to use the stead-state probabilities of each OFF state given by $\boldsymbol{\pi}$, but this is actually not correct.

Instead, the key is to recognize that each OFF state should be weighted by the rate at which ON states switch into it. In other words, we weight OFF states by the probability that they are the initial state the system reaches upon switching out of the ON conformation; the gateway into the OFF states. Mathematically, we encode these weights using the flux vector $\boldsymbol{f}^{\text{OFF}}$, which has M elements, each defined as

$$f^{\text{OFF}}_i = \sum_{j=1}^{K} a_j k_{ij} \pi_j, \tag{S21}$$

where a_j is the jth element of the activity vector $\boldsymbol{a}$ (1 for ON states and 0 otherwise), k_{ij} is the transition rate from state j to state i, and π_j is the steady-state probability of state j.

Finally, we combine this expression with Equation S20 to obtain an expression for the average reactivation time as a flux-weighted average of the first passage times out of each OFF state:

$$ET_{\text{OFF}\rightarrow\text{ON}} = \frac{1}{k^*_{\text{on}}} = \frac{\sum_{i=1}^{M} f_i et^{\text{ON}}_i}{\sum_{i=1}^{M} f_i}. \tag{S22}$$

As noted above, the calculations for k^*_{off} follow precisely the same logic, with the roles of the OFF and ON states switched. After this is done, the total burst cycle time, τ_b, is simply

$$\tau_b = ET_{\text{OFF}\rightarrow\text{ON}} + ET_{\text{ON}\rightarrow\text{OFF}}. \tag{S23}$$

Equation S22 is useful because it allows us to relate the (potentially quite complex) microscopic dynamics of a transcriptional system to emergent bursting timescales observed in live imaging experiments (5). To our knowledge, this is the first time that take this flux-weighted first passage time approach is applied to the modeling of burst dynamics. We hope that the expressions provided here will prove useful to others seeking to pursue similar projects in the future.

A.6. Re-scaling transition rates to generate gene circuits with unit burst cycle times. A useful feature implied by Equation S22 and Equation S23 is that the absolute size of τ_b scales inversely with the microscopic rates in the matrix $\boldsymbol{K}$, such that we can decrease τ_b by some scaling factor λ by simply multiplying $\boldsymbol{K}$ by λ. We use this trick to renormalize all time-dependent metrics calculated over the course of our parameter sweeps to have units of burst cycle time. This is done by calculating τ_b for each new model realization we generate, and then multiplying its transition rate matrix by this quantity to generate a normalized rate matrix, namely

$$\boldsymbol{K}^* = \tau_b \boldsymbol{K}. \tag{S24}$$

As a result of this rescaling, the normalized matrix, $\boldsymbol{K}^*$, will have a unit burst cycle time ($\tau_b = 1$). We use this rescaled matrix to calculate all relevant gene circuit characteristics, thus ensuring that all time-dependent performance metrics are given in units of "per τ_b".

B. Gaussian noise approximation. Throughout this work, we make the simplifying assumption that the intrinsic noise in accumulated mRNA levels due to transcriptional bursting is approximately Gaussian. In this section, we use stochastic simulations to put this assumption to the quantitative test. The Markov chain central limit theorem states that the distribution of a quantity that is a function of a Markov chain (such as the transcription rate, $\bar{r}$), will become approximately Gaussian as the number of iterations becomes large (6).

The question, then, is whether can expect the accumulated transcriptional output to approach this limiting Gaussian distribution within timescales that are relevant to the decision times discussed in this work. To determine this, we used stochastic simulations (7) to track the distribution of the accumulated output of 500 random realizations of the four-state system shown in Figure 1C for 5,000 burst cycles. Each realization had a unique set of transition rates and, correspondingly, a unique average rate of transcription, $\bar{r} = \pi_a r_0$, where π_a indicates the fraction of time the system spends in a transcriptionally active molecular state and r_0 is the rate of transcript initiation when active. For each model realization, we ran 100 stochastic simulations. We used these simulations to track the distribution of the apparent average transcription rate for each model realization as function of accumulation time. Figure S6A shows the apparent mean rate across 100 simulations for a single illustrative gene circuit realization. Inset histograms indicate distribution of apparent transcription rates at different time points. As expected, we see that the apparent rates are initially highly dispersed; however, even after 25 burst cycles, we see that $p(\bar{r})$ has become a much narrower, roughly symmetrical distribution that appears approximately Gaussian.

To systematically assess the rate of convergence to normality, we utilized the simple One-sample Kolmogrov-Smirnov test ("kstest", (8)), which tests the null hypothesis that a vector of transcription outputs from realization i at time t, $\boldsymbol{r}_i(t)$, is drawn from a normal distribution. The test returns a p value corresponding to the probability of observing $\boldsymbol{r}_i(t)$ if the transcriptional output were truly Gaussian. In standard implementations $p \lesssim 0.05$ is taken to constitute strong evidence that the output is *not* Gaussian. Thus, to assess convergence to normality, we tracked this p value over time for each of the 500 gene circuit realizations.

Figure S6B shows the average kstest p-values across 10 different sets of gene circuits, grouped by their average rate of transcription. In all cases, we see that noise profiles rapidly converge towards normality, such that all systems cross the (relatively conservative) threshold of $p = 0.1$ within 5 burst cycles (dashed line in Figure S6B). Gene circuits near the tail ends of the induction curve ($\bar{r} \leq 0.1$ and $\bar{r} \geq 0.9$) take the longest to converge, which is likely because it takes longer for distributions near the boundaries to become symmetric about their mean; yet even these converge rapidly.

The fastest decisions discussed in the main text (Figure 4C and D), and most decision times considered are significantly longer than the time for Gaussian convergence revealed by Figure S6B). Thus, we conclude that the Gaussian noise approximation invoked throughout this work is justified.

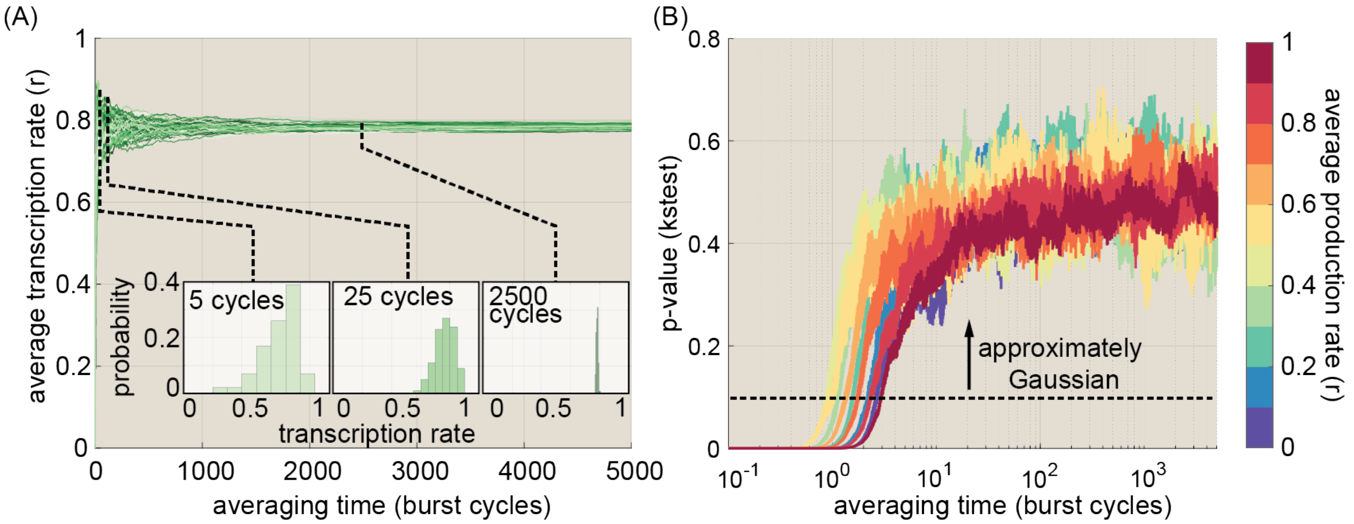

Fig. S6. Testing the validity of the Gaussian noise approximation. **(A)** Illustrative plot showing average transcription rate as a function of the averaging time across 100 stochastic simulations of one illustrative realization of the four-state model gene circuit. Inset histograms show distribution of apparent rates at three different time points. We see that, as the accumulation time increases, the distributions get tighter and appear more Gaussian in shape. **(B)** Plot showing p-values of one-sample Kolmograv-Smirnov test. Different colors indicate average trends for systems with different average transcription rates. We see that systems near the low and high ends of the induction curve converge to Guassian form most slowly, but even these cross the $p = 0.1$ line within a handful of burst cycles. Error bars indicate bootstrap estimates of standard error calculated for each group. (For stochastic simulations shown in A and B, transition rate and interaction term magnitudes, k and η, were constrained such that $10^{-2} \leq k\tau_b \leq 10^2$ and $10^{-2} \leq \eta \leq 10^2$, where τ_b is the burst cycle time. η_{ab} and η_{ib} were further constrained such that $\eta_{ab} \geq 1$ and $\eta_{ib} \leq 1$, consistent with our assumption that the transcription factor activates the gene locus.)

C. Deriving the rate of information transmission for a gene locus. Motivated by (9), we define the rate of information transmission as the time derivative of the expected Kullback-Leibler (KL) divergence between the two hypotheses ($C = c_0$ and

$C = c_1$), given some accumulated mRNA level m, such that

$$\text{IR} = \frac{d}{dt}\Big\langle \text{D}_{\text{KL}}\big[p(c_1|m)||p(c_0|m)\big]\Big\rangle, \tag{S25}$$

where $\text{P}(c_0|m)$ and $\text{P}(c_1|m)$ indicate (respectively) the conditional likelihood that the true value of C is c_0 and c_1 given the observed output m, and where the angled brackets indicate that we are dealing with the expected value of D_{KL} across many replicates. We refer readers to information theory reference materials for a formal definition of D_{KL} (see, e.g., (10)); however, at an intuitive level it can be regarded as measuring how different two probability distributions are from one another. Thus, with Equation S25, we define the rate of information production as the rate at which the two possibilities (c_1 or c_0?) become distinguishable from one another given the observed "evidence" (m).

We can write out the expected KL divergence from Equation S25 more explicitly as the weighted sum of log probability ratios:

$$\text{IR} = \frac{d}{dt}\left(p_0\left\langle \ln\frac{p(c_0|m)}{p(c_1|m)}\right\rangle_0 + p_1\left\langle \ln\frac{p(c_1|m)}{p(c_0|m)}\right\rangle_1\right), \tag{S26}$$

where $\langle ...\rangle_i$ indicates the expectation taken assuming the true value of C to be c_i and where p_0 and p_1 indicate the priors on the true value of C, taken to be equal moving forward ($p_1 = p_0 = 1/2$). This formulation provides intuition for the sense in which IR is the information rate: as the conditional probabilities of the observed output given the true (numerators) and false (denominators) hypotheses about C diverge in favor of the true hypothesis, the log ratio terms will become large and positive. Thus a positive derivative corresponds to positive information production.

However, here we must recall that our focus here is to understand how the molecular architecture of gene loci impacts the transcriptional response and, ultimately, IR. Thus we wish to work in terms of $p(m|c)$—the conditional distribution of observed mRNA outputs given some input—rather than $p(c|m)$. To do this, we make use of Bayes' Theorem. We have:

$$\frac{p(c_0|m)}{p(c_1|m)}\frac{p(m)}{p(m)} = \frac{p(m|c_0)}{p(m|c_1)}\frac{p(c_0)}{p(c_1)}. \tag{S27}$$

This expression becomes an equality if we assumed equal prior probabilities for our two hypotheses ($p(c_0) = p(c_1)$):

$$\frac{p(c_0|m)}{p(c_1|m)} = \frac{p(m|c_0)}{p(m|c_1)}. \tag{S28}$$

Thus, we can use Equation S28 to rewrite Equation S26 as:

$$IR = \frac{d}{dt}\frac{1}{2}\left(\left\langle \ln\frac{p(m|c_0)}{p(m|c_1)}\right\rangle_0 + \left\langle \ln\frac{p(m|c_1)}{p(m|c_0)}\right\rangle_1\right). \tag{S29}$$

We can think of the conditional probabilities, $p(m|c_i)$, in Equation S29 as representing the full *stochastic* transcriptional response to some input activator concentration c_i. When these are approximately Gaussian (a condition discussed above in Appendix B), it becomes a straightforward exercise to solve for the expected log ratios in Equation S29. We will solve for the case when $C = c_1$ in full. The c_0 case proceeds in precisely the same fashion. To start, we have

$$\left\langle \ln\frac{p(m|c_1)}{p(m|c_0)}\right\rangle_1 = \int_0^\infty p(m|c_1)\ln p(m|c_1)dg - \int_0^\infty p(m|c_1)\ln p(m|c_0)dg. \tag{S30}$$

Recall that $m = rt$ is Gaussian with probability density function:

$$p(m|c_i) = \frac{e^{-\left(\frac{m-\overline{m}(c_i)}{2\sigma^2(c_i)}\right)^2}}{\sqrt{2\pi\sigma^2(c_i)}}. \tag{S31}$$

Plugging Equation S31 in for $\ln p(m|c_1)$ yields

$$\left\langle \ln\frac{p(m|c_1)}{p(m|c_0)}\right\rangle_1 = -\frac{1}{2}\ln\left(2\pi\sigma_m^2(c_1)\right) - \frac{1}{2} - \int_0^\infty p(m|c_1)\left[-\frac{1}{2}\ln\left(2\pi\sigma_m^2(c_0)\right) - \frac{1}{2}\left(\frac{\overline{m}(c_0)-g}{\sigma_m(c_0)}\right)^2\right]dm, \tag{S32}$$

Where we've recognized that the first integral will simply yield the standard expression for the entropy of a Gaussian random variable. Pulling constant factors out of the second integral leads to

$$\left\langle \ln\frac{p(m|c_1)}{p(m|c_0)}\right\rangle_1 = -\frac{1}{2}\ln\left(2\pi\sigma_m^2(c_1)\right) - \frac{1}{2} + \frac{1}{2}\ln\left(2\pi\sigma_m^2(c_0)\right) + \frac{1}{2\sigma_m^2(c_0)}\int_0^\infty p(m|c_1)\left[\overline{m}^2(c_0) - 2m\overline{m}(c_0) + m^2\right]dm. \tag{S33}$$

Simplifying and recognizing that $\langle m^2\rangle_1 = \overline{m}^2(c_1) + \sigma_m^2(c_1)$ leads to:

$$\left\langle \ln\frac{p(m|c_1)}{p(m|c_0)}\right\rangle_1 = \frac{1}{2}\ln\frac{\sigma_m^2(c_1)}{\sigma_m^2(c_0)} - \frac{1}{2} + \frac{1}{2\sigma_m^2(c_0)}\left[\overline{m}^2(c_0) - 2\overline{m}(c_0)\overline{m}(c_1) + \sigma_m^2(c_1)^2 + \overline{m}^2(c_1)^2\right]. \tag{S34}$$

Finally, we recall that $m = rt$ and $\sigma_m^2 = \sigma^2 t$, obtaining

$$\left\langle \ln \frac{p(m|c_1)}{p(m|c_0)} \right\rangle_1 = \frac{1}{2}\left[\ln\frac{\sigma_r^2(c_0)}{\sigma_r^2(c_1)} + t\frac{\big(\bar{r}(c_1)-\bar{r}(c_0)\big)^2}{\sigma_r^2(c_0)} + \frac{\sigma_r^2(c_1)}{\sigma_r^2(c_0)} - 1\right]. \quad [S35]$$

Performing the same procedure for the case where $c = c_0$ yields:

$$\left\langle \ln \frac{p(m|c_0)}{p(m|c_1)} \right\rangle_0 = \frac{1}{2}\left[\ln\frac{\sigma_r^2(c_1)}{\sigma_r^2(c_0)} + t\frac{\big(\bar{r}(c_0)-\bar{r}(c_1)\big)^2}{\sigma_r^2(c_1)} + \frac{\sigma_r^2(c_0)}{\sigma_r^2(c_1)} - 1\right]. \quad [S36]$$

Plugging Equation S35 and Equation S36 into Equation S29 and taking the derivative with respect to time yields

$$\mathrm{IR} = \frac{1}{4}\frac{\big(\bar{r}(c_1)-\bar{r}(c_0)\big)^2\big(\sigma(c_1)^2+\sigma(c_0)^2\big)}{\sigma(c_0)^2\sigma(c_1)^2}. \quad [S37]$$

Next, if we assume that the difference between c_0 and c_1 is small (as stipulated in the main text), then $\sigma(c_0) \approx \sigma(c_1) \approx \sigma^2(c^*)$ and $\bar{r}(c_1) - \bar{r}(c_0) \approx \delta c dr/dc$, leading to

$$\mathrm{IR} = \frac{1}{2}\left(\delta c\frac{dr}{dc}\right)^2\frac{1}{\sigma(c^*)^2}. \quad [S38]$$

Finally, we invoke the definitions of sharpness and precision given in Figure 1B, which leads to Equation 2 from the main text:

$$\mathrm{IR} = \frac{1}{2}\left(\frac{\delta c}{c^*}\right)^2 s^2 p^2. \quad [S39]$$

D. Poisson noise from mRNA synthesis is negligible relative to noise from bursting. In this section, we provide support for the claim, made in Main Text Section B, that Poisson noise due to mRNA synthesis is negligible relative to noise from transcriptional bursting. We take as our starting point Equation S71 from Appendix K,

$$\mathrm{P} = \frac{\pi_\mathrm{a}(1-\pi_\mathrm{a})}{\sigma}, \quad [S40]$$

which relates the normalized precision, P, to the bursting noise, σ, and the fraction of time a gene circuit spends in transcriptionally active states, π_a. From Figure 3A, we see that $P \leq 1$ for the four-state gene circuit shown in Figure 1C when the system is out of equilibrium, which, from Equation S40, implies that

$$\sigma^2 \geq \pi_\mathrm{a}^2(1-\pi_\mathrm{a})^2 \quad [S41]$$

for the 4 state system.

Thus, Equation S42 gives a lower bound for the intrinsic variance in gene expression that arises due to transcriptional burst fluctuations at the gene locus. To see how to relate this to noise from mRNA synthesis, we need to take two more steps. First, we must recall that we are working in units of the burst cycle time, τ_b. Second, we must further recall that we set the actual rate of mRNA synthesis, r_0, equal to 1 throughout the main text. We must do away with these simplifications in order to relate σ^2 to synthesis noise. Accounting for these simplifications, the full expression for the noise floor, in "real" time units and accounting for the true rate of mRNA synthesis is

$$\sigma^2_\mathrm{burst} \geq \tau_b r_0^2 \pi_\mathrm{a}^2(1-\pi_\mathrm{a})^2. \quad [S42]$$

Now, if we assume mRNA synthesis to be a Poisson process (following, e.g., (11)), we have that this component of the variance is simply equal to

$$\sigma^2_\mathrm{Poisson} = r_0\pi_\mathrm{a}. \quad [S43]$$

The key thing to notice about Equation S43 is that mRNA synthesis noise is *independent* of the bursting timescale τ_b. Thus, as τ_b increases, σ^2_burst will increase in magnitude relative to $\sigma^2_\mathrm{Poisson}$. Figure S7A and B illustrate this fact, showing predicted bursting and mRNA synthesis variance components, respectively, as a function of the bursting time scale τ_b and the activity level (π_a). All calculations assume an mRNA synthesis rate of 20 mRNA per minute, a rate based off of estimates from the fruit fly (12) and that is consistent with measurements from other systems (13). From Figure S7A, we see that σ^2_burst peaks at $\pi_\mathrm{a} = 0.5$ and increases dramatically as we move rightward along the x-axis and the burst cycle time increases. We emphasize that this represents a *lower* bound for maximally precise non-equilibrium gene circuits; most systems (including IR-optimized systems) will lie above this bound. In contrast Figure S7B shows that noise from mRNA synthesis scales linearly with π_a, and is constant in τ_b.

The total gene expression noise level is given by

$$\sigma^2_\mathrm{tot} = \sigma^2_\mathrm{Poisson} + \sigma^2_\mathrm{burst}. \quad [S44]$$

We can use this expression to calculate a lower bound on the relative contribution of mRNA synthesis noise to the overall intrinsic variance in gene expression. Figure S7C shows the results of this calculation. We see that, with the exception of rapidly bursting systems near the saturation ($\pi_\mathrm{a} \approx 1$), the contribution from Poisson noise due to mRNA synthesis is negligible. Thus, we conclude that noise from transcriptional bursting constitutes the dominant source of gene expression noise for the vast majority of the parameter regimes relevant for the investigations in this paper, and that our decision to neglect Poisson noise from mRNA synthesis is reasonable.

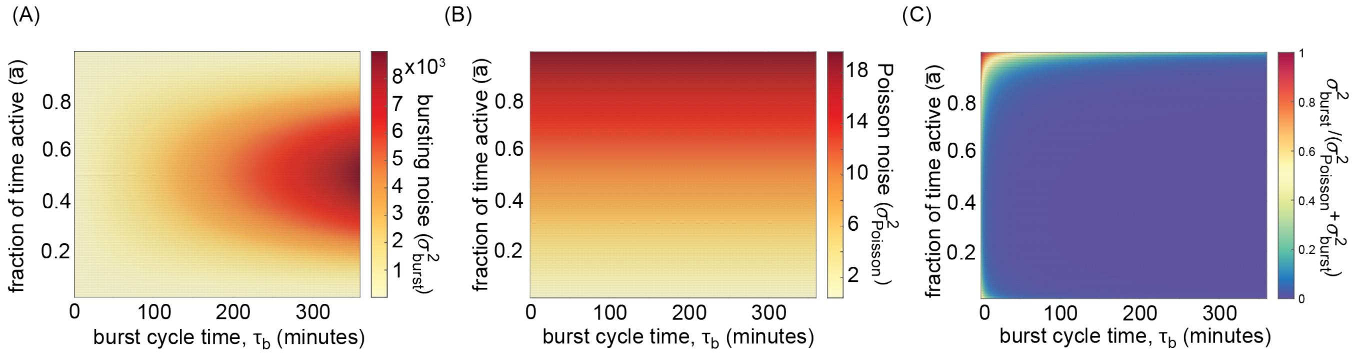

Fig. S7. Determining the contribution from mRNA synthesis noise. (A) Heatmap showing lower bound of bursting component of variance for the non-equilibrium four-state model shown in Figure 1C as a function of the fraction of time spent in the active state (π_a) and the burst cycle time (τ_b). **(B)** Heatmap showing predicted variance component arising from mRNA synthesis. **(C)** Predicted relative contribution of mRNA synthesis noise to total intrinsic noise levels in gene expression. Note that contribution is only significant for rapidly bursting systems near the saturation point. (All calculations assume an mRNA synthesis rate of 20 per minute, in keeping with estimates from (5).)

E. The Sequential Probability Ratio Test. Over half a century ago, Wald conceived of the Sequential Probability Ratio Test (SPRT) as a solution to the problem of making accurate decisions between two hypotheses, H_1 and H_0 in "real time" as relevant data is accruing (14). Shortly thereafter, it was established that SPRT represents the optimal approach to sequential decision problems involving binary decisions (15), meaning that it requires the fewest observations to achieve a desired level of accuracy. In this framework, a downstream receiver (in our case, downstream genes or other cellular processes) tracks the accrual of some signal (mRNA, and eventually protein) over time and compares how likely this accrued signal is under the two hypotheses to be distinguished (e.g., high or low activator concentration). In this work, we use the optimal nature of SPRT to set lower bounds on decision times that could be achieved given the transcriptional output of model gene loci. The essence of the test lies in tracking the relative likelihoods of our two hypotheses ($C = c_1$ and $C = c_0$) over time as more and more transcriptional output, m, accrues:

$$\frac{P_0}{P_1} = \frac{P(c_0|m)}{P(c_1|m)} \qquad \text{[S45]}$$

Figure S8A shows a stochastic simulation of how this ratio evolves over time for the output of a single model gene circuit. Although the true concentration in this case is c_0, we see that the two hypotheses are essentially indistinguishable early on. This is because the range of possible outputs given high and low activator concentrations overlap significantly early on (leftmost panel of Figure S8B). However, as more and more time passes, the expected outputs (m) given the two possible inputs (c_1 and c_0) start to separate. We see that the ratio in their likelihoods diverges more and more in favor of c_0 ($P_0/P_1 >> 1$), corresponding to a higher and higher degree of certainty that c_0 is the correct choice.

This divergence, however, is non-monotonic and noisy, which reflects the stochastic nature of protein production at a single gene locus. It has been shown that the noisy divergence of the log of the probability ratio (which we will call $\mathscr{L}$) can modeled as a 1-D diffusive process with average drift IR (9) given by

$$\mathrm{IR} = \frac{d}{dt}\langle \mathscr{L} \rangle. \qquad \text{[S46]}$$

In this framework, a "decision" is made when $\mathscr{L}$ crosses a so-called "decision boundary" (horizontal dashed lines in Figure S8A). Siggia et al showed that the Gaussian diffusion approximation could be used to obtain an analytic expression for the expected time needed to make a decision. From Equation 15 in the supplement of (9), we have that:

$$\langle T \rangle = \frac{K}{2V \sinh \frac{VK}{D}} \left[e^{\frac{VK}{D}} + e^{-\frac{VK}{D}} - 2 \right], \qquad \text{[S47]}$$

where V is the same as IR from above (and in the main text), D encodes the diffusivity of decision process (essentially, how large the fluctuations are about its mean drift trajectory), and K is related to the log of the error tolerance parameter ε, such that

$$K = \log \left(\frac{1-\varepsilon}{\varepsilon} \right). \qquad \text{[S48]}$$

We note that Equation S47 assumes equal priors regarding the likelihood of c_1 and c_0, and also assumes equal error tolerances for choosing incorrectly in either case (16).

If we take the accumulated transcriptional output of our gene circuit, $m = rt$, to be approximately Gaussian (see Appendix B), then it can be shown that D has the form:

$$D = \frac{(m_0 - m_1)^2(\sigma_0^6 + \sigma_1^6)}{4\sigma_0^4\sigma_1^4}, \qquad \text{[S49]}$$

where m_i and σ_i give the mean and variance in the accumulated transcriptional output, given that $C = c_i$. From Equation S37 in Appendix C, we also have that

$$V = \text{IR} = \frac{(m_0 - m_1)^2(\sigma_0^2 + \sigma_1^2)}{4\sigma_0^2\sigma_1^2}. \quad \text{[S50]}$$

In a previous work, Desponds and colleagues (16) demonstrated that $D \approx V$ when the difference between hypothesis—$\delta c/c^*$ in our case—is small. Indeed, we see from Equations S49 and S50, that when c_1 and c_0 are sufficiently close, σ_1 and σ_0 will be approximately equal, such that:

$$D \approx V \approx \frac{(m_0 - m_1)^2}{2\sigma^2}. \quad \text{[S51]}$$

As demonstrated by (16), when $D \approx V$, Equation S47 simplifies dramatically, yielding

$$\langle T \rangle = \log\left(\frac{1-\varepsilon}{\varepsilon}\right)\frac{1-2\varepsilon}{\text{IR}}, \quad \text{[S52]}$$

which is Equation 3 from the main text. For correctness, we use the full expression (Equation S47) to calculate all decision time quantities shown in the main text. However, since Equation S52 holds quite well for the 10% concentration difference considered here, we give the simpler expression in the main text to aid the reader's intuition.

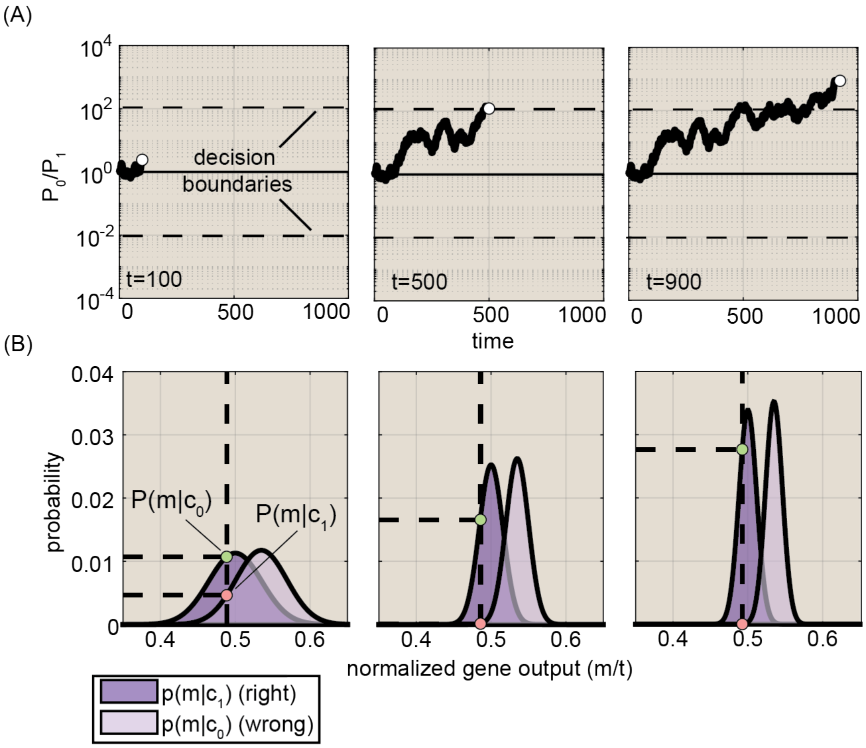

Fig. S8. The Sequential Probability Ratio Test. (A) Panels show stochastic trajectory of the relative probabilities of c_0 and c_1 over time, given the observed output of some gene circuit (illustrated in Figure 1E). **(B)** Panels illustrating expected distributions of transcriptional outputs, m, for each concentration at different time points normalized by the total time over which the gene circuit has been active, t. Note how the distributions narrow and separate as time progresses.

F. Steady state approximation. Throughout this work, we assume that microscopic reactions within our model gene circuits have reached steady state, such that we can employ the closed form solutions for steady-state system behaviors detailed in Appendix A. In this Appendix, we test this assumption by using stochastic simulations to test whether key gene circuit behaviors converge to their steady state behaviors rapidly relative to the cellular decision timescales considered in this work (e.g., in Figure 4C and D). In particular, we test the convergence of the average production rate ($\bar{r}$ from Equation S8) and the variance (σ^2 from Equation S10), since these two quantities form the basis for the information rate and decision time calculations presented throughout this work.

As in Appendix B, we used stochastic simulations to test how rapidly the average production rate and variance of the accumulated transcriptional output approaches our steady-state predictions. We tracked the output of 500 random realizations of the four-state system shown in Figure 1C for 5,000 burst cycles. Each realization had a unique set of transition rates and, correspondingly, a unique average rate of transcription, $\bar{r}$, and variance, σ^2. For each model realization, we ran 100 stochastic simulations. We used these simulations to calculate the average transcription rate and variance for each model realization as function of mRNA accumulation time (in burst cycles).

To assess the rate at which the actual mean and variance of the accumulated mRNA output produced by our model gene circuits converge to their predicted steady state values, we calculated the percent error for each of metric as a function of accumulation time. Specifically, for each of our 500 model realizations, we calculated the percent error in the mean at time t as

$$\epsilon_r^{(i)}(t) = \left| \frac{\overline{r}_i - \hat{r}_i(t)}{\overline{r}_i} \right| \times 100, \quad \text{[S53]}$$

where $\overline{r}_i$ is the predicted steady state mRNA production rate for the ith gene circuit realization, and where $\hat{r}_i(t)$ is the empirical estimate derived from averaging across the 100 simulations of gene circuit i up to time t. In analogous fashion, we calculate the percent error in the variance as

$$\epsilon_\sigma^{(i)}(t) = \left| \frac{\sigma_i - \hat{\sigma}_i^2(t)}{\sigma_i} \right| \times 100. \quad \text{[S54]}$$

As a final step in constructing our error metrics, we must account for errors in our simulation-based estimates that are due to statistical noise, rather than to a lack of steady-state convergence. This statistical noise results from the fact that we use a finite number of simulations (100) to estimate $\hat{r}_i$ and $\hat{\sigma}_i^2$. This leads to noise that has nothing to do with the question of steady state convergence. To account for this, we calculate asymptotic values for ϵ_r and ϵ_σ (taken as their values after 5000 burst cycles) and subtract them from our percent error estimates. The idea behind this is that, because the systems will have definitively reached steady state by 5000 burst cycles, any residual error will reflect statistical uncertainty in the Monte Carlo estimator, which is irrelevant for assessing steady-state convergence. This gives

$$\delta_r^{(i)}(t) = \epsilon_r^{(i)}(t) - \epsilon_r^{(i)}(5000) \quad \text{[S55]}$$

for the mean production rate and

$$\delta_\sigma^{(i)}(t) = \epsilon_\sigma^{(i)}(t) - \epsilon_\sigma^{(i)}(5000) \quad \text{[S56]}$$

for the variance.

Figure S9A shows the average adjusted percent error in the mRNA production rate (Equation S55) for 10 different sets of gene circuits, grouped according to their average rate of transcription. The fastest biological timescale cited in the main text is that of cells in the developing Drosophila *melanogaster* embryo, which have approximately 32.5 burst cycles to distinguish between c_0 and c_1. We find that, by this point, the mean derived from our simulations is already in excellent agreement with the steady state predictions derived from Equation S8, with an average error of just 1%. Indeed, all 10 gene circuit groups shown in Figure S9A have $\delta_r < 5\%$ by just 10 burst cycles into the mRNA accumulation process.

To gain better intuition for how to interpret these error levels, it is informative to directly compare predicted and simulated production rate values. The inset panel of Figure S9A shows a scatter plot of simulated vs. steady state values for all 500 gene circuits after 32.5 (green circles) and 5000 (gray diamonds) burst cycles. These plots indicate excellent agreement in each case, confirming our steady state predictions accurately reflect the mean production rate after 32.5 burst cycles of mRNA accumulation.

Figure S9B shows the average adjusted percent error in the variance in mRNA production rate (Equation S56) for the same 10 different sets of gene circuits. Here again, we observe a rapid convergence to the predicted steady state values predicted by Equation S10, with $\delta_\sigma < 10\%$ for all 10 groups after just 10 burst cycles, and with an overall adjusted percent error of just 1% after 32.5 burst cycles. The inset panel of Figure S9B shows a scatter plot of simulated vs. steady state variance values for all 500 gene circuits after 32.5 (green circles) and 5000 (gray diamonds) burst cycles. As with the production rate, these plots indicate good agreement between steady state predictions and our simulation results.

G. Implementation of parameter sweep algorithm. In this section, we describe the parameter sweep algorithm employed throughout this work to enumerate the performance bounds of gene circuit models. We note that this approach is based on an algorithm previously employed by Eck & Liu et al. (17) to explore the behavior of non-equilibrium models of transcription (see also, (18)). Figure S10A illustrates the key steps in this numerical procedure. First, an initial set of gene circuit realizations (typically comprised of 1,000 variants) is generated by sampling random values for each transition rate in the system. We then calculate the performance metrics of interest (S and P for the example in Figure S10A) for each gene circuit realization. This defines an initial set of points (Figure S10A, Panel i) that collectively span some region in 2D parameter space with area a_1.

Next (Panel ii), we subdivide parameter space into N different bins along the X and Y axes, with N dictated by the total number of points ($10 \leq N \leq 50$). We subsequently calculate the maximum and minimum point in each X and Y slice (Panel iii). Finally, we randomly select candidate gene circuit models from these boundary points and apply small perturbations to each transition rate to generate a new set of random variants (iv). In general, these variants will lie close to the original model in 2D parameter space and, thus, close to the current outer boundary of parameter space. The key to the algorithm's success is that some of these variants will lie *beyond* the current boundary (blue points in Figure S10A, Panel iv). This has the effect of extending the boundary outward, leading to an increase in the surface area spanned by our sample points (panel iv). As a result, cycling through steps ii-iv amounts to a stochastic edge-finding algorithm that will iteratively expand the boundary spanned by sample points outward in 2D parameter space until some analytic boundary is reached.

The panels in Figure S10B show snapshots of the sweep algorithm's progress exploring sharpness vs. precision parameter space for non-equilibrium realizations of the four-state gene circuit (Figure 1C). Figure S10C shows the total area spanned by the sample points for this run as a function of sweep iteration. By eye it appears that most of salient parameter space has been explored by step 10 of the algorithm, but we are quite strict with our convergence criteria. We will only terminate a sweep at

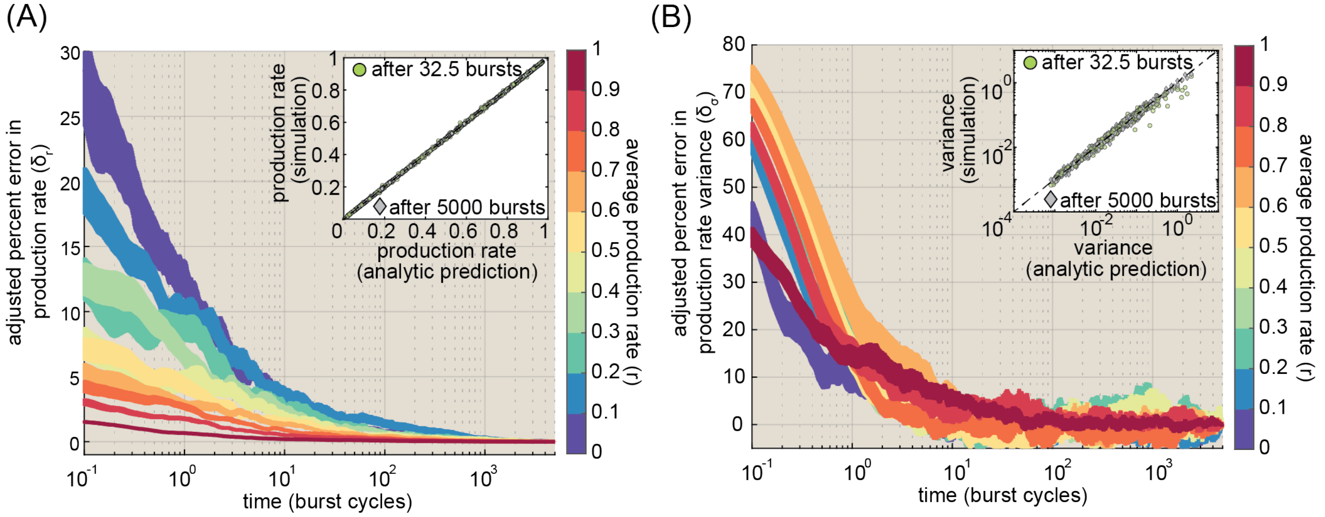

Fig. S9. Testing timescale for convergence to steady-state. (A) Trends showing adjusted percent deviation of simulation results for the mean production rate from predicted steady-state values (from Equation S8) plotted as a function of the number of burst cycle times. Colors indicate trends for groups of gene circuits with different average production rates. Shaded regions indicate bootstrap estimates of the standard error in the estimated percent errors. The inset panel shows production rates calculated from simulations for each of the 500 random gene circuit realizations after 32.5 (green circles) and 5000 (gray diamonds) burst cycles plotted against their predicted steady-state values. Dashed black line indicates expected trend assuming perfect agreement between simulation and prediction. **(B)** Trends showing adjusted percent deviation of simulation results for the variance in the production rate from predicted steady-state values (from Equation S10) as a function of the number of burst cycles. Inset panel shows variance calculated from simulations for each of the 500 random gene circuit realizations after 32.5 (green circles) and 5000 (gray diamonds) burst cycles plotted against their predicted steady-state values. Dashed line indicates expected trend assuming perfect agreement between simulation and prediction. (For stochastic simulations shown in A and B, transition rate and interaction term magnitudes, k and η, were constrained such that $10^{-5} \leq k\tau_b \leq 10^5$ and $10^{-5} \leq \eta \leq 10^5$, where τ_b is the burst cycle time. η_{ab} and η_{ib} were further constrained such that $\eta_{ab} \geq 1$ and $\eta_{ib} \leq 1$, consistent with our assumption that the transcription factor activates the gene locus.)

step t if $(a_t - a_{t-2})/a_{t-2} \leq 0.001$ and $(a_{t-1} - a_{t-3})/a_{t-3} \leq 0.001$. In this case, this convergence criterion is met following step 25, leading to the final set of sample points shown in Figure S10D. In general, we run all sweeps until the above criterion is met or some pre-specified maximum number of iterations (usually 50) is reached.

G.1. Numeric vs. symbolic metric calculations. The algorithm outlined in Figure S10A is predicated upon the ability to rapidly calculate performance metric quantities (e.g., S and IR) given a set of transition rate magnitudes. Wherever possible, we use symbolic expressions to perform these calculations; however, this is only feasible for the simple four and six state systems depicted in Figure 1C and Figure 4A. For more complex models, it is infeasible to perform the symbolic operations required to obtain closed-form symbolic expressions. As a results, we use numerical calculations to arrive at performance metrics for all higher-order models.

G.2. Enforcing equilibrium constraints. In this work, we make frequent use of comparisons between equilibrium and non-equilibrium gene circuits in order to elucidate how energy expenditure alters gene-regulatory performance. A key step in performing parameter sweeps for equilibrium gene circuits is ensuring that transition rates adhere to the constraints imposed by detailed balance. For the simple four state model shown in Figure 1C, this process boils down to ensuring that the product of the four transition rates moving in a clockwise direction about the square is equal to the product of the four counterclockwise rates. As shown in Appendix A.2, this amounts to enforcing the constraint that

$$\lambda = \frac{\eta_{ab}\eta_{ua}}{\eta_{ba}\eta_{ib}} = 1, \tag{S57}$$

where the η factors on the top and bottom of the left-hand-side expression correspond to regulatory interaction terms that modify transition rates in the clockwise and counterclockwise directions, respectively, and where λ is the flux factor that captures the relative magnitudes of clockwise and counterclockwise transitions.

To enforce this constraint during the course of a parameter sweep, we add a step to the process outlined above. New gene circuit realizations are generated as before, but now, following its generation, we calculate the initial flux factor, λ_0 for each new realization using Equation S57. In general this quantity will not equal one for the new realizations ($\lambda^* \neq 1$). To fix this, we then multiply η_{ba} and η_{ib} each by a factor of $\lambda^{\frac{1}{2}}$, which leads to a modified system that adheres to the constraint laid out in Equation S57. Next, we check the modified terms to ensure that they adhere to magnitude constraints (typically $10^{-5}/\tau_b \geq \eta_i$ $10^{-5}/\tau_b$) and pass all qualifying rates along to the next step in the sweep iteration (step ii in Figure S10A). Finally, we note that, although we have focused on the simple four state system, our assumption that all binding and activation reactions are identical ensures that the exact same approach holds for all higher-order models ($N_A > 1$ or $N_B > 1$) considered in this work. See Appendix J.3 for details.

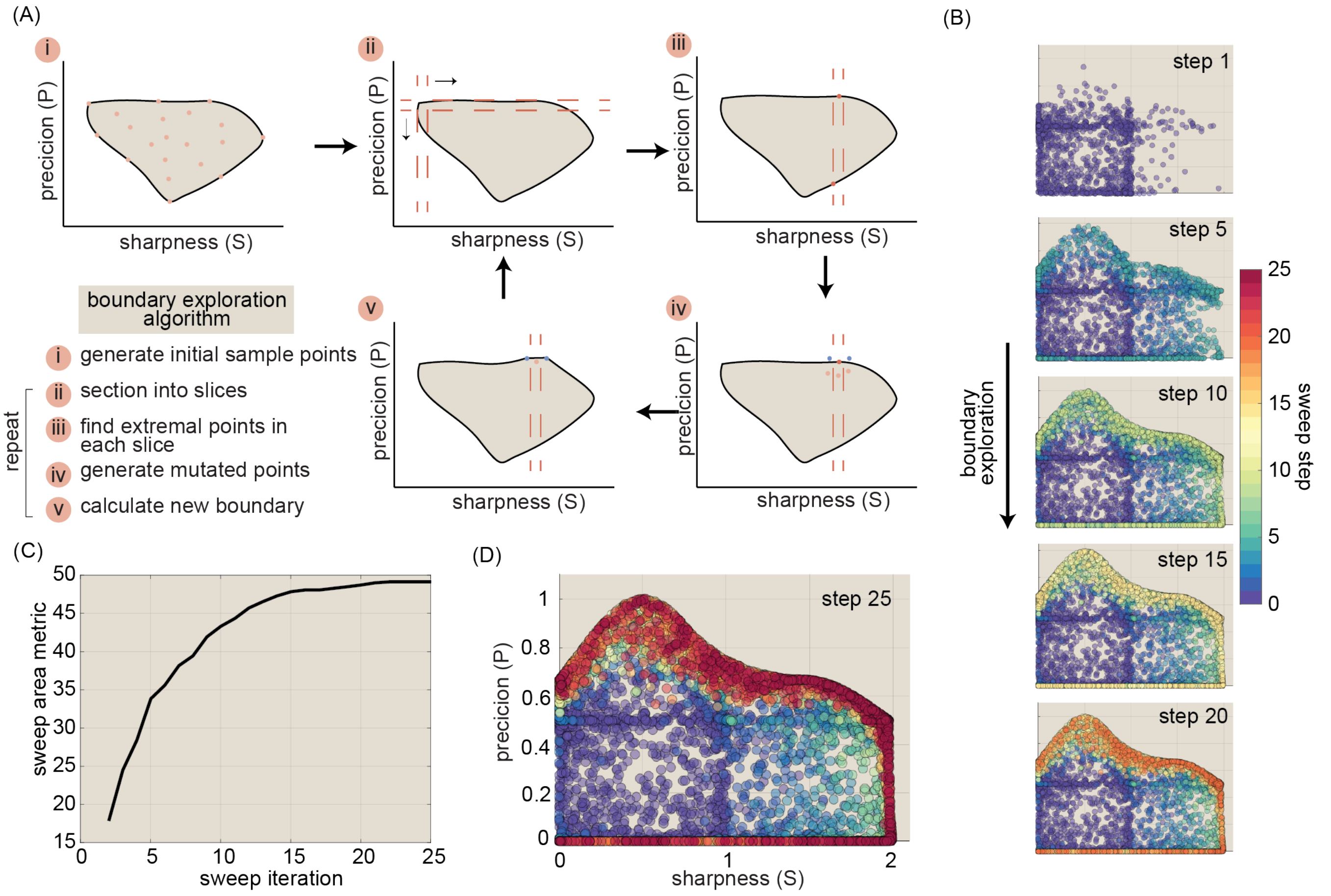

Fig. S10. A simple stochastic edge-finding algorithm for numerical parameter sweeps. (A) Schematic illustrating key steps in our parameter sweep approach (see text for details). This panel has been adapted with permission from (17). **(B)** Sequence of snapshots showing progress of sweep algorithm across a single run for the case of normalized sharpness (S) versus normalized precision (P). Circle color indicates the sweep step on which it was generated. **(C)** Plot showing 2D surface area spanned by sample points over time. **(E)** Plot showing final set of sample points obtained by the sweep algorithm.

H. Testing the convergence characteristics of the parameter sweep algorithm. Here, we discuss results from a series of tests designed to assess the convergence of our sweep algorithm for key scenarios examined in the main text. This task is the most straight-forward when the algorithm is employed for "two-boundary" sweeps, such as S vs. P (Figure 3A) and S_0 vs f (Figure 5B), where both parameters examined adhere to finite performance bounds and, thus, where the 2D region of accessible parameter space has a finite area. In this case, our general approach will be to assess whether independent runs of the algorithm (i) converge prior to the 50 run limit and (ii) reach a consistent final estimate for the area of 2D space that is attainable for different model architectures. The task becomes more complicated for "one-boundary" sweeps, such as IR vs. Φ (Figure 2A, C, and D) and IR vs. w/c, where only a single parameter (IR in each case) has a finite upper bound and the other (Φ and w/c) is limited only by bounds imposed externally as a part of sweep specification. We will begin by assessing convergence for the simpler two-sided case, and will turn thereafter to examining one-sided cases.

H.1. Sharpness vs. Precision sweeps. Figure 3A and Figure S2A and B show results for parameter sweeps examining tradeoffs between normalized sharpness (S) and normalized precision (P) for systems with 1-5 binding sites and 1-4 activation steps. We note that Figure 3B and Figure S2C also derive from these parameter sweep results. Across the board, we find that nearly all independent runs of the sweep algorithm converge according to the definition laid out above (Figure S11A and B). Moreover, for simpler architectures, we find that all independent sweep runs converge to essentially the same total area. For instance, Figure S11B shows normalized area as a function of sweep step for 500 non-equilibrium realizations of the baseline four-state model, indicating that all runs terminate near the global maximum found across all runs (dashed line). We take this as strong evidence that the algorithm is consistently exploring the full extend of 2D parameter space.

As might be expected, the task of exhaustively exploring parameter space becomes more difficult as models become more complex. Note the larger spread in outcomes for the non-equilibrium five binding site ($N_B = 5$) and 4 activation steps ($N_A = 4$) models in Figure S11C and D, respectively. Nonetheless, we find that a significant number of sweeps converge to a consistent maximum area, even for the most complex models considered. Figure S11E and F give the total fraction of sweeps having a final area within 95% of the global maximum as a function of binding site number and activation step number, respectively. First, we see that *100%* of sweeps for equilibrium models uniformly meet this standard for all model architectures considered

(squares in Figure S11E and F). Second, our analysis indicates that, even for the extrema ($N_B = 5$ and $N_A = 5$), 13% and 36% of total runs, respectively (67 and 179 sweeps), still achieve final areas comparable to the global maximum, suggesting that the algorithm still does an adequate job of exploring parameter space in these cases.

H.2. Information vs. energy sweeps. Next, we turn to the one-sided sweeps. First, let's consider the IR vs. Φ sweep results shown in Figure 2A, C, and D. Because Φ has no natural barrier in parameter space, the convergence metrics considered above do not provide reliable indicators of model convergence. Instead, we make use of the fact that the IR vs. Φ and the S vs. P parameter sweeps should function (either directly or indirectly) to uncover the maximum achievable non-equilibrium information rate for each model architecture. Thus, as a basic test of sweep performance, we checked for the consistency between IR estimates derived from these different sweep modalities. As illustrated in Figure S12A, we find excellent agreement between the maximum IR values derived from the S vs. P (x-axis) and IR vs. Φ (y-axis) parameter sweeps for all model architectures considered. This provides one indication the IR vs. Φ sweeps are fully exploring the relevant parameter space.

As a second check, we compared the IR vs. Φ bounds derived for two separate rounds of parameter sweeps ("round a" and "round b") comprised of 200 and 500 independent parameter sweeps, respectively. We reasoned that, if our algorithm is accurately recovering the true IR vs. Φ bound for each model architecture, this bound (i) should be replicable across different parameter sweep rounds and (ii) should be insensitive to the precise number of sweep runs per round. For each model architecture, we calculated the maximum IR value returned by sweep rounds a and b for 30 different rates of energy dissipation ranging from $0.1k_BT$ (close to equilibrium) to $5000k_BT$ (upper limit of x axis in Figure 2D). Figure S12B and C show the results of this exercise for multi-binding site and multi-activation step models, respectively, indicating excellent agreement between different sweep round for all model architectures. This demonstrates that our information vs. energy bounds are highly replicable across different rounds of sweeps. The consistency across round comprised of significantly different numbers of runs provides further evidence that we are conducting a sufficient number of independent sweeps (≥ 200) per run. Taken together, these results and the results from the preceding paragraph provide strong evidence that our algorithm is robustly recovering accurate IR vs. Φ bounds for all models considered.

H.3. Information vs. w/c sweeps. Finally, we turn to the parameter sweep results for information (and, correspondingly, decision time) as a function of wrong-to-right activator concentration (w/c) shown in Figure 4B-D. We note that the results shown in Figure 5A and C are also derived from these sweeps. Like Φ, w/c has no intrinsic boundary in parameter space and, thus, swept area provides a poor indication of convergence. Fortunately, in addition to treating w/c as a sweep parameter, we can also conduct 2D parameter sweeps where w/c is set at a constant value (e.g., $w/c = 1000$ in Figure S4B). Thus we cross-validate the IR vs. w/c bounds returned by the sweeps from Figure 4 by conducting separate sweeps of IR vs. $\bar{r}$ (the mean transcription rate) at different w/c values (illustrated in Figure S13A). These sweeps *do* converge, with an average of 80% of runs reaching 93% of the global maximum.

Figure S13B and C show the results of this comparison for three different values of w/c: 10, 10^2, and 10^3. We focus on the architectures depicted in Figure 4, namely equilibrium systems with 1-5 binding sites (and one activation step) and non-equilibrium systems with 1-4 activation steps (and one binding site). We also test convergence for the non-equilibrium gene circuit with 5 binding sites and 1 activation step shown as a dashed line in Figure 4C. In most cases, we find good agreement between the two methods, suggesting that the IR vs. w/c sweeps are generally returning accurate estimates for the IR vs. w/c bound. We do note a couple of exceptions, however. First, we see that that IR vs. w/c sweeps appear to underestimate the upper IR bound to a significant degree for the non-equilibrium model with 4 activation steps when $w/c = 10$ (circle in upper right-hand corner of Figure S13C). This indicates that the IR vs. w/c sweep is performing sub-optimally in this case. However, since this deviation occurs in the extreme low interference regime and our focus in Section E lies on model performance at higher w/c levels ($w/c \gtrsim 100$), where our sweep algorithm performs reliably, it does not impact any conclusions drawn throughout the course of the main text. We note that the IR vs w/c sweeps similarly underestimate the IR bound non-equilibrium realizations of the 5 binding site model when $w/c = 10$ (hollow gray circle in upper right-hand corner of Figure S13B). In this case, however, even the IR vs. $\bar{r}$ parameter sweeps do not converge reliably, with only 3-4% of sweeps reaching 95% of the global maximum. Thus, we are unable to assess the full extent to which the IR vs w/c is sub-optimal in this case. Once again, though, this claims in the main text rely only on the IR bound when w/c is large ($w/c \gtrsim 10^3$); a regime in which we find that the sweeps perform reliably (hollow gray square in Figure S13B). Thus, we conclude that the IR vs. w/c sweeps provide a viable basis for the investigations undertaken in this study.

H.4. Specificity vs. N_A results. We claim in the main text that, out of equilibrium, the specificity is bounded by the number of activation steps, such that $f \leq \alpha^{N_A+1}$. Here α is the affinity factor (set to 100) that reflects intrinsic differences in the binding kinetics between cognate and non-cognate factors ($\alpha = k_u^w / k_u$). Figure S4B shows parameter sweep results in support of this claim. These results are derived from 2D f vs. $\bar{r}$ sweeps. Figure S14 shows convergence statistics for these runs for non-equilibrium systems with 1 to 4 activation steps and 1 binding site. We find that all 50 sweep runs met their convergence criteria for each run (Figure S14A) and, further, that no fewer than 76% of runs converged to a 2D area that was within 95% of the global maximum. This indicates that these parameter sweep results converge reliably to consistent overall values for specificity and a function of transcription rate and, thus, that they provide a sound basis for assessing the maximum achievable non-equilibrium specificity as a function of N_A.

I. Estimating decision time ranges for different biological systems.

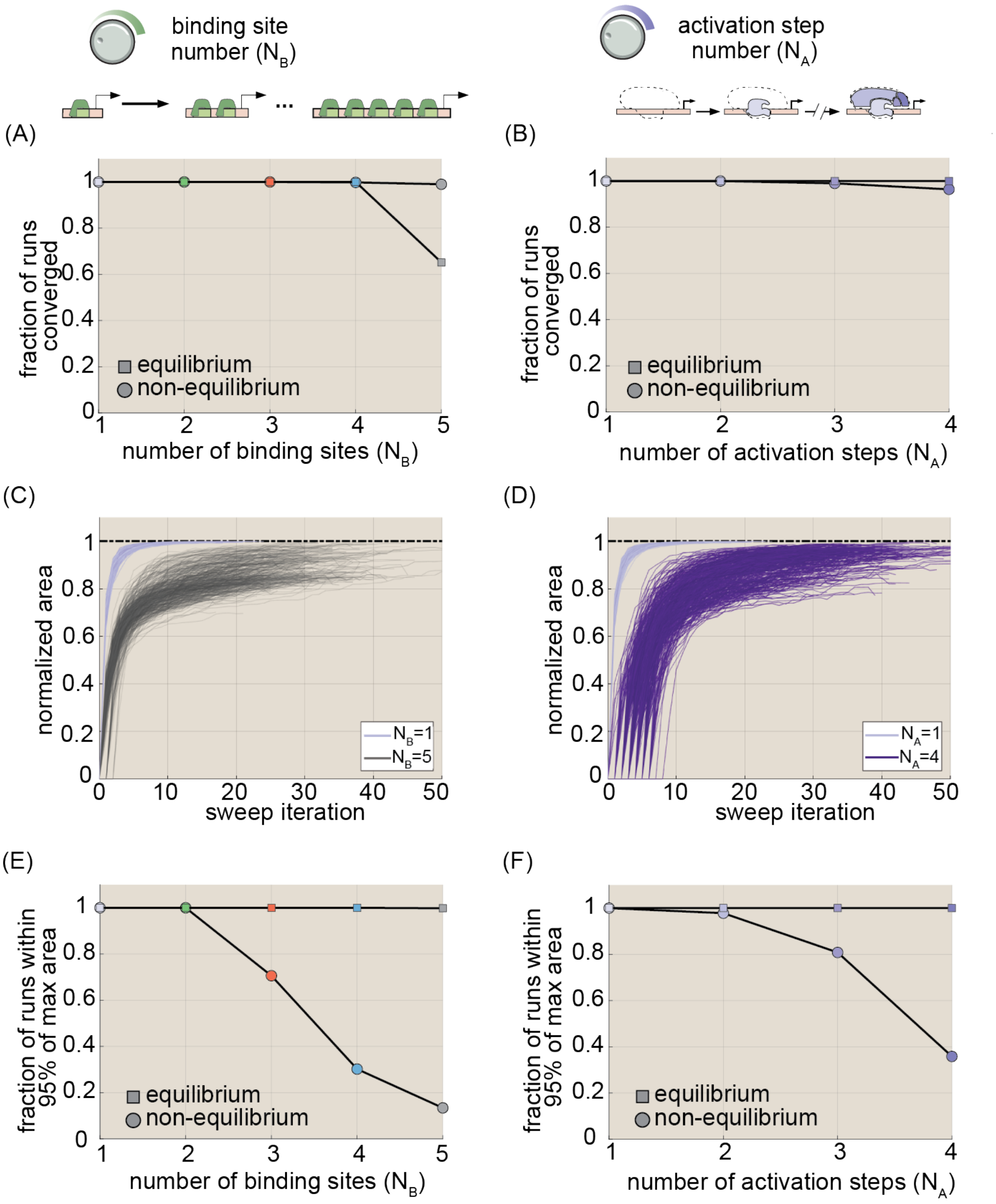

Fig. S11. Convergence results for S vs. P parameter sweeps. (A-B) Plots showing fraction of parameter sweeps that met convergence criteria for multi-binding site and multi-activation step models, respectively. Squares indicate results for equilibrium models and circles indicate non-equilibrium models. **(C-D)** Plots of area vs. sweep step for different model architectures. Note that the area corresponding to the first (iteration=1) is not recorded by the algorithm, and so has been estimated in each case via linear interpolation. Staggered starts apparent for $N_B = 5$ and $N_A = 5$ models indicate cases where model initialization were aborted one or more times due to an insufficient number of gene circuits meeting quality control criteria. **(E-F)** Fraction of parameter sweeps having a final area within 95% of the global maximum for multi-binding site and multi-step models, respectively. (All results were calculated using 500 independent runs of the sweep algorithm for each model architecture. Transition rate and interaction term magnitudes (k and η) were constrained such that $10^{-5} \leq k\tau_b \leq 10^5$ and $10^{-5} \leq \eta \leq 10^5$, where τ_b is the burst cycle time. η_{ab} and η_{ib} were further constrained such that $\eta_{ab} \geq 1$ and $\eta_{ib} \leq 1$, consistent with our assumption that the transcription factor activates the gene locus.)

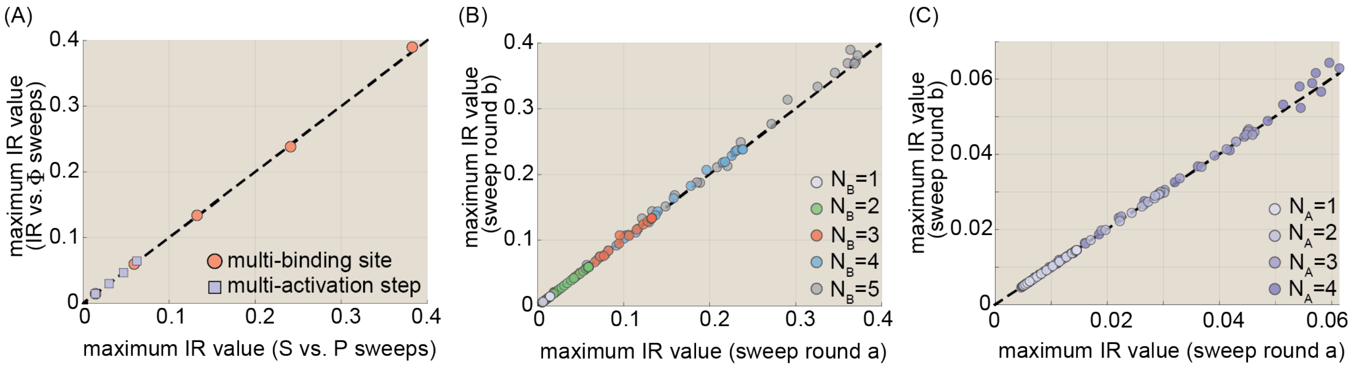

Fig. S12. Convergence results for IR vs. Φ parameter sweeps. (A) Scatter plot comparing maximum information rate estimated from S vs. P and from IR vs. Φ sweeps. **(B-C)** Scatter plots comparing results for the upper IR bound at different points along the curves shown in Figure 2C and D from two independent rounds of parameter sweeps comprising 200 and 500 separate runs, respectively. Points reflect IR maxima for Φ values ranging from 0.1k$_B$T to 5000k$_B$T. (Transition rate and interaction term magnitudes (k and η) were constrained such that $10^{-5} \leq k\tau_b \leq 10^5$ and $10^{-5} \leq \eta \leq 10^5$, where τ_b is the burst cycle time. η_{ab} and η_{ib} were further constrained such that $\eta_{ab} \geq 1$ and $\eta_{ib} \leq 1$, consistent with our assumption that the transcription factor activates the gene locus.)

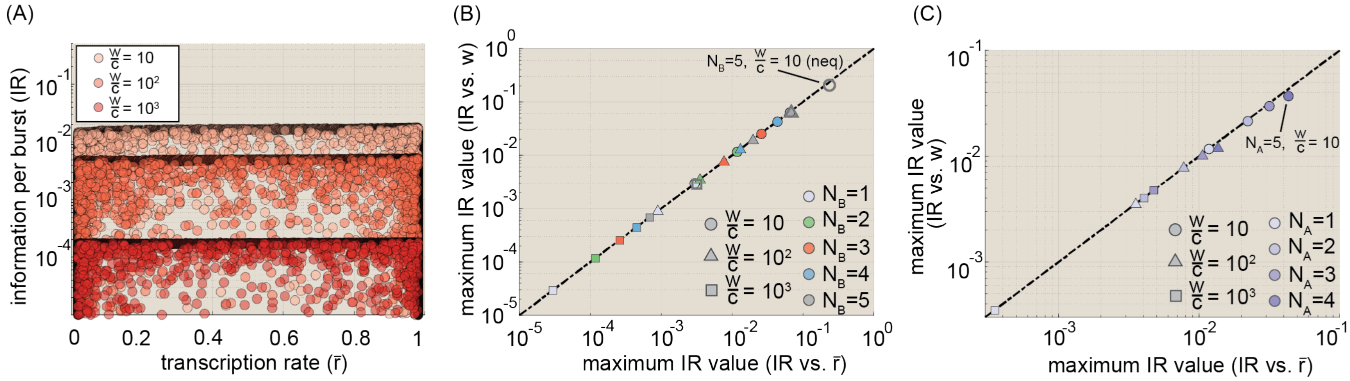

Fig. S13. Convergence results for IR vs. w/c parameter sweeps. (A) Illustrative scatter plot showing IR vs. $\bar{r}$ sweep results for the three binding site model at equilibrium for three different values of w/c. **(B-C)** Scatter plots comparing parameter sweep results for the upper IR bound at three different w/c levels $(10, 10^2$, and $10^3)$ derived from IR vs. $\bar{r}$ sweeps (x-axis) and IR vs. w/c sweeps (y-axis) for equilibrium multi-binding site and non-equilibrium multi-activation step models, respectively. Circles, triangles, and squares indicate w/c values of 10, 10^2, and 10^3, respectively. Hollow markers in (B) indicate non-equilibrium systems. All other results in (B) are for equilibrium gene circuits (in keeping with Figure 4C). All results in (C) correspond to non-equilibrium gene circuits (in keeping with Figure 4D). (Transition rate and interaction term magnitudes (k and η) were constrained such that $10^{-5} \leq k\tau_b \leq 10^5$ and $10^{-5} \leq \eta \leq 10^5$, where τ_b is the burst cycle time. η_{ab} and η_{ib} were further constrained such that $\eta_{ab} \geq 1$ and $\eta_{ib} \leq 1$, consistent with our assumption that the transcription factor activates the gene locus.)

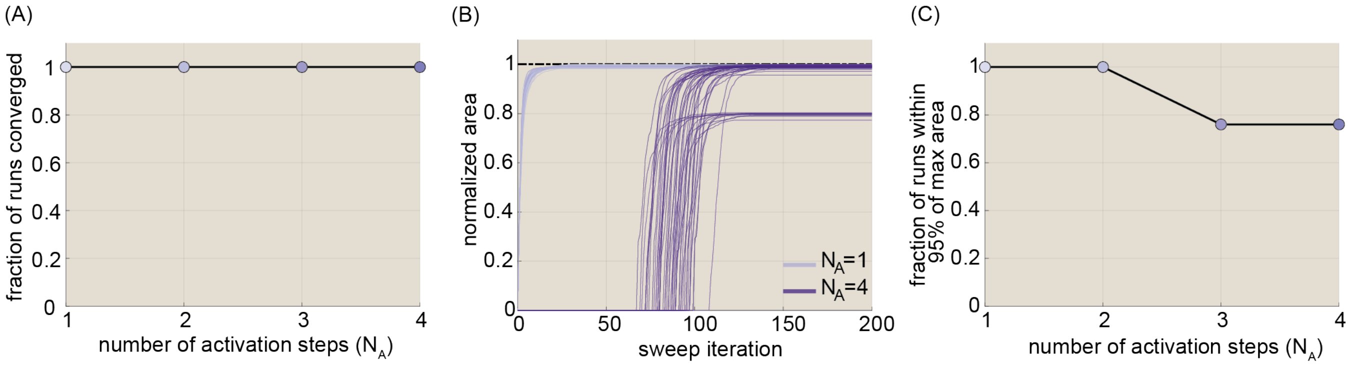

Fig. S14. **Convergence results for f vs. $\overline{r}$ parameter sweeps.** **(A)** Plot showing fraction of runs converged as a function of the number of activation steps. All 50 runs converged for each of the four gene circuit models considered. **(B)** Plot showing area spanned in parameter space as a function of iteration number for all 50 runs for the $N_A = 1$ and $N_A = 4$ models. The delayed rise for $N_A = 4$ models reflects the fact that repeated initializations were required to find a sufficient number of gene circuit realizations that adhered magnitude and quality control constraints. **(C)** Fraction of total runs for each model time that reached a final area greater than or equal to the 95% of the global maximum across all runs. (w/c was set to 10^3 for all runs. Transition rate and interaction term magnitudes (k and η) were constrained such that $10^{-5} \leq k\tau_b \leq 10^5$ and $10^{-5} \leq \eta \leq 10^5$, where τ_b is the burst cycle time. η_{ab} and η_{ib} were further constrained such that $\eta_{ab} \geq 1$ and $\eta_{ib} \leq 1$, consistent with our assumption that the transcription factor activates the gene locus.)

***Caenorhabditis elegans* decision time estimation.** A recent study by Lee and colleagues (19) used live imaging to examine Notch-dependent burst dynamics in the *sygl-1* gene in the germ line of young adult nematodes. Their results indicate that the gene exhibits burst cycle times ranging from 60.5 minutes up to 105.3 minutes (see Figure 2 E and F in (19)). Meanwhile, a review article indicated potential values for the cell cycle time for adult germ-line cells in C. *elegans* as ranging from 16 to 24 hours (20). A separate study examining nonsense-mediated mRNA decay in C. *elegans* reported a half life of approximately 6 hours for the *rpl-7A* gene (Figure 4k in (21)). If we take the cell cycle time as the upper time limit for cellular decision-making, this leads to an estimate of $1440/60.5 = 23.8$ burst cycles.

***Mus musculus* decision time estimation.** Burst cycle time estimates were taken from Table A.1 in Appendix A of (5), which indicates times ranging from 30 minutes to a "few hours". mRNA half life estimates were taken from Table 1 of (22), which indicates a range of 30 minutes to 30 hours for mouse cells. To estimate the effective decision time corresponding to an mRNA half-life of 30 hours (1,800 minutes), we recognize that, once mRNA levels have reached a steady state, they will reflect (in effect) a weighted average of the preceding transcriptional activity, where weights moving backward in time contribute

$$w(t) = e^{-\frac{t}{\tau_{\mathrm{mRNA}}}}, \qquad \text{[S58]}$$

where t indicates temporal distance from the present and τ_{mRNA} is the exponential time constant, given by $\tau_{\mathrm{mRNA}} = t_{\frac{1}{2}}/\ln(2)$. Integrating Equation S58, we find that τ_{mRNA} time steps are effectively present in steady-state mRNA levels. Taking 30 minutes as the lower bound for bursting timescales, this yields an upper bound of $(1800/\log 2)/30 = 86.6$ burst cycles. We note that this estimate is not materially different from the 60 cycle estimate that would be obtained by simply dividing 1,800 by 30.

***Drosophila melanogaster* decision time estimation.** We take the duration of nuclear cycle 14, which follows the thirteenth (and final) round of synchronous cellular divisions in early *Drosophila melanogaster* development, as the relevant timescale for cellular decisions in early fruit fly development. Studies have found that the duration of this developmental period varies along the embryo, with a minimum duration of 65 minutes (23). To estimate bursting timescales, we use burst inference results from our previous work (12), which indicate a burst cycle time of approximately 2 minutes for the *even-skipped* gene. Thus, we arrive at an upper limit of $65/2 = 32.5$ cycles.

J. Higher-order molecular models. Here we provide an overview of key modeling assumptions underlying our approach to modeling gene circuits with multiple activator binding sites or multiple activation steps.

J.1. Gene circuits with multiple activator binding sites. A key feature of eukaryotic enhancers is the presence of multiple distinct binding sites for regulatory factors (24, 25). To better understand the impact of variable numbers of binding sites on information transmission, this work examines gene circuit models with between 1 and 5 activator binding sites. In so doing, we maintain the same basic MWC architecture outlined in the context of the simple 4 state model with one activator binding site shown in Figure 1B. No number of bound activators is alone sufficient for mRNA production, but each contributes an extra factor of η_{ab} and η_{ib} to impact locus activation dynamics. In all cases, we assume a single molecular activation step for multi-binding site models ($\mathrm{N_A} = 1$). Finally, we also allow for cooperative interactions between bound activator molecules, which are captured by the interaction term η_{ub}.

Figure S15A illustrates what this looks like for a model gene circuit with two activator binding sites. The model has eight total states, with four inactive states (top) and four active states (bottom). There are several features to note. First, the transitions between states 2 and 6, which feature two bound activator molecules, are weighted by squared interaction terms, η_{ab}^2 and η_{ib}^2, to reflect the regulatory influence of two activators on locus activation dynamics. More generally, if n activators are bound, these weights are raised to the nth power (i.e. η_{ab}^n and η_{ib}^n). Second, note that unbinding reactions leading out of states 2 and 6 are multiplied by the additional η_{ub} mentioned above. This reflects interactions between bound molecules. For simplicity, we assume that η_{ub} is the same for both cognate and non-cognate activator species, as well as for interactions between cognate and non-cognate activators. In general, unbinding reactions out of states with n activators bound will be weighted by η_{ub}^{n-1} to reflect pairwise interactions from the remaining bound factors.

Lastly, a key simplifying assumption that we make in this work is that each activator binding site is identical with respect to its regulatory influence on the gene locus. As a result, it does not matter *which* binding sites are bound, only *how many* are bound. In the context of Figure S15A, this means that states 1 and 5 are functionally identical to states 3 and 7, respectively. Thus, these states can be combined into single coarse-grained states, which leads to an effective model with 6 states, rather than 8. This ability to coarse grain is invaluable for more complex architectures, since it means that the total number of unique molecular states scales as $\mathrm{N_A N_B}$, rather than $\mathrm{N_A^{N_B}}$.

J.2. Gene circuits with multiple molecular activation steps. Setting $\mathrm{N_A} > 1$ is intended to reflect the reality that multiple distinct molecular reactions—e.g., mediator engagement, PIC assembly, nucleosome displacement, etc.—are necessary preconditions for achieving productive transcription. In the main text we investigate the performance of gene circuits whose transcriptional activity is dictated by 1-4 molecular components, each of which can be either engaged (compatible with transcription) or disengaged (incompatible with transcription). In their simplest interpretation, "engaged" and "disengaged" states might correspond to the presence or absence of some critical component of the transcriptional machinery at the gene locus; however, we remain intentionally non-committal about their physical interpretation, since these generic states are meant to capture a broad swath of potential molecular reactions. For instance, in the case of a nucleosome, the "engaged" state would correspond

to the *absence* of the nucleosome (26). The terms could also capture conformational shifts in key macromolecules such as mediator (27), or in the topology of the gene locus itself.

We assume that each component is required for transcription, such that, in a model with n molecular components only molecular states with all n components engaged are transcriptionally active, and $\mathrm{N_A} = n$ activation steps are required to achieve locus activation. Furthermore, while in reality each molecular component is likely characterized by heterogeneous dynamics (see, e.g., (5)) we again make the simplifying assumption that each molecular step is identical. As a result, it does not matter which molecular components are engaged, only how many. Figure S15B shows how this logic plays out for the case where $\mathrm{N_A} = 2$. As with the $\mathrm{N_B} = 2$ case, the model gene circuit has 8 states; however, in this case, only two states (5 and 6)—the ones in which both components are engaged—are transcriptionally active. Note that the binding and unbinding reactions connecting these states are weighted by factors of η_{ba}^2 and η_{ua}^2, respectively, to reflect the influence of each molecular factor. In general, if n components are engaged, these factors are raised to the nth power. In addition, we allow for cooperative interactions between molecular components (curved arrow in states 1,2 and 4-7), captured by the η_{aa} and η_{ia} terms in Figure S15B. In general these terms are raised to the power of $n-1$, where n is the number of engaged components at the initial molecular states.

J.3. Enforcing equilibrium constraints. In Appendix G, we outlined our approach to enforcing equilibrium constraints in the context of the simple four state gene circuits shown in Figure 1C. As outlined in Equation S57, this was done by requiring that the ration of the products of forward and backward rates around the gene circuit's single loop be set equal to 1. This, in turn, guarantees that $\phi = 0$ (see Equation 1). In general, enforcing equilibrium constraints on higher-order systems that contain multiple loops entails a significantly more complicated set of constraints. However, our assumptions (discussed in detail above) that (i) all binding reactions are identical and (ii) that all activation reactions are identical ensure that, in our case, the exact same approach holds for all higher-order models ($\mathrm{N_A} > 1$ or $\mathrm{N_B} > 1$) considered in this work.

For instance, consider the cycle containing states 3,2,6, and 7 in Figure S15A, which depicts a gene circuit with $\mathrm{N_B} = 2$ and $\mathrm{N_A} = 1$. From the rates shown in the figure, we see that the ratio of forward to backward rates in this cycle is given by

$$\lambda_1 = \frac{[c]k_b\eta_{ab}^2 k_a \eta_{ub}\eta_{ua}k_u\eta_{ib}k_i}{[c]\eta_{ba}k_b\eta_{ib}^2 k_i\eta_{ub}k_u\eta_{ab}k_a}. \qquad \text{[S59]}$$

Upon inspection, it becomes clear that this expression can be simplified dramatically by cancelling like terms in the numerator and denominator. This procedure leads to

$$\lambda_1 = \frac{\eta_{ab}\eta_{ua}}{\eta_{ba}\eta_{ib}}, \qquad \text{[S60]}$$

which is exactly equal to the expression for the four state system (Equation S57). Similar simplifications occur for all "multimodal" cycles within higher-order gene circuits, where by multimodal we mean cycles that encompass two distinct classes of molecular reactions. For instance, the cycle we considered above contains both a binding reaction *and* an activation step.

In addition to these multimodal cycles, higher order gene circuits like those shown in Figure S15A and B contain unimodal cycles that are comprised of multiple instances of the same kind of reaction. For example, consider the bottom cycle in Figure S15A that is composed of states 4-7. All four states are in the active conformation, so there are no molecular activation steps within this cycle. Instead, as we move clockwise about the loop, we see that the gene locus goes from having 0 activators bound (state 4), to 1 bound (state 5), to 2 bound (state 6), and back to 1 bound (state 7). In this case the ratio of forward and backward rate products is given by

$$\lambda_2 = \frac{[c]\eta_{ba}k_b[c]\eta_{ba}k_b\eta_{ub}\eta_{ua}k_u\eta_{ua}k_u}{[c]\eta_{ba}k_b\eta_{ub}\eta_{ua}k_u\eta_{ua}k_u[c]\eta_{ba}k_b}. \qquad \text{[S61]}$$

We find that *all* terms on the top and bottom cancel, such that

$$\lambda_2 = 1. \qquad \text{[S62]}$$

The same reasoning applies to cycles that contain multiple activation steps, rather than multiple binding events. Thus, our modeling assumptions ensure that all unimodal cycles are constrained to operate at equilibrium, irrespective of the values chosen for transition rates within the gene circuit. As a result, these cycles cannot contribute to the overall energy dissipation of the gene circuit, and do not need to be considered when applying equilibrium constraints. Thus, despite their increased molecular complexity, we find that the equilibrium constraint originally derived in the context of the simple four state case applies equally well to higher-order models.

J.4. Future directions. Throughout this work, we have treated activator binding sites and activation steps as orthogonal axes of gene circuit complexity. In reality, of course, both elements are likely at play in gene regulatory architectures. We choose to investigate the impact of each independently for two chief reasons: first it greatly simplifies exposition and allows us to more easily isolate how each aspect of gene locus architecture interacts with energy dissipation to dictate rates of information transmission. Second, since model complexity scales as $\mathrm{N_A N_B}$, we are limited in our ability to accurately explore the performance of models where both $\mathrm{N_A}$ and $\mathrm{N_B}$ are large. Improving computational and numerical techniques to permit such explorations represents an interesting future direction. We note also that such models should be tractable without need for additional development if limited to operate at equilibrium.

In addition, we wish to emphasize the potential importance of allowing for heterogeneity, both in the properties of different binding sites along the enhancer and between different molecular components within the activation pathway. This question

seems especially interesting in the context of the molecular activation steps. Our simple model with identical steps likely represents the floor of system performance. How much is to be gained when each reaction can adhere to its own kinetics, and exert a distinct kind of regulatory influence over the gene locus?

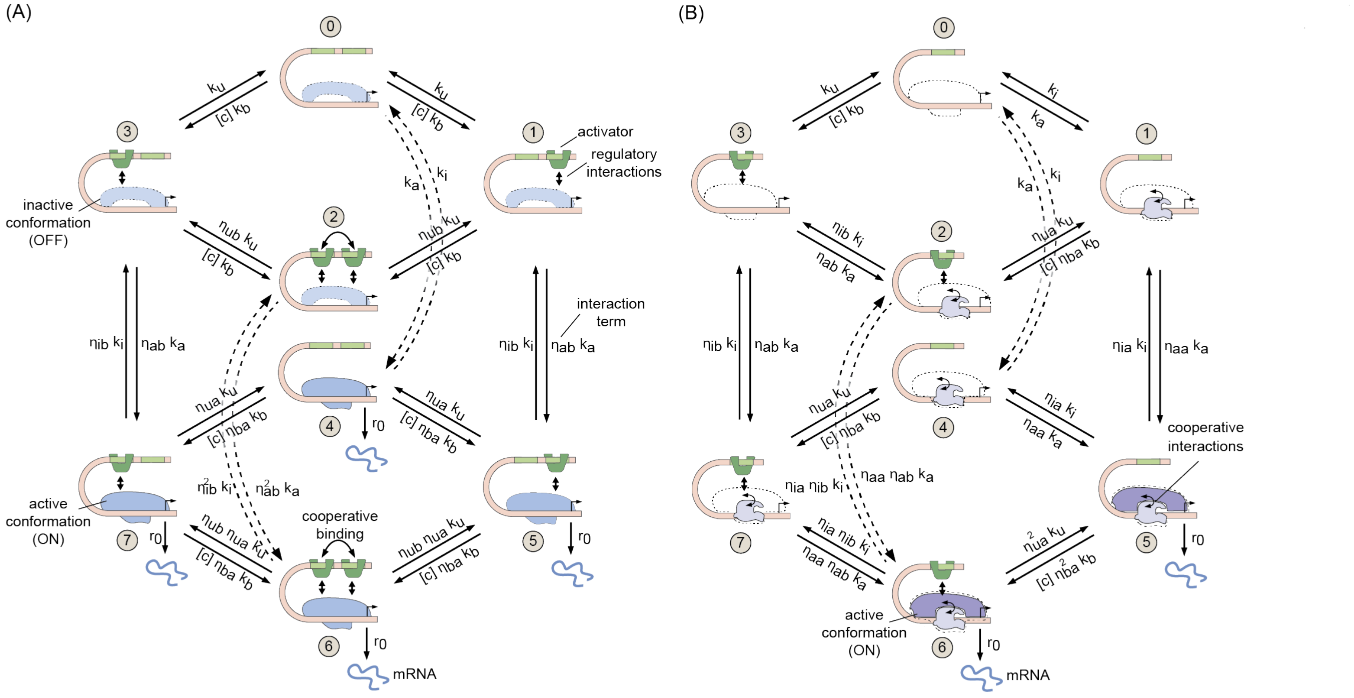

Fig. S15. Higher order gene circuit models. (A) Cartoon indicating the molecular architecture of a model gene circuit with one activation step and two activator binding sites ($N_A = 1$ and $N_B = 2$). **(B)** Molecular architecture of a model gene circuit with two activation steps and one activator binding site ($N_A = 1$ and $N_B = 2$).

K. Deriving normalized sharpness and precision metrics. In Figure 1B, the transcriptional sharpness, s, is defined as the first derivative of the transcriptional input-output function multiplied by the activator concentration, c^*, such that

$$s = \frac{dr}{dc} c^*. \quad \text{[S63]}$$

The transcriptional precision, p, is defined as the inverse of the intrinsic noise in the transcriptional input-output function:

$$p = \frac{1}{\sigma}, \quad \text{[S64]}$$

where σ is as defined in Equation S10 in the main text. Under these definitions, a key challenge in comparing sharpness and precision levels across different gene circuits is that the upper bounds on both s and p depend on the fraction of time the system spends in the transcriptionally active state, π_a (defined in Equation S8). Figure S16A and B illustrate this π_a-dependence for equilibrium and non-equilibrium realizations of the four-state system defined in Figure 1C. As an example: the equilibrium bound on s is 0.25 when $\pi_a = 0.5$, but only 0.09 when $\pi_a = 0.1$ (Figure S16A). Since we allow gene circuits to take on different transcription rates ($r = \pi_a\, r_0$) at $c = c^*$, this π_a-dependence thus confounds our efforts to understand how the molecular architecture of gene circuits—the number of binding sites, number of molecular steps, and presence or absence of energy dissipation—dictates transcriptional performance.

To overcome this issue, we need to normalize s and p such that they are independent of π_a. Focusing first on sharpness, we were inspired by previous works (18, 28) to leverage Hill Function as a flexible conceptual tool for extracting generic sharpness measures. The Hill function is defined as:

$$\pi_a = \frac{c^S}{c^S + K_d^S}, \quad \text{[S65]}$$

where c is the activator concentration, S is the Hill coefficient, and K_d is a constant that dictates the location of the function's half-max point. In general, the input-output functions generated by our model gene circuits will have more complex functional forms, but nonetheless, Equation S65 indicates that we can relate these more complex functions to the Hill function via the shared parameters π_a and c.

The sharpness of the Hill function has the form:

$$s_H = S \frac{c^S K_d^S}{(c^S + K_d^S)^2}. \quad \text{[S66]}$$

To better relate this to our input-output function, we need to re-express K_d in terms of c and π_a. Solving Equation S65 for K_d yields

$$K_d = c\left(\frac{1-\pi_\mathrm{a}}{\pi_\mathrm{a}}\right)^{\frac{1}{S}}. \qquad \text{[S67]}$$

Plugging this in to Equation S66 we obtain, after simplification:

$$s = \pi_\mathrm{a}(1-\pi_\mathrm{a})\mathrm{S}. \qquad \text{[S68]}$$

This expression tells us that the sharpness (s) of a Hill function with activity level π_a at $c = c^*$ is equal to the Hill coefficient, S, multiplied by the term $\pi_\mathrm{a}(1-\pi_\mathrm{a})$. By rearranging, we can obtain the Hill coefficient as a function of s and π_a

$$\mathrm{S} = \frac{s}{\pi_\mathrm{a}(1-\pi_\mathrm{a})}. \qquad \text{[S69]}$$

Thus, for a generic gene circuit input-output function with sharpness s and expression level π_a at $c = c^*$ we can invoke Equation S69 to calculate the Hill coefficient for the equivalently sharp Hill function (Figure S16C). This provides us with a generic measure of transcriptional sharpness that is independent of π_a and thus can facilitate comparisons across gene circuits that drive differing activity levels at $c = c^*$ (Figure S16D). We refer to this independent sharpness metric as the "normalized sharpness" in the main text, and denote it with the variable S.

This leads us to the question of transcriptional precision. The two key considerations in defining the normalized precision metric, P, are that (i) we want it to yield a quantity proportional to the information rate when multiplied with S (Equation S69), where

$$\mathrm{IR} = \left(\frac{\delta c}{c^*}\right)^2 \mathrm{S}^2\mathrm{P}^2, \qquad \text{[S70]}$$

and (ii) we want it to adhere to a single upper bound, regardless of π_a. There is only one definition that satisfies the first constraint:

$$\mathrm{P} = p\big(\pi_\mathrm{a}(1-\pi_\mathrm{a})\big) = \frac{\pi_\mathrm{a}(1-\pi_\mathrm{a})}{\sigma}. \qquad \text{[S71]}$$

Happily, Equation S71 exhibits consistent upper bounds for all π_a values, and thus satisfies our second constraint (Figure S16E).

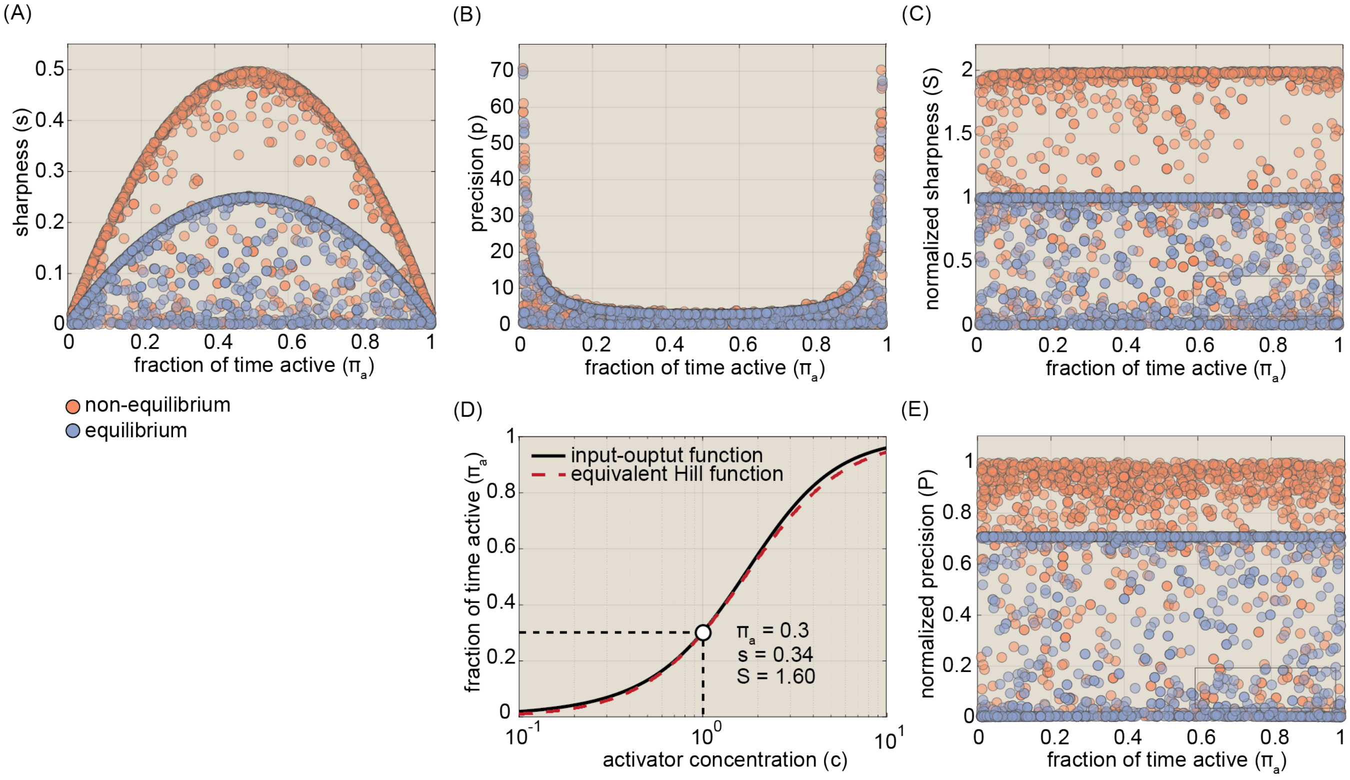

Fig. S16. Defining normalized sharpness and precision. **(A)** Plot depicting the upper sharpness limit for equilibrium (blue) and non-equilibrium (red) realizations of the four-state system depicted in Figure 1C. The upper limit depends on the fraction of time spent in the active state, π_a. **(B)** Plot of precision as a function of the transcription rate. Here again, the upper bounds depend on π_a. **(C)** Plot of normalized sharpness as a function of the transcription rate. In the case, the upper limits are invariant. **(D)** Illustration of normalized sharpness concept. For a given input-output curve, we identify normalized sharpness, S, as the Hill coefficient of an equivalently sharp Hill function with the same expression level at $C = c^*$. **(E)** On the other hand, the normalized precision, P, exhibits invariant performance bounds.

L. Optimal equilibrium four-state gene circuits behave like effective two state systems. In this section, we calculate the normalized sharpness (S) and precision (P) for a simple 2 state gene circuit (Figure S17A) with one ON state and one OFF state and two transition rates, k_{off} and k_{on}. We assume that activator binding dictates fluctuations into and out of the ON state, such that k_{on} is proportional to c ($k_{\text{on}} = ck^0_{\text{on}}$). For this simple system, the rate of transcription is given by

$$\bar{r} = r_0 \frac{ck^0_{\text{on}}}{ck^0_{\text{on}} + k_{\text{off}}} = r_0 \pi_{\text{a}}. \qquad \text{[S72]}$$

Differentiating this expression with respect to c and setting $r_0 = 1$ (as in main text), we find that

$$s = \frac{ck^0_{\text{on}} k_{\text{off}}}{(ck^0_{\text{on}} + k_{\text{off}})^2}. \qquad \text{[S73]}$$

Finally, dividing through by $b = \pi_{\text{a}}(1 - \pi_{\text{a}})$ yields the normalized sharpness, which is simply given by

$$S = 1. \qquad \text{[S74]}$$

Thus, we see that the two state model is constrained to a normalized sharpness level that represents the upper performance limit for the four-state model operating at equilibrium (blue circles in Figure 3A).

Next, we turn to precision. From Equation S10, we find that

$$\sigma^2 = \frac{2ck^0_{\text{on}} k_{\text{off}}}{(ck^0_{\text{on}} + k_{\text{off}})^3}. \qquad \text{[S75]}$$

Inverting and multiplying by b^2 gives

$$P^2 = \frac{ck^0_{\text{on}} + k_{\text{off}}}{2(ck^0_{\text{on}} + k_{\text{off}})}. \qquad \text{[S76]}$$

Finally, multiplying through by τ_b (Equation S17) and taking the square root gives

$$P = \frac{1}{\sqrt{2}}, \qquad \text{[S77]}$$

which, again, is equivalent to the upper limit of the four-state gene circuit at equilibrium (Figure 3A).

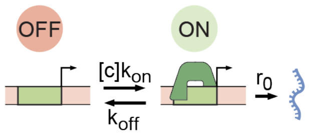

Fig. S17. A simple 2 state model of transcription. Cartoon of a simple 2 state gene circuit model in which activator binding and unbinding dictate transitions into and out of a transcriptionally active state.

M. Sharp and precise non-equilibrium networks exhibit distinct and incompatible microscopic topologies. In this appendix section, we expand upon the claim, made in Main Text Section D, that the tradeoff between sharpness and precision maximization indicated in Figure 3A reflects the fact that sharp and precise non-equilibrium gene circuits exhibit distinct and incompatible molecular architectures. One simple way to probe these differences in molecular architecture quantitatively is to measure the degree of heterogeneity (or dispersion) in (a) transition rates and (b) state probabilities. We developed entropy-based dispersion metrics ranging from 0 to 1 to quantify how uniform (0) or heterogeneous (1) transition rates and state probabilities were for different realizations of the four-state network shown in Figure 1C. While crude, these measures can provide useful microscopic insights. For instance, in gene circuits with a state probability score of 0 each microscopic state must be equiprobable ($\pi_1 = \pi_2 = \pi_3 = \pi_4 = 1/4$), while those with a 1 are maximally heterogeneous. In general, maximal heterogeneity corresponds to the case when one and only one state has a nonzero probability; however, since, for simplicity, we have elected here to focus on gene circuits where $\bar{r} = 0.5$, the maximum instead corresponds to a case when two molecular states (one OFF and one ON) have probability $\pi_i = 0.5$. Similar considerations hold for the transition rate axis. We conducted parameter sweeps to explore the space of achievable dispersion values for 10,000 non-equilibrium gene circuits (gray circles in Figure S18A).

From Figure S18A, we can see immediately that precise and sharp gene circuits occupy opposite extremes of dispersion space. Specifically, precise systems exhibit highly uniform state probability and transition rate values, while sharp networks are highly heterogeneous, both with respect to the fraction of time spent in each state and the relative magnitudes of their transition rates. These stark differences, as well as the tight clustering of each motif, suggest that sharpness and precision arise from distinct and non-overlapping microscopic architectures.

Figure S18B illustrates the sharp and precise gene circuit motifs revealed by our analysis. In maximally precise gene circuits, we find that all states are (nearly) equiprobable, all clockwise rates are nearly identical in magnitude, and all counter-clockwise rates are negligible. This leads to a clock-like system with four equal steps per cycle, a design which renders the microscopic transitions as deterministic as possible and, as a result, minimizes noise from microscopic fluctuations. In contrast, maximally sharp non-equilibrium gene circuits exhibit an effective two state architecture in which non-equilibrium driving permits activator binding to regulate *both* the locus activation step and the locus inactivation step; thereby doubling transcriptional sharpness (see Appendix N for details). Thus, maximally sharp and maximally precise non-equilibrium gene circuits require distinct molecular architectures that cannot be realized simultaneously.

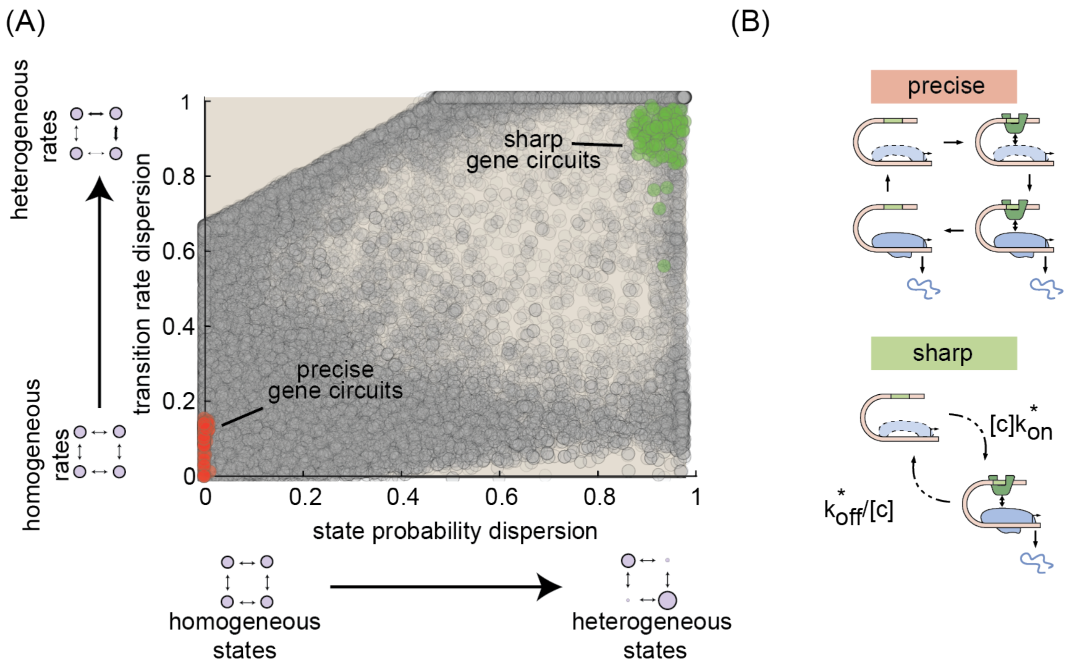

Fig. S18. Sharp and precise non-equilibrium networks exhibit distinct and incompatible microscopic architectures. (A) Plot showing dispersion scores for state probabilities and transition rates for 50,000 non-equilibrium networks. Here, a score of 0 indicates maximal uniformity (all rates or probabilities are equal) and a 1 indicates maximal heterogeneity. Green and red circles indicate the scores for the 100 gene circuits within 2% of the maximum achievable non-equilibrium sharpness and precision levels, respectively. **(B)** Cartoons illustrating distinct molecular motifs for precise and sharp non-equilibrium gene circuits. (Transition rate and interaction term magnitudes (k and η) were constrained such that $10^{-5} \leq k\tau_b \leq 10^5$ and $10^{-5} \leq \eta \leq 10^5$, where τ_b is the burst cycle time. η_{ab} and η_{ib} were further constrained such that $\eta_{ab} \geq 1$ and $\eta_{ib} \leq 1$, consistent with our assumption that the transcription factor activates the gene locus.)

To further examine the contrasting architectures of maximally precise and sharp non-equilibrium gene circuits, we used our parameter sweep algorithm to generate over 90,000 realizations of the four state system in Figure 1C, and examined the constraints that governed the steady-state probabilities of each molecular state and the magnitude of each transition rate for maximally sharp and maximally precise non-equilibrium gene circuits. We quantified the degree to which each gene circuit was optimized for precision using the following metric:

$$p_{score} = \frac{\mathrm{P}_i^2}{\mathrm{P}_{max}^2}, \quad \text{[S78]}$$

where P_i is the normalized precision of the ith gene circuit and P_{max} is the maximum achievable non-equilibrium precision (equal to 1 for this system). Note that we squared each quantity to reflect the fact that IR depends on the precision *squared* (Equation 2). To examine how the steady state probabilities (π_i) of each of the four molecular states change as gene circuits become increasingly precision-optimized, we arranged gene circuits into groups according to their p_{score}, ranging from $p_{score} = 0.5$ (the equilibrium maximum) to $p_{score} = 1$ (the non-equilibrium maximum). For each group, we calculated the distribution of π_i values. Figure S19A depicts the results of these calculations. We see that the distribution of π_i values is relatively diffuse for sub-optimal gene circuits towards the left-hand side of panels 1-4, which indicates that no single motif dominates. However, as we move to the right toward more and more precision-optimized gene circuits, we see that each of the four molecular states converge to the same π_i value of 1/4. This reflects the fact that, in maximally precise non-equilibrium gene circuits, each of the molecular states has a uniform probability.

We next undertook an analogous exercise for each of the eight transition rates that make up the four state gene circuits from Figure 1C. For this exercise, it proves to be more insightful to look at the overall magnitudes of the edges connecting states, rather than of the individual parameters laid out in Figure 1C. For example, the magnitude of the edge from state 0 to state 1 is just equal to the binding rate, $[c]k_\mathrm{b}$, but the rate going from 1 to 2 is equal to the product of two parameters: $\eta_\mathrm{ab}k_\mathrm{a}$. To

enable comparison, we normalize the transition rates according to the maximum rate in each gene circuit, such that all values should fall between 0 and 1. Figure S19B summarizes our results. We observe a tight convergence toward the maximum value of 1 for each of the four clockwise rates (panels 1-4 in Figure S19B). In contrast, we find that all counterclockwise rates (panels 5-8) are at least two orders of magnitude smaller, and follow relatively diffuse distributions, even for maximally precise gene circuits. This makes sense: as long as they are small enough relative to the clockwise rates, their precise value does not matter. Taken together, the results shown in Figure S19A and B indicate that precise non-equilibrium gene circuits exhibit highly uniform molecular architectures wherein each microscopic state is equiprobable, all clockwise transition rates are uniform, and all counterclockwise rates are negligible. This results in a "clock-like" system that maximizes the regularity of molecular transitions (Figure S18B).

Figure S19C and D show the results for a similar analysis examining the molecular architectures of sharp non-equilibrium gene circuits. Figure S19C indicates that the steady state probabilities of states 0 and 2 converge to 1/2 for maximally sharp gene circuits, while the probabilities for states 1 and 3 approach 0. This means that maximally sharp non-equilibrium gene circuits behave as effective 2 state systems. What about transition rates? The results illustrated in Figure S19D indicate that a strict of hierarchy of transition rates spanning multiple orders of magnitude is required in order to maximize transcriptional sharpness. Appendix N (below) provides further details about how the relative magnitudes of transition rates dictate non-equilibrium sharpness. Overall, we believe that the results laid out in Figure S19 confirm that maximally sharp and precise gene circuits have distinct and incompatible microscopic architectures.

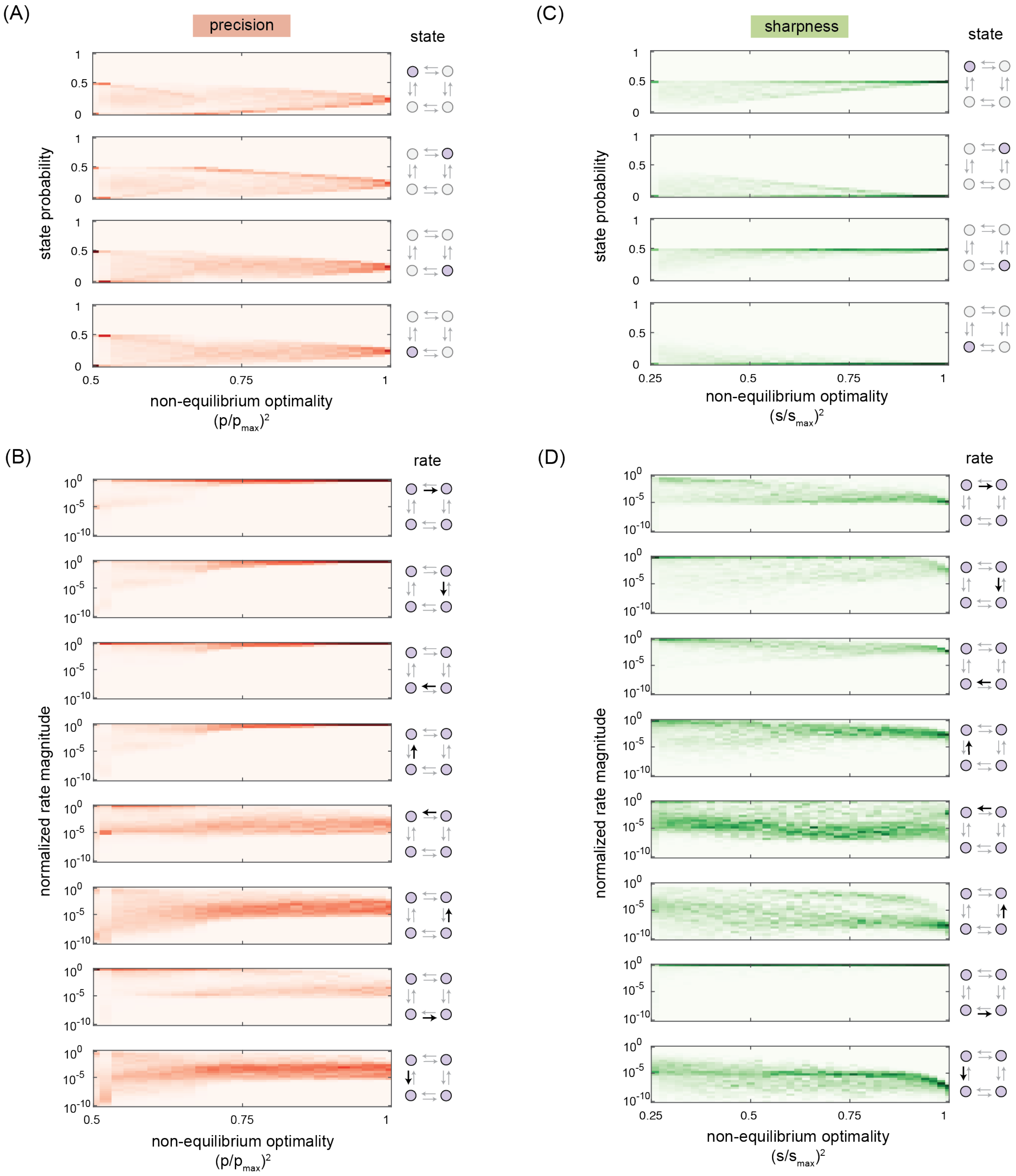

Fig. S19. A detailed dissection of sharp and precise microscopic architectures. (A) Heatmaps showing the distribution of molecular state probabilities as a function of precision optimality. Shading indicates the probability of each group. Probabilities within each column of every panel are normalized such that they sum to 1. Cartoons on the right hand side indicate which state corresponds to each panel. **(B)** Heatmaps depicting distribution of transition rate values as a function of precision optimality. Cartoons on right-hand side indicate the identity of the transition rate corresponding to each of the 8 panels. **(C)** Heatmaps showing the distribution of molecular state probabilities as a function of sharpness optimality. **(D)** Heatmaps depicting distribution of transition rate values as a function of sharpness optimality. (For the parameter sweeps used to generate these heatmaps, transition rate and interaction term magnitudes (k and η) were constrained such that $10^{-5} \leq k\tau_b \leq 10^5$ and $10^{-5} \leq \eta \leq 10^5$, where τ_b is the burst cycle time. η_{ab} and η_{ib} were further constrained such that $\eta_{ab} \geq 1$ and $\eta_{ib} \leq 1$, consistent with our assumption that the transcription factor activates the gene locus.)

N. A hierarchy of microscopic transition rates underpins non-equilibrium sharpness gain. Figure 3A shows that energy dissipation opens up a broad spectrum of S and P values that are not attainable at equilibrium. It is difficult to formulate general statements that apply to all gene circuit models inhabiting these spaces beyond the upper equilibrium limit; however, we can learn much by examining the architecture of gene circuits lying at the outer limits of non-equilibrium performance, since these systems tend to distil the logic underpinning non-equilibrium performance gains into relatively simple regulatory motifs.

Such is the case for the IR-optimized non-equilibrium four-state systems depicted as gray circles in Figure 3A. In Main Text Section D, we found that the driver of this IR is a twofold increase in sharpness relative to the upper equilibrium limit. To realize this twofold sharpness gain, we find that non-equilibrium driving is harnessed to facilitate effective one-way transitions between the active and inactive conformations—specifically, from states 1 to 2 and 3 to 0 in Figure 1C—ensuring that the system will have a strong tendency to complete transcriptional cycles in the clockwise direction ($J > 0$).

In addition to this non-equilibrium driving, sharpness maximization places strict constraints on the relative magnitudes of microscopic transition rates within the network. To understand these constraints, it is instructive to consider a coarse-grained representation of our network with a single ON state (2) and a single OFF state (0). We can obtain expressions for the two effective transition rates in the network by recognizing that they are equal to the inverse of the mean first passage times between states 2 and 0, which we can calculate using Equation S16 from Appendix A.

If we neglect the energetically disfavored transitions from 2 to 1, the effective ON rate (k^*_{on} in Figure 3D) takes on a relatively simple form

$$k_{\text{on}} = \frac{[c]k_{\text{b}}k_{\text{a}}\eta_{\text{ab}}}{[c]k_{\text{b}} + \eta_{\text{ab}}k_{\text{a}} + k_{\text{u}}}. \tag{S79}$$

From Equation S79, we see that the effective ON rate becomes proportional to the concentration, c, when the factor of $[c]k_{\text{b}}$ becomes negligible in the denominator. The limit where $k_{\text{u}} \gg [c]k_{\text{b}}, \eta_{\text{ab}}k_{\text{a}}$ represents a scenario in which the activator K_d is larger when the network is in the inactive conformation than when in the active conformation such that the activator must bind multiple times (on average) before it succeeds in driving the system into the active conformation. The other limit, when $\eta_{\text{ab}}k_{\text{a}} \gg [c]k_{\text{b}}, k_{\text{u}}$, corresponds to a system where locus activation happens rapidly upon activator binding.

In similar fashion, the effective OFF rate can be expressed as the inverse of the first passage time from 3 to 1

$$k_{\text{off}} = \frac{\eta_{\text{ua}}k_{\text{u}}k_{\text{i}}}{\eta_{\text{ba}}[c]k_{\text{b}} + k_{\text{i}} + \eta_{\text{ua}}k_{\text{u}}}. \tag{S80}$$

Interestingly, we see that the effective k_{off} becomes *inversely* proportional to c when activator binding rate exceeds both the unbinding rate and the rate of locus deactivation ($\eta_{\text{ba}}[c]k_{\text{b}} \gg k^a_{\text{u}}, k^-_{\text{off}}$). This imbalance causes the system to become kinetically trapped in the active conformation for multiple cycles of activator unbinding and rebinding, with an average duration inversely proportional to $\eta_{\text{ba}}[c]k_{\text{b}}$.

Thus, when the proper hierarchy of microscopic rates is realized, our four-state network behaves *as though* it were a two state system in which both the on and off rates are concentration dependent, such that

$$\bar{r} \approx \frac{k_{\text{on}}}{k_{\text{on}} + k_{\text{off}}} \approx \frac{[c]k^*_{\text{on}}}{[c]k^*_{\text{on}} + \frac{k^*_{\text{off}}}{[c]}}, \tag{S81}$$

where k^*_{on} and k^*_{off} are coarse-grained transition rates with units of $s^{-1}[c]^{-1}$ and $s^{-1}[c]$, respectively. Repeating the calculations from Appendix L for the above effective two state system will yield an S value of 2 and a P value of 1, in agreement with our numerical results from Figure 3A. We propose that this doubled concentration dependence can be conceptualized as a kind of "on rate-mediated" proofreading. In contrast to classical kinetic proofreading, which works by amplifying intrinsic differences in ligand off rates (29, 30), sharp networks amplify the concentration-dependence carried by binding rates, effectively "checking" C twice per cycle since both k^*_{on} and k^*_{off} are functions of the activator concentration c.

O. Non-equilibrium gains in sharpness drive IR increases in more complex regulatory architectures. This appendix section contains additional discussion relating to sharpness-precision tradeoffs for higher-order model architectures with multiple binding sites or multiple activation steps.

O.1. Sharpness maximization remains optimal for systems with multiple binding sites. To assess whether sharpness-maximization remains the optimal strategy for more complex architectures featuring multiple activator binding sites, we employed parameters sweeps to examine the space of achievable S and P values for gene circuits with 1-5 activator binding sites (and N_{B} fixed at 1). Figure S2A shows the results of this analysis. For ease of comparison across different models, we plot the relative gains in S and P for each model with respect to their maximum equilibrium values. For instance, the maximum equilibrium S value for the $\text{N}_{\text{B}} = 2$ model is 2, so a non-equilibrium gene circuit model with two binding sites that exhibits an S value of 2.5 will be calculated to have a sharpness gain of $2.5/2 = 1.25$.

Figure S2A reveals that the sharpness-precision tradeoff observed for the one-binding site model persists and, indeed, becomes more severe for systems with additional activator binding sites. We see that the non-equilibrium gain in S is fixed at approximately 2. And while the non-equilibrium gain in P increases from $\sqrt{2}$ for $\text{N}_{\text{B}} = 1$ to approximately 2.25 for $\text{N}_{\text{B}} = 5$, these P maxima (peaks in the upper left quadrant of Figure S2A) occur at lower and lower values of S, which renders them more and more disadvantageous from an IR perspective. As a result, when we plot IR-optimal gene circuits for each value of N_{B} (colored circles in Figure S2A), we find that they are invariably located in regions where $\text{S}/\text{S}_{\text{eq}} \approx 2$ and $\text{P}/\text{P}_{\text{eq}} \approx 1$. These

results demonstrate that spending energy to maximize sharpness remains the key to maximizing transcriptional information transmission, irrespective of the number of activator binding sites.

O.2. Multiple activation steps increases upper sharpness bound away from equilibrium. Figure S2B shows the range of achievable non-equilibrium gains in S and P for systems with 1-4 activation steps (and $N_B = 1$). Once again we observe a strong tradeoff between sharpness and precision, which suggests that this incompatibility is a general feature of transcriptional systems. And, once again, we find that IR-maximizing gene circuits (colored circles) lie at or near the right-most edge of achievable parameter space, indicating that dissipating energy to enhance transcriptional sharpness (rather than precision) remains the best strategy for maximizing the IR.

Yet unlike the systems examined in Figure S2A, Figure S2B reveals that the non-equilibrium gain in transcriptional sharpness (S) is not fixed but, rather, increases with the number of molecular steps from a factor of two when $N_A = 1$ to a factor of *five* when $N_A = 4$. This indicates that increasing the number of dissipative molecular steps in the activation pathway raises the upper limit on the sharpness of the transcriptional input-output function, even when the number of binding sites is held constant.

P. Specificity definitions and details. This Appendix Section uses a simple two state gene circuit model to compare and contrast the specificity definition employed in two recent works (11, 28), which compares how a single transcription factor ("TF") activates at two different gene loci (the "TF-centric" approach)—a target locus with specific binding sites, and a non-cognate locus that lacks binding site—with the definition employed in this work, which focuses on cognate and non-cognate factors competing to activate a single locus (the "gene-centric" approach).

P.1. A detailed comparison of specificity definitions for a simple 2-state model of transcription. Figure S20A illustrates the second "Tf-centric" scenario for the case of a simple two state network with a single binding site and no possibility of a conformation change at the locus; however the same idea applies equally well for the 4 state network we considered above, as well as more complicated architectures. Here transcriptional specificity is defined as the ratio of the average steady state transcription rates at on- and off-target gene loci:

$$f_{\mathrm{TF}} = \frac{\overline{r}_r}{\overline{r}_w}, \qquad \text{[S82]}$$

where f_{TF} is the specificity under the TF-centric framing of the problem, and r_r and r_w indicate the transcription rates at the cognate (right) and non-cognate (wrong) loci, respectively. In (11), the authors show that specificity for the two state system shown in Figure S20A is given by:

$$f_{\mathrm{TF}} = \frac{\alpha k_{\mathrm{u}} + [c]k_{\mathrm{b}}}{k_{\mathrm{u}} + [c]k_{\mathrm{b}}}. \qquad \text{[S83]}$$

From Equation S83, we see that the activator specificity is bounded from above by α. Moreover, this upper performance limit is achieved only in an off rate-dominated regime where $k_{\mathrm{u}} >> [c]k_{\mathrm{b}}$, which the authors in (11) note leads to a runaway increase in transcriptional noise with increasing specificity under the constraint that the mean transcription rate must remain constant. As a result, the authors conclude that non-equilibrium network architectures are necessary in order to improve specificity and minimize transcriptional noise (11).

In analogy to the parallel case outlined above, we employ a "gene-centric" definition (Figure S20B), which takes specificity as the ratio of the average number of cognate and non-cognate factors bound while the locus is in a transcriptionally productive state, normalized by concentration:

$$f = \frac{w}{c}\frac{\pi_c}{\pi_w}. \qquad \text{[S84]}$$

In the case of the two state model shown in Figure S20B, this is simply given by the ratio of fractional occupancies of states 1 and 1*:

$$f = \frac{w}{c}\frac{\pi_2}{\pi_{2^*}}. \qquad \text{[S85]}$$

Since in steady state this is necessarily at equilibrium (note the absence of cycles), we can express this ratio as a function of the difference between the energies of cognate and non-cognate factor binding, ε_c and ε_w, which leads to

$$f = \frac{w}{c}e^{-\frac{(\varepsilon_c - \varepsilon_w)}{\mathrm{k_B T}}}. \qquad \text{[S86]}$$

Next, we note that the energies can be expressed as ratios of binding and unbinding rates, such that

$$\varepsilon_c = -\mathrm{k_B T} \ln \frac{ck_{\mathrm{b}}}{k_{\mathrm{u}}} \qquad \text{[S87]}$$

and

$$\varepsilon_w = -\mathrm{k_B T} \ln \frac{wk_{\mathrm{b}}}{\alpha k_{\mathrm{u}}}. \qquad \text{[S88]}$$

Plugging these two expressions into Equation S86, we have

$$f = \frac{w}{c}e^{\ln \frac{ck_{\mathrm{b}}k_{\mathrm{u}}}{\alpha k_{\mathrm{u}} w k_{\mathrm{b}}}}, \qquad \text{[S89]}$$

which simplifies to a simple equality

$$f = \alpha. \tag{S90}$$

From Equation S90, we see that f is simply equal to the binding specificity factor α for our three state network, *irrespective of binding kinetics*. Thus, in contrast to (11), we find that equilibrium gene circuits need not shift towards a noisy, off rate-dominated regime to achieve maximum fidelity; indeed *all* systems necessarily achieve precisely $f = \alpha$. Intuitively, this difference stems from the fact that our model captures the effects of kinetic competition between cognate and non-cognate activators: whenever the cognate activator (green square in Figure S20B) is bound, non-cognate factors cannot bind.

A key limitation of this approach is that it neglects the presence of non-specific stretches of regulatory DNA, even at cognate gene enhancers. Thus, to more accurately reflect the specificity challenges faced by real gene loci, a synthesis of the two approaches summarized above will be necessary, which considers competition between cognate and non-cognate factors to bind and activate a gene locus that features both specific binding sites (which favor the cognate activator) and neutral sites (to which all activator species bind non-specifically). One expectation for such a scenario is that the simple equality stated in Equation S90 will no longer hold, and tradeoffs similar to those observed in (11) will again emerge; although, this time, the severity of these tradeoffs will depend on w/c.

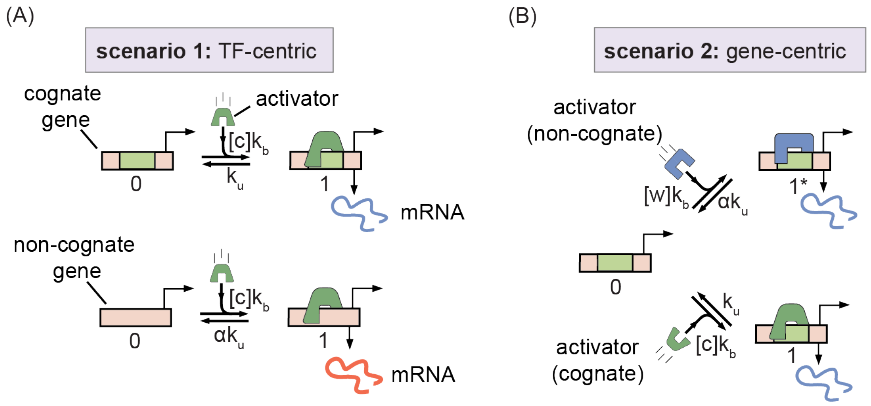

Fig. S20. Accounting for the influence of off-target activation. (A) An illustration of the parallel definition of activation fidelity. This approach considers the relative amounts of transcription driven by a transcriptional activator at its target locus and at an off target locus. **(B)** Cartoon illustrating "gene-centric" specificity definition, which considers competition between cognate and non-cognate factors to bind and activate a single gene locus.

P.2. Calculating equilibrium specificity for a gene circuit with one binding site and one activation step. Here we extend the arguments from the previous section to show that, at equilibrium, the transcriptional specificity of the six state model gene circuit shown in Figure 4A is fixed at $f^{eq} = \alpha$, irrespective of molecular details. For this system, the specificity is simply equal to the concentration-normalized ratio of the occupancies of states 2 and 4:

$$f = \frac{w}{c}\frac{\pi_2}{\pi_4}. \tag{S91}$$

Since we're assuming equilibrium conditions, we can re-express this as a difference between state energies, such that

$$f^{eq} = \frac{w}{c} e^{-\frac{(\varepsilon_2 - \varepsilon_4)}{\mathrm{k_B T}}}. \tag{S92}$$

In each case, we can express the state energies as the sum of the energy due to cognate or non-cognate factor binding with the energetic contributions from being in the active conformation, ε_{a}, and from interactions between the activator and the locus conformation, $\varepsilon_{\mathrm{ab}}$. This leads to

$$\varepsilon_2 = -\mathrm{k_B T} \ln \frac{c k_{\mathrm{b}}}{k_{\mathrm{u}}} + \varepsilon_{\mathrm{a}} + \varepsilon_{\mathrm{ab}} \tag{S93}$$

and

$$\varepsilon_4 = -\mathrm{k_B T} \ln \frac{w k_{\mathrm{b}}}{\alpha k_{\mathrm{u}}} + \varepsilon_{\mathrm{a}} + \varepsilon_{\mathrm{ab}}. \tag{S94}$$

The key is to note the the first terms on the right-hand side of the above expressions are identical to Equations S87 and S88. Since the remaining energy terms are identical, they will cancel out, such that we once again have

$$f^{eq} = \frac{w}{c} e^{\ln \frac{c k_{\mathrm{b}} k_{\mathrm{u}}}{\alpha k_{\mathrm{u}} w k_{\mathrm{b}}}}, \tag{S95}$$

which simplifies to

$$f^{eq} = \alpha. \tag{S96}$$

P.3. Calculating equilibrium specificity for gene circuits with multiple binding sites. The above arguments can be extended to apply to more complex model architectures with multiple activator binding sites. To do this, we first need to generalize the definition of specificity put forward in the main text (Equation B.2) for the case when there is more than one binding site. We define multi-site specificity as the ratio of the number of cognate and non-cognate activators bound to the gene locus (on average) while the gene is in the transcriptionally active (ON) conformation, such that:

$$f = \frac{w}{c}\frac{\langle n_c \rangle}{\langle n_w \rangle} = \frac{w}{c}\frac{\sum_{i \in \mathrm{ON}}^{N} n_i^c \pi_i}{\sum_{i \in \mathrm{ON}}^{N} n_i^w \pi_i}, \qquad \text{[S97]}$$

where $i \in ON$ stipulates that state i is part of the ON conformation, π_i is the probability of finding the gene locus in state i, and where n_i^c and n_i^w indicate the number of cognate and non-cognate factors bound to the gene locus in state i. Note also that we retain the normalizing prefactor of w/c.

Now, let's calculate f for an equilibrium gene circuit with two binding sites. Once again, we work with energies since the system is at equilibrium. From Equation S97 we see that only states with at least one cognate or non-cognate factor bound contribute to the numerator and denominator, respectively. As a result, in each case, there are just three distinct molecular states to consider. For the cognate case (numerator), these are 1 cognate bound and 0 non-cognate, 1 cognate and 1 non-cognate, and 2 cognate. The non-cognate case (denominator) follows the same pattern. Drawing from the expression in the previous section, this leads to

$$f^{eq} = \frac{w}{c}\frac{\frac{ck_{\mathrm{b}}}{k_{\mathrm{u}}}\eta_a\eta_{ab} + 2\frac{ck_{\mathrm{b}}}{k_{\mathrm{u}}}\frac{wk_{\mathrm{b}}}{\alpha k_{\mathrm{u}}}\eta_a\eta_{ab}^2\eta_{ub} + 2\left(\frac{ck_{\mathrm{b}}}{k_{\mathrm{u}}}\right)^2\eta_a\eta_{ab}^2\eta_{ub}}{\frac{ck_{\mathrm{b}}}{\alpha k_{\mathrm{u}}}\eta_a\eta_{ab} + 2\frac{ck_{\mathrm{b}}}{k_{\mathrm{u}}}\frac{wk_{\mathrm{b}}}{\alpha k_{\mathrm{u}}}\eta_a\eta_{ab}^2\eta_{ub} + 2\left(\frac{ck_{\mathrm{b}}}{\alpha k_{\mathrm{u}}}\right)^2\eta_a\eta_{ab}^2\eta_{ub}}, \qquad \text{[S98]}$$

where η_a is a weight factor corresponding to the active conformation ($\eta_a = e^{-\frac{\varepsilon_a}{k_{\mathrm{B}}T}}$), η_{ab} is a weight factor capturing cooperative interactions between the bound activator and the active conformation, and η_{ub} captures cooperative interactions between bound activator molecules. Note that the three terms in the numerator and denominator of Equation S98 match the ordering of the scenarios given above the equation. Factoring out common multipliers leads to

$$f^{eq} = \frac{w}{c}\frac{\frac{ck_{\mathrm{b}}}{k_{\mathrm{u}}}}{\frac{wk_{\mathrm{b}}}{\alpha k_{\mathrm{u}}}}\frac{1 + 2\frac{wk_{\mathrm{b}}}{\alpha k_{\mathrm{u}}}\eta_{ab}\eta_{ub} + 2\frac{ck_{\mathrm{b}}}{k_{\mathrm{u}}}\eta_{ab}\eta_{ub}}{1 + 2\frac{ck_{\mathrm{b}}}{k_{\mathrm{u}}}\eta_{ab}\eta_{ub} + 2\frac{ck_{\mathrm{b}}}{\alpha k_{\mathrm{u}}}\eta_{ab}\eta_{ub}}, \qquad \text{[S99]}$$

where we now see that the numerator and denominator are identical in the right-most ratio. Thus, we find that

$$f^{eq} = \alpha. \qquad \text{[S100]}$$

Similar patterns repeat for systems with more binding. See the Mathematica notebook entitled "specificity_multi_site.nb" in this paper's git repository (https://github.com/nlammers371/noneq-gene-regulation.git) for a full treatment of the 3 and 5 binding site cases.

Q. Deriving non-equilibrium tradeoff bound between intrinsic sharpness and specificity. In this section, we lay out the key steps in deriving the non-equilibrium tradeoff bound between sharpness and specificity depicted as a black dashed line in Figure 5B, which has the form:

$$\mathrm{S} \leq \underbrace{\frac{f}{\frac{w}{c} + f}}_{\substack{\text{specificity} \\ \text{factor } (p_c)}} \times \underbrace{\left(\frac{\alpha^2 + \alpha f - 2f}{\alpha f - f}\right)}_{\substack{\text{intrinsic} \\ \text{sharpness } (\mathrm{S}_0)}}, \qquad \text{[S101]}$$

where we assume that $\alpha \leq f \leq \alpha^2$.

To arrive at this bound, we make use of insights gained in Appendix N, where we used first passage times to examine the key microscopic conditions for the twofold gain in sharpness away from equilibrium observed in Figure 3A. Even for the simple six state system illustrated in Figure 4A, our system has eight degrees of freedom when operating away from equilibrium. As such, a key part of our approach will be to first reduce this complexity as much as possible while preserving the salient behaviors, namely the possibility for non-equilibrium gains in sharpness and specificity. After this, we identify a tuning parameter, β, that can be used to interpolate between maximally sharp to maximally specific non-equilibrium gene circuit architectures. Since the expressions for non-equilibrium gene circuits are, in general, quite complex, we sketch the key steps here and direct the reader to the Mathematica notebook entitled "sharpness_specificity_bound_derivation.nb" on the project git repository for additional details: https://github.com/nlammers371/noneq-gene-regulation.git. Note that we work in units of c throughout, such that $c = 1$.

To begin, we strip unnecessary dimensions from our system. We set $\eta_{\mathrm{ib}}k_{\mathrm{i}}$ and k_{a} to the same generic rate, k_1. Next, we set $\eta_{\mathrm{ab}}k_{\mathrm{a}}$, $\eta_{\mathrm{ua}}k_{\mathrm{u}}$, and k_{b} to a second rate parameter, k_2. Finally, we set k_{i} equal to $\beta\eta_{\mathrm{bs}}k_{\mathrm{b}}$, where β is our interpolation parameter. This leaves us with a system with five free parameters, rather than eight.

In Appendix N, we saw that maximally sharp non-equilibrium gene circuits (i) only switch into the active transcriptional conformation when the activator is bound and (ii) only switch *out* of the ON states when the activator is unbound. This

amounts to effective one-way transitions from states $1 \rightarrow 2$ (equivalently, $5 \rightarrow 6$) and $3 \rightarrow 1$. We impose this condition by taking the limit where $k_1 \rightarrow 0$. Next, we impose the condition uncovered by examination of Equation S79:

$$k_{\mathrm{u}} \gg [c]k_{\mathrm{b}}, \eta_{\mathrm{ab}}k_{\mathrm{a}}, \tag{S102}$$

by taking the limit where k_{u} approaches infinity.

These limits lead to a further simplified system that can be used to investigate fundamental tradeoffs between intrinsic sharpness and specificity. For this stripped-down system, we find that the expression for specificity, f, is quite simple:

$$f = \frac{\alpha(\alpha + \alpha\beta + w)}{\alpha + \beta + w}, \tag{S103}$$

where we see that all dependence on microscopic transition rates has dropped out, with the exception of our interpolation parameter, β. Furthermore, tuning β causes Equation S103 to shift from equilibrium levels ($f = \alpha$ when $\beta = 0$) to the non-equilibrium limits revealed by Figure 5B ($f = \alpha^2$ when $\beta \gg \alpha, w$).

The normalized sharpness, S, has a slightly more complicated functional form, given by

$$S = \frac{\alpha\left[\alpha(k_2 + (2+\beta)\eta_{\mathrm{ba}}k_{\mathrm{b}}) + 2\eta_{\mathrm{ba}}k_{\mathrm{b}}w\right]}{\alpha^2(k_2 + \eta_{\mathrm{ba}}k_{\mathrm{b}} + \beta\eta_{\mathrm{ba}}k_{\mathrm{b}}) + \alpha w(k_2 + 2\eta_{\mathrm{ba}}k_{\mathrm{b}}) + w\eta_{\mathrm{ba}}k_{\mathrm{b}}(\beta + w)}. \tag{S104}$$

To obtain an expression for the intrinsic sharpness, S_0, we divide through by the specificity prefactor (p_c) from Equation B.1:

$$\mathrm{S}_0 = \frac{f + \frac{w}{c}}{f} S. \tag{S105}$$

Simplifying and applying the condition that $k_2 \approx 0$ leads to

$$\mathrm{S}_0 = 2 - \frac{\alpha\beta}{\alpha + \alpha\beta + \frac{w}{c}}. \tag{S106}$$

Here again, as with Equation S103, we see that all dependence on the on rate parameters drops away. Further, it is easy to see that this expression goes to 2 when $\beta = 0$ and 1 when $\beta \gg \alpha, w$. Thus, when β is small, our system exhibits equilibrium levels of specificity and non-equilibrium levels of intrinsic sharpness and, when β is large, it exhibits non-equilibrium specificity and equilibrium sharpness levels. Thus, we have succeeded in our initial aim to establish a simplified model that can capture the tradeoffs between sharpness and specificity revealed by our numerical parameter sweeps (Figure 5B).

As a final step, we can solve Equation S103 to obtain an expression for β in terms of f:

$$\beta = \frac{(f - \alpha)(\alpha + w)}{\alpha^2 - f}. \tag{S107}$$

Plugging this expression into Equation S105 and simplifying yields an expression for S_0 as a function of f:

$$\mathrm{S}_0 = \frac{\alpha^2 + \alpha f - 2f}{\alpha f - f}, \tag{S108}$$

where we assume that $\alpha \leq f \leq \alpha^2$. Thus, we have obtained the final S_0 expression depicted in Equation S101. Observe that $\mathrm{S}_0 \approx 2$ when $f = \alpha$ and $\mathrm{S}_0 \approx 1$ when $f = \alpha^2$. Equation S108 gives the dashed black curve bounding f vs. S_0 sweep results shown in Figure 5B, confirming that it represents the limiting behavior of intrinsic sharpness and specificity for non-equilibrium realizations of the six state model from Figure 4B.

References

1. AH Lang, CK Fisher, T Mora, P Mehta, Thermodynamics of statistical inference by cells. *Phys. Rev. Lett.* **113** (2014).
2. JL Lebowitz, H Spohn, A gallavotti-cohen-type symmetry in the large deviation functional for stochastic dynamics. *J. Stat. Phys.* **95**, 333–365 (1999).
3. TL Hill, *Free Energy Transduction and Biochemical Cycle Kinetics.* (Springer New York), p. 119 (1989).
4. W Whitt, Asymptotic Formulas for Markov Processes with Applications to Simulation. *Oper. Res.* **40**, 279–291 (1992).
5. NC Lammers, YJ Kim, J Zhao, HG Garcia, A matter of time: Using dynamics and theory to uncover mechanisms of transcriptional bursting (2020).
6. CJ Geyer, Introduction to Markov Chain Monte Carlo, Technical report (2011).
7. DT Gillespie, Exact stochastic simulation of coupled chemical reactions in *Journal of Physical Chemistry.* (American Chemical Society), Vol. 81, pp. 2340–2361 (1977).
8. FJ Massey, The Kolmogorov-Smirnov Test for Goodness of Fit. *Source: J. Am. Stat. Assoc.* **46**, 68–78 (1951).
9. ED Siggia, M Vergassola, Decisions on the fly in cellular sensory systems. *Proc. Natl. Acad. Sci.* **110**, E3704–E3712 (2013).
10. TM Cover, JA Thomas, *Elements of Information Theory 2nd Edition (Wiley Series in Telecommunications and Signal Processing).* p. 776 (2006).

11. R Shelansky, H Boeger, Nucleosomal proofreading of activator–promoter interactions. *Proc. Natl. Acad. Sci. United States Am.* **117**, 2456–2461 (2020).
12. NC Lammers, et al., Multimodal transcriptional control of pattern formation in embryonic development. *Proc. Natl. Acad. Sci.* **117**, 836–847 (2020).
13. K Tantale, et al., A single-molecule view of transcription reveals convoys of RNA polymerases and multi-scale bursting. *Nat. Commun.* **7**, 12248 (2016).
14. A Wald, Sequential Tests of Statistical Hypotheses. *The Annals Math. Stat.* **16**, 117–186 (1945).
15. A Wald, J Wolfowitz, Optimum Character of the Sequential Probability Ratio Test. *The Annals Math. Stat.* **19**, 326–339 (1948).
16. J Desponds, M Vergassola, AM Walczak, A mechanism for hunchback promoters to readout morphogenetic positional information in less than a minute. *eLife* **9** (2020).
17. E Eck, et al., Quantitative dissection of transcription in development yields evidence for transcription factor-driven chromatin accessibility. *bioRxiv* p. 2020.01.27.922054 (2020).
18. J Estrada, F Wong, A DePace, J Gunawardena, Information Integration and Energy Expenditure in Gene Regulation. *Cell* **166**, 234–44 (2016).
19. CH Lee, H Shin, J Kimble, Dynamics of Notch-Dependent Transcriptional Bursting in Its Native Context. *Dev. Cell* **50**, 426–435.e4 (2019).
20. EJA Hubbard, The C. elegans germ line: a model for stem cell biology. *Dev. dynamics : an official publication Am. Assoc. Anat.* **236**, 3343 (2007).
21. HG Son, et al., RNA surveillance via nonsense-mediated mRNA decay is crucial for longevity in daf-2/insulin/IGF-1 mutant C. elegans. *Nat. Commun. 2017 8:1* **8**, 1–11 (2017).
22. JE Pérez-Ortín, P Alepuz, S Chávez, M Choder, Eukaryotic mRNA Decay: Methodologies, Pathways, and Links to Other Stages of Gene Expression. (2013).
23. VE Foe, BM Alberts, Studies of nuclear and cytoplasmic behaviour during the five mitotic cycles that precede gastrulation in Drosophila embryogenesis. *J. cell science* **61**, 31–70 (1983).
24. BJ Vincent, J Estrada, AH DePace, The appeasement of Doug: a synthetic approach to enhancer biology. *Integr. Biol.* **8**, 475–484 (2016).
25. M Erokhin, Y Vassetzky, P Georgiev, D Chetverina, Eukaryotic enhancers: common features, regulation, and participation in diseases. *Cell. molecular life sciences : CMLS* **72**, 2361–2375 (2015).
26. LA Mirny, Nucleosome-mediated cooperativity between transcription factors. *Proc. Natl. Acad. Sci. United States Am.* **107**, 22534–22539 (2010).
27. E Nogales, RK Louder, Y He, Structural Insights into the Eukaryotic Transcription Initiation Machinery. *Annu. Rev. Biophys.* **46**, 59–83 (2017).
28. R Grah, B Zoller, G Tkačik, Nonequilibrium models of optimal enhancer function. *Proc. Natl. Acad. Sci. United States Am.* **117**, 31614–31622 (2020).
29. JJ Hopfield, Kinetic Proofreading: A New Mechanism for Reducing Errors in Biosynthetic Processes Requiring High Specificity (protein synthesis/DNA replication/amino-acid recognition). **71**, 4135–4139 (1974).
30. J Ninio, Kinetic amplification of enzyme discrimination. *Biochimie* **57**, 587–95 (1975).
